# Medulloblastoma: biology and immunotherapy

**DOI:** 10.3389/fimmu.2025.1602930

**Published:** 2025-07-03

**Authors:** Alessandro Poggi, Francesco Reggiani, Helena S. Azevedo, Lizzia Raffaghello, Rui Cruz Pereira

**Affiliations:** ^1^ Molecular Oncology and Angiogenesis Unit, IRCCS Ospedale Policlinico San Martino, Genoa, Italy; ^2^ Gene Expression Regulation Unit, IRCCS Ospedale Policlinico San Martino, Genoa, Italy; ^3^ Instituto de Investigação e Inovação em Saúde, Universidade do Porto, Porto, Portugal; ^4^ INEB - Instituto de Engenharia Biomédica, Universidade do Porto, Porto, Portugal

**Keywords:** medulloblastoma, adaptive and innate immune response, Tumor microenvironment, brain tumor, unconventional t cells, NK cells, immunotherapy

## Abstract

Medulloblastoma is an aggressive central nervous system tumor affecting children more commonly between the ages of 5-9. It is usually localized in the cerebellum, leading to diffusion of tumor cells through the cerebrospinal fluid and metastases to other portions of the brain and spinal cord. Conventional treatment consists of surgical resection followed by adjuvant radiation and/or chemotherapy. The side effects of these therapies are critical to consider, especially given that patients are in a distinct stage of their lives. In addition, the overall survival is not satisfactory ranging from 50-90% depending on the type of medulloblastoma. The molecular characterization has broadly subdivided medulloblastoma into four subgroups, and more recently, the single-cell transcriptomics studies have further identified several other subgroups. Important advances have been reported on the cell origin, their plasticity, heterogeneity of genetic and epigenetic alteration, and interaction with the immune and stromal components of the tumor microenvironment. Research studies on these key points are essential to make advances in planning the application of conventional therapies together with immunotherapies. Herein, we discuss the main advances recently obtained on medulloblastoma biology and immunotherapies. Overall, the biological and molecular features of medulloblastoma are briefly summarized to understand the reason for the application of the old and new immunotherapies. Immunotherapies considered include the identification of potential medulloblastoma neoantigens and tumor-associated antigens to generate antigen-specific T lymphocytes. The main antigens expressed by medulloblastoma cells and/or by components of the tumor microenvironment will be considered as the molecular targets of antibodies, antibody derivatives, and chimeric antigen receptor effector cells to improve the conventional therapies. In the last portion of this review, the brief analysis of the activating and inhibiting receptors expressed by antitumor T, natural killer, and unconventional T cells can give new insights into the potential treatment of medulloblastoma.

## 1 Introduction

Medulloblastoma (MB) is the most common embryonal malignant tumor of the central nervous system (CNS) in children, reaching about the 20% of all brain pediatric tumors. Large cohorts of patients underwent comprehensive omics analyses (genomic, transcriptional, proteomic, methylomic and epigenetic changes), resulting in the definition of four consensus molecular subgroups (1). These subgroups display differences in patient demographics, pathogenesis, prognosis and response to therapy. World Health Organization (WHO) criteria now integrate these molecular findings with the traditional histology classification (2). The four subgroups have been defined as Wingless and Int-1 (WNT)-activated, Sonic Hedgehog (SHH)-activated, Group 3 (non-WNT/non-SHH), and Group 4 (non-WNT/non-SHH), and they have been well characterized in other reviews and herein will be briefly considered thereafter (1, 2). The overall 5-year survival for MB can reach 70-85%, but the toxic effects of surgery, chemotherapy and radiation have long-term consequences for pediatric patients. It is necessary to identify more precision therapies to reduce the morbidity of treatments. The conventional treatments consisting of surgical resection, chemotherapy and radiation can lead to relevant drawbacks related to the age of the patient. Indeed, the major severe adverse effects comprise neuroendocrine dysfunction, growth alterations, infertility, neurocognitive disabilities and even secondary malignancies. The surgical intervention is the first line of treatment, and it tends to eliminate as much tumor mass as possible without causing more signs and symptoms. Following the surgery, the irradiation of the brain and spine with a proton beam is necessary as the MB tends to diffuse from the original site to the rest of the brain and spine. The reduction or omission of radiotherapy can result in an ineffective treatment, although this reduces the above-mentioned side effects (3, 4). Overall, the treatment will be chosen based on the subtype, its diffusion, patient response, associated side effects and quality of life, emphasizing the trade-off between survival and neurocognitive disabilities (5). Chemotherapy increases the survival of patients, but it is not effective in many cases as the 5-year overall survival (OS) can reach about 70% in patient in the high-risk group (6).

The immune system is involved in the control of tumor cell growth as it can sense the alterations present in tumor cells due to genetic mutations, which may lead to the expression of tumor neoantigens, tumor-associated antigens or stress molecules (7–11). By consequence, the immune system can react against tumors, both eliciting an adaptive and innate immune response (12–14). The recognition of tumor cells, can lead to their killing. The recent literature is full of reports that claimed the key role of immune system-mediated control of tumor cell growth (15–17). It has been reported in 1985 that lymphokine-activated killer (LAK) cells were efficient in some tumors, such as melanoma (18–20). This was one of the first clear experimental proofs of the concept that the immune system can check tumor growth. Since that discovery, the following clinical applications of infusing high doses of interleukin (IL)2 to treat human melanoma or renal cell carcinoma gave contradictory results in terms of effectiveness. More importantly, the identification of life-threatening IL2-mediated side effects in humans such as vascular leakage syndrome (VLS) limited its clinical applications (21, 22). This stimulated researchers to better define the modalities of administration of IL2 and the use of IL2 derivatives instead of IL2 itself (23–25). Today, several clinical trials have shown that an immune response can be elicited by stimulating T lymphocytes, relieving the brake of autoreactivity using immune checkpoint inhibitors (ICI) and/or chimeric antigen receptor (CAR) engineered T or natural killer (NK) cells. The incoming approaches to block tumor cell growth and resistance will use a combo of targeted therapy together with conventional treatments (26–29). These treatments show important side effects such as cytokine release syndrome (CRS), immune effector cell-associated neurotoxicity syndrome (ICANS) for CAR-T cells or serious autoimmune reactions in the lungs, intestines, liver, hormone-making glands, kidneys, or other organs for ICI (30–34). However, the effective therapeutic responses observed with some types of tumors, such as hematological malignancies, melanoma and non-small cell lung carcinomas, justify the use of these antitumor biological drugs (35–37). Conceivably, these new therapeutic approaches can be applied to treat MB, limiting the toxic side effects of conventional therapies (38, 39). Herein, we will analyze in some detail the biological features of MB cells together with the molecular targets for triggering an efficient immune response, analyzing the rationale of using unconventional therapeutic approaches and suggesting new treatments for this pediatric tumor.

## 2 Epidemiology and biological features of medulloblastoma

Briefly, the epidemiology and biological features of MB will be considered to give an overall scenario in which the old and new immunotherapies can be applied. The main point to remark is that pediatric patients are primarily involved by this tumor. Pediatric patients have two relevant characteristics, among others, to be considered when immunotherapies are applied: 1- the immune response in these patients can be different from an adult; the immune system is developing; 2- the MB arises in the cerebellum in which are developing interactions with other portion of the brain to allow the coordination of the large majority of motor neuron functions and cognitive properties (40–42).

The designation “medulloblastoma” was first named in 1925 by neurosurgeons Bailey and Cushing, and reflects its anatomical origin in the posterior fossa (43). As the most frequent malignant tumor among pediatric CNS cancers, it accounts for approximately 60% of intracranial embryonal tumors. It arises in the cerebellum, a hindbrain structure responsible for motor coordination and learning, situated at the base of the brain near the fourth ventricle (44, 45). While most MBs occur sporadically, a subset is linked to genetic predisposition syndromes, notably within the SHH-activated subgroup (46, 47). These syndromes include Li-Fraumeni syndrome (TP53 mutations), Turcot syndrome (APC-associated polyposis), Fanconi anemia subtypes, and Gorlin syndrome (nevoid basal-cell carcinoma syndrome) (46, 47). According to the CBTRUS report, MB is the most common malignant childhood CNS tumor, comprising the largest percentage of embryonal tumors (70.2%) and almost 20% of all pediatric brain tumors (48).

While primarily a childhood disease (approximately 10 cases per million children), it is rare in adults (approximately 0.54 cases per million). Pediatric and adult MBs exhibit distinct molecular profiles, with pediatric tumors typically harboring fewer genetic mutations in comparison with the adults (44, 49, 50). Notably, pediatric cases show a sex disparity; males are more likely to develop than females, suggesting biological sex as a potential risk factor. However, susceptibility also varies based on tumor histology and molecular subtypes, which makes it even more difficult to establish a predefined grade of risk factors (44–46).

Pediatric MB is often metastatic at the time of initial diagnosis, with a tendency to spread beyond the CNS via cerebrospinal fluid (CSF), lymphatic circulation, and the bloodstream. While external CNS metastasis is a rare feature in most brain tumors, it occurs more frequently in MB compared to other pediatric CNS tumors (44, 51, 52). External CNS metastases typically emerge later and are less commonly detected with metastasis, including bones, bone marrow and to a lesser extent, lymph nodes, liver and lungs (51, 52). This metastatic potential raises special concerns as it appears to be associated with a higher likelihood of progression to a specific subtype, indicating a poor prognosis. The aggressive nature of MB, coupled with its early metastatic behavior, underscores the challenges in managing this malignancy and highlights the need for early detection and targeted therapeutic strategies.

### 2.1 Classification and biology

Similar to other cancers, MB presents a high heterogeneity, both in terms of histology and molecular characteristics. Advances in tumor biology and genetics have led to a classification system that improves both diagnosis and treatment strategies. Originally, MB was categorized by histological properties into three main subtypes: classic (C), desmoplastic/nodular (DN), and large cell/anaplastic (LCA) depending on cellular phenotype (53–57).

The classic MB group represents the most common subtype, accounting for 66–72% of cases. Tumoral cells typically do not grow significantly in volume and rarely show structural alterations such as desmoplasia or nodules exhibiting a high nucleus-to-cytoplasm ratio, rounded nuclei, and significant mitotic and apoptotic activity. The DN group is much less frequent than the classic subtype, representing about 15% of all the cases. It is often associated with a favorable prognosis and a distinct molecular subtype (SHH), facilitating a quicker and more precise diagnosis. MB cells have the ability to deposit collagen in the pericellular space. Cytologically, these cells appear small and round, with characteristic arborizations and pericellular reticulin deposition. This subtype of MB tumors presents a specific variant - MB with Extensive Nodularity (EN) – that primarily occurs in newborns (50, 55). The LCA subtype accounts for approximately 15% of the cases that have more aggressive behavior. It is characterized by nuclear pleomorphism, increased cell volume, and a tendency for cells to cluster. Within this subgroup, the large cell variant is particularly rare, occurring in only 2–4% of cases. Cells show high proliferative and apoptotic activity and are associated with a significantly poorer prognosis compared to other subtypes (54–57).

**Figure 1:**
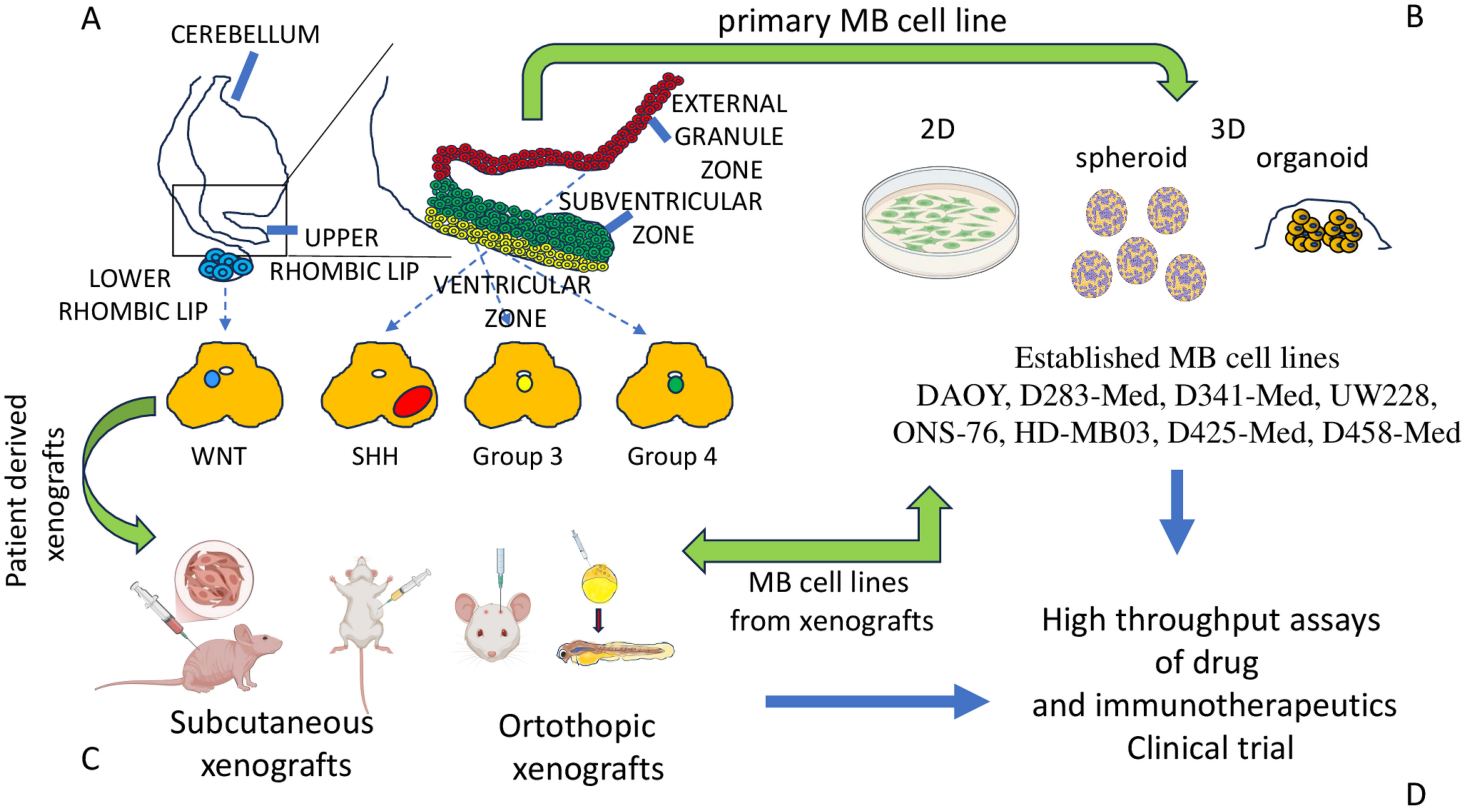
MB molecular subgroups and models for studying the biology of MB cells. **(A)** Four main subgroups of MB have been proposed on the basis of OMICS analysis. MB cells derive from different regions of the cerebellum, as shown. **(B)** The major models to study the biology of MB cells are represented by primary cell lines derived from tumor specimens of patients. Some of them can be stabilized during the culture, maintaining specific phenotypic and functional features leading to established cell lines (some of which have been listed). The primary and/or well-established cell lines can be cultured in conventional (inappropriately called 2D culture) or 3D conditions, such as spheroid or organoid. **(C)** Patients’ tumor specimens or cell lines can be inoculated subcutaneously or orthotopically in small animals (mice/rats) with an impaired immune response to allow the engraftment of these xenografts. Also, MB cells from patients or cell lines can be inoculated in blastula of Zebrafish to generate orthotopic models of MB tumor. The animal models (subcutaneously or orthotopic models) are a key tool to study the growth and metastatic behavior of MB cells. From xenografts, cell lines can be obtained for *in vitro* studies. **(D)** Altogether, these research models are the basis for the selection of novel drugs and immunotherapeutic tools to be validated in clinical trials.

The 2021 fifth edition of the WHO Classification of CNS Tumors categorizes MBs into four distinct molecular subgroups: WNT-activated, SHH-activated with TP53 wild type, SHH-activated with TP53 mutation, and non-WNT/non-SHH (formerly classified as groups 3 and 4 (50, 58–60). (**Figure 1**). These subcategories are further segmented into more specific subtypes using advanced methylation profiling, which has identified multiple WNT-, SHH- and non-WNT/non-SHH-associated subtype. This molecular stratification has significantly enhanced the understanding of the biological diversity and clinical variability of MB, which enables more precise classification, prognosis and therapy (57–70). Information about epidemiological, clinical and molecular features of MB molecular subcategories is summarized in **Table 1**. It is to be noted that the identification of distinct methylation patterns and histological features continues to drive research into targeted therapies and personalized medicine treatment protocols, aiming to improve outcomes for patients with MB (61, 62). Furthermore, besides the sporadic medulloblastoma, this tumor can occur in association with cancer predisposition syndromes such as colon polyps in Turcot syndrome or basal-cell carcinomas in Gorlin syndrome [reviewed in (46, 47, 73, 74)]. This event should be considered in relation to each molecular subgroup and this knowledge can guide oncologist to perfom the cancer surveillance to diagnose and treat early the medulloblastoma in collaboration with other specialists. This topic is of great relevance for MB as germline mutations are 5-6% and these genetic alteration affect specific molecular pathways leading to tumor development (75).

**Table 1:** Main features of medulloblastoma tumor.

Characteristic	Medulloblastoma molecular subtype			
	WNT	SHH	Group 3	Group 4
Prevalence %	<5-10	20-30	20-25-40-50	35-40
Female/male ratio	1:1	1:1	2:1	2:1 3:1
Age	Children, teens	Infants, adults	Infants, children	Infants, children, adults
Histology	Classic	Nodular/Desmoplastic, Classic, LCA	Classic, LCA	Classic, LCA
Cells of origin	Lower rhombic	Cerebellar granule precursors of neuron	Hypothesis of neural stem cells	Upper rhombic leap progenitors
Metastatic strenght	low	low	high	high
Recurrence	rare	local	metastasis	metastasis
Prognosis	good	middle	poor	middle
Risk level	Low risk	TP53WT intermediate, TP53 mutant very high	Intermediate to high	intermediate
5-years survival %	95	75	50	75
Genetic alteration	WNT	SHH	Group 3	Group 4
Somatic mutation	CTNNB1, DDX3X, SMARCA4, TP53, CSNK2B, PIK3CA, KMTD2, CREBBP	PTCH1, SMO, TP53, SUFU, DDX3, TERT, IDH1, KMT2D, BCOR	SMARCA4, CTDNEP1, KMT2D, KBTBD4, MLL2	KDM6A, KMT2C, ZMYM3, KBTBD4, MLL3
Germline mutation	APC (<5%) CREBBP	PTCH1, TP53, SUFU		
Chromosome affected Copy number	Ch 6 loss monosomy	Ch 3 gain, Ch9p gain, Ch 9q loss, Ch 10q loss Ch 17p loss TERT, MYCN, GLI1/2	Ch 1 gain, Ch 5 loss, 10q loss, MYC, PVT1, OTX2, GF11/1B, GF11 overexpression	IsoCh 17 Ch X loss, Ch 17p loss PRDM6, SNCAIP, MYCN, CDK6, GF11 and GF11B

The table has been generated using information from references 1, 2, 67–72. Please note the high variability in the prevalence are reported in the references cited.

### 2.2 MB cell lines models

Like in other cancers, despite advancements in multimodal therapy, MB still remains a challenge due to its high heterogeneity and therapy resistance. To better understand biology and develop targeted therapies, researchers rely on well-characterized cell lines as essential tools for studying tumor pathogenesis, investigating signaling pathways, and screening therapeutic compounds (**Figure 1**). Several cell lines have been established, each representing different molecular subtypes and biological characteristics. Herein, we present a brief description of the most used cells, which include DAOY, D283-Med and D341-Med, UW228, ONS-76, HD-MB03 and D425-Med and D458-Med (76, 77) (**Figure 1**, **Table 2**).

**Table 2:** Features of some MB cell lines.

Cell line	DAOY	UW228	ONS-76	HD-MB03	D425-Med	D341-Med	D283-Med	D458-Med
Subtype	SHH (DN)	SHH	SHH (DN)	Group 3	Group 3	Group 3	Group 3/4	Group3/4
Growth Type	Adherent and Suspension	Adherent (epithelial like)	Adherent (differentiation with RA)	Suspension	Suspension	Suspension	Suspension	Suspension
Key Alterations	PTCH1 mutation, 9q loss, 10q gain (MYCN), WT TP53	PTCH1 mutation, 9q loss, WT TP53	PTCH1 deletion/mutation, WT TP53	MYC amp, 1q/7 gain, 10q (PTEN) & 16q loss	MYC amp, MYCN subclones, chr 7 gain, 10q loss (PTEN)	MYC and sometimes MYCN amplification, chr 8 gain	MYC amplification, i17q (Group 4)	MYC amp, i17q (Group 4), chr 8 amp
Stemness Markers	CD133, Nestin, SOX2	Moderate: β3-tubulin, MAP2	β3-tubulin, GFAP, NeuroD1 (with RA)	CD133, SOX2, Nestin	Not specified	CD133, Nestin, SOX2	CD133, Nestin, SOX2	Not specified
Drug Sensitivity	SMO inhibitor (vismodegib), HDAC inhibitor (panobinostat)	Partial SMO inhibitor response, ABCB1-mediated drug resistance	Vismodegib, RA modest cisplatin resistance	HDAC inhibitors (panobinostat), resistant to cisplatin/vincristine	Not specified	Resistant to cisplatin/etoposide, BET inhibitors (MYC-targeted)	Resistant to cisplatin/etoposide, drug efflux via ABCG2	Not specified
Models Strengths	CSC dynamics, SHH pathway, plasticity	Differentiation studies, SHH pathway	Differentiation therapy model	High-risk, metastatic, CSC studies	Leptomeningeal metastasis model	Metastatic behavior, therapy resistance	Metastasis formation, drug resistance	Hematogenous metastasis (lung/liver)
Limitations	Low metastasis *in vivo*, genomic drift	Low metastasis, genomic instability	Differentiation potential lost in long-term culture	Genomic drift with time	Not specified	Genomic drift	Genomic drift, moderate metastasis	Not specified

ABCB1, ATP-binding cassette sub-family B member (P-glycoprotein); BET, bromodomain and extra-terminal domain; DN, desmoplastic/nodular; GFAP, glial fibrillary acid protein; HDAC, histone deacetylase inhibitor; MAP2, microtubule -associated protein 2; PTCH1, protein patched homolog 1; PTEN, phosphatase and tensin homolog; SOX2, sex determining region Y-box2; SMO, smoothened; RA, retinoic acid; SHH, sonic hedgehog protein; CSC, cancer stem cells; The features of the cell lines are described in detail in the site https://www.cellosaurus.org/ and the references from 71, 76–105, and reviewed in ref. 78.

Several others cell lines have been selected and described in the literature reviewed partly elsewhere (78, and listed in https://www.cellosaurus.org/search?query=medulloblastoma+cell+line). WNT and group 4 MB subtype cell lines are underrepresented compared to the frequency of these MB (78). For instance, the CHLA-01-MED and CHLA-01R-MED have been used to investigate features of primary and metastatic cells of group-4 MB (79) or the MED6 and MED5R of WNT group and MED1 of group 4 to evaluate the function of the MDR1 (ABCB1 molecule) to study drug-resistance mechanisms (80).

The most used and represented in literature is the DAOY cell line. This cell line has been derived from a desmoplastic tumor in a 4-year-old male and categorized within the SHH subgroup (81). It harbors a heterozygous *PTCH1* mutation (c.1312G>T; p.G438X), leading to constitutive SMO activation (54, 55, 76, 77). Genomically, it exhibits 9q loss (*PTCH1* locus) and 10q gain (*MYCN* locus), consistent with SHH-MB. DAOY retains wild-type *TP53*, differentiating it from TP53-mutant SHH MB. These adherent cells form neurospheres under serum-free conditions, expressing CSC markers such as CD133, nestin, and SOX2 (77). DAOY xenografts show desmoplastic histology and it expresses SHH targets (GLI1, *MYCN*) responding to SMO inhibitors like vismodegib (82). However, resistance via *SUFU* mutations or *G88LI2* amplification can emerge (70, 82, 83). DAOY also displays HDAC1 overexpression, and HDAC inhibitors reduce cell viability (84). Limitations include non-metastatic behavior in xenografts and genomic drift over time (78, 85).The D283-Med was established from a metastatic tumor obtained postmortem from pediatric patient while the D341-Med were from the primitive medulloblastoma tumor at craniectomy (71, 86). These cells present MYC amplification and aggressive behavior (71, 78). Due to their non-adherent, suspension-like growth, these models are widely used to study metastasis and therapy resistance. Both exhibit *MYC* proto-oncogene amplification, a hallmark of Group 3 MB, correlating with their aggressive phenotype and poor prognosis (71, 87).

D283-Med presents isochromosome 17q (i17q), characteristic of Group 4, while D341-Med often has chromosome 8 gains. Their morphology mimics anchorage-independent growth, facilitating metastasis studies (71, 86). D341-Med xenografts show leptomeningeal spread; D283-Med is less metastatic but grows aggressively. Both express CSC markers (CD133, SOX2, nestin) and show intrinsic resistance to cisplatin and etoposide due to upregulated ABC transporters (e.g., ABCG2) and anti-apoptotic proteins (e.g., BCL2) (78, 86, 88–91). Notably, D341-Med responds to BET inhibitors that suppress MYC transcription (91). However, long-term culture can lead to genomic divergence, necessitating periodic validation. Nevertheless, these cell lines present some indicators of genomic drifts upon prolonged *in vitro* culture time, which can lead to unwanted clonal selection and genomic divergence from the initial primary tumor patients. This important fact raises the need to perform periodic molecular validations.

The UW228 cell line was derived from a MB recurrence (92). Unlike D283-Med and D341-Med, UW228 cells display moderate differentiation and exhibit a more adherent, epithelial-like morphology with moderate differentiation (78, 92). It carries inactivating *PTCH1* mutations and wild-type *TP53*. Genomic alterations include 9q loss, aligning it with SHH-MB. UW228 expresses intermediate neuronal markers (β3-tubulin, MAP2), distinguishing it from less differentiated models. Cultured in monolayers, it supports studies on differentiation and SHH signaling (93, 94). UW228 shows partial sensitivity to SMO inhibitors and transient suppression of GLI1, with resistance mechanisms involving *SUFU* downregulation or *GLI2* amplification. Resistance to cisplatin and etoposide correlates with elevated ABCB1 expression. It is useful for modeling therapy-naïve SHH MB and differentiation therapy, though limited by low metastatic potential and genomic instability in long-term cultures. This cell line displays a partial sensitivity to SMO inhibitors, with reduced GLI1 expression and transient growth suppression. Nevertheless, some resistance arises via *SUFU* downregulation of *GLI2* amplification (78, 95, 96). Studies have shown that this cell line also shows medium resistance to cisplatin and etoposide. UW228 cell line presents low metastatic potential *in vivo*, which limits its utility for invasion and tumor dissemination studies. Like other cell lines, it presents some genomic instability over *in vitro* passages, which fosters low-passage genetic validation.

The ONS-76 cell line was established in 1989 from a 2-year-old Japanese patient with nodular/desmoplastic MB (98). ONS-76 cells display neuronal differentiation when exposed to retinoic acid, making this cell line a suitable model for studying differentiation therapy and SHH inhibitors (98). Integrated in the SHH molecular subgroup, ONS-76 has *PTCH1* mutations or deletions, disrupting its inhibitory function on SMO and promoting GLI-mediated transcription (98). These cells retain wild-type *TP53* and harbor *PTCH1* mutations, supporting SHH pathway activation. ONS-76 expresses neuronal markers (β3-tubulin, GFAP) and undergoes differentiation upon retinoic acid exposure, marked by neurite outgrowth and NeuroD1 upregulation (98, 99). It grows as a 2D monolayer and can form desmoplastic xenografts. ONS-76 is sensitive to SMO inhibitors, and unlike DAOY, rarely develops GLI2-driven resistance, likely due to functional *TP53*. Retinoic acid reduces proliferation via HDAC inhibition (100). This cell line also shows modest cisplatin resistance. ONS-76 is ideal for SHH-targeted differentiation therapies, though differentiation capacity may decline with extended culturing.

The HD-MB03 cell line, established in 2014 from a MYC-amplified, represents a critical preclinical model for studying high-risk, metastatic MB biology (101). It exhibits *MYC* amplification, 1q/7 gains, and loss of 10q (*PTEN*) and 16q. These alterations enhance proliferation and apoptotic resistance (56, 57). HD-MB03 forms floating spheroids and shows robust CSC marker expression (CD133, SOX2, nestin) (102). *In vivo*, it replicates leptomeningeal and spinal metastases. The cell line is intrinsically resistant to cisplatin and vincristine due to high ABC transporter and BCL2 expression. HDAC inhibitors such as panobinostat disrupt MYC-driven transcription and reduce tumor growth (91, 103). HD-MB03 serves as a strong platform for testing MYC and epigenetic-targeted therapies, though periodic molecular verification is necessary due to potential genomic evolution.

D425-Med and D458-Med were established in the late 1990s from high-risk, recurrent cases, both showing *MYC* amplification (104). Both have high proliferative and metastatic capacities. D425-Med shows 7q gain and 10q loss (*PTEN*), driving PI3K/AKT pathway activation, while D458-Med presents i17q and chromosome 8 amplification. Both grow as suspension spheroids and demonstrate strong *in vivo* metastatic capabilities. D425-Med shows leptomeningeal dissemination in orthotopic models; D458-Med metastasizes to lung and liver via systemic injection (104, 105). D425-Med may harbor *MYCN* amplification in subclones, enhancing proliferation. These models are vital for studying hematogenous dissemination and high-risk MB mechanisms.

## 3 Conventional and advanced models for studying MB biological properties

It is becoming evident that some features of tumor cells are better resembled *in vitro* using three-dimensional (3D) cultures instead of conventional cell cultures in flat-bottomed plates with adherent cells to plastic or matrix substrate (106–111). 3D cultures such as organoids and spheroids together with orthotopic animal models of MB can be useful to study the mechanisms of resistance to therapy as well as the interaction with other components of the Tumor microenvironment and immune system (**Figure 1**). The next chapters will deal with these up-to-date unconventional cultures and animal models in MB.

### 3.1 *In vitro* models: from conventional to 3D spheroid and organoid models

Like in other human tumors, traditional cultures of cell monolayer remain widely used in research, providing a controlled environment for rapid drug screening and genetic manipulation. Still, *in vitro* culture conditions can influence both phenotype and cell signaling and drug sensitivity to a large extent. Simplistic conventional, also inappropriately called 2D, monolayer cultures with a single layer of cells attached to treated polystyrene have unnaturally low cell densities, lack cell-cell interaction or cell-peri and extracellular matrix interactions, and lack the exhibition of the brain tumor nutrient gradients or physiological levels of oxygen (112). For instance, it has been shown that some MB cell lines including DAOY (SHH), ONS-76 (SHH), D458 (Group-3), HD-MB03 (Group-3), CHLA-01-MED (Group-4) and CHLA-01R-MED (Group-4) showed the growth and metastatization features of MB tumors of each subgroup they belong only when they were cultured in a 3D hyaluronic acid hydrogels but not when cultured in conventional conditions as adherent cells (113). This would suggest that 3D cultures may be used to study MB behavior and drug sensitivity, mimicking better the *in vivo* physical conditions (113).

Ivanov et al. highlighted the importance of using different 3D culture systems with the relevant tissue architecture and phenotype as well as normal tissues and how the establishment of a collaborative online database linked to distinct cell banks would catalyze preclinical MB research (78, 112–114). The use of 3D spheroid models from cell lines and patient-derived xenografts (PDX) demonstrates having more representative resistance to conventional chemotherapies, e.g. etoposide and cisplatin, in comparison to conventional cultures, and the same 3D experiments were key to identifying hypoxia-induced genes that drive resistance. Brabetz and his co-authors generated PDX-derived 3D organoids retaining genetic and transcriptional heterogeneity of primary tumors that were strategic to demonstrate that group 3 organoids with *MYC* amplification exhibit invasive growth patterns depending on the 3D extracellular matrix (ECM) (such as collagen) and that venetoclax (a *BCL-2* inhibitor) synergizes with chemotherapy, overcoming apoptosis resistance (115, 116). The use of 3D culture assays has become central in research due to their ability to recapitulate key aspects of tumor biology, such as CSC niche enrichment, intra-tumoral heterogeneity preservation, and therapy resistance modeling. Using 3D models, Vinci et al. have identified and demonstrated *MYC*-dependent metabolic vulnerabilities in Group 3, including sensitivity to glutaminase inhibitors (117). In the past decade, many 3D bio-printed brain tumor models were developed for glioblastoma aiming for the recapitulation of TME and developing better drug screening platforms (118, 119). Still, to the best of our knowledge, there are no studies that developed 3D bio-printed constructs for other brain tumors such as MB. (**Figure 1**).

### 3.2 *In vivo* models

Similar to other brain malignancies, the study of MB frequently relies on established cell lines, from murine and/or human origin, and PDX models. Models have provided critical insights into tumor biology and therapeutic response. The heterogeneity of MB has been addressed *in vivo* through the development of genetically engineered mouse models (GEMMs), through the orthotopic implantation of murine cerebellar progenitor cells and by PDX murine models. This section aims to provide a concise and short overview of the MBs *in vivo* models employed in research. It is not intended to serve as a comprehensive account of all available models, but rather to offer a general perspective on the principal systems currently utilized in the field. For more exhaustive information on *in vivo* models applied for MB research, readers are referred to the detailed reviews (120, 121).

GEMMS involve targeted modifications of the murine genome, such as gene knockout (deletion), knock-in (mutagenesis), or transgenic overexpression, enabling the study of tumor biology within an intact immune system and native tissue microenvironment. Unlike transplantation-based systems, GEMMs recapitulate spontaneous tumorigenesis, providing insights into the multistep progression from initiation to malignancy (120, 121). In research, GEMMs have been pivotal in confirming genetic drivers and elucidating the cellular origins of molecular subgroups, particularly SHH and WNT. Despite their high cost, technical complexity, and time-intensive nature, GEMMs remain among the most informative and widely utilized systems in cancer biology (120, 121). This model has been instrumental in validating oncogenic drivers and elucidating the cellular origins of distinct molecular subgroups, particularly SHH and WNT MB. For example, key studies on SHH MB utilized PTCH1 heterozygous mice (PTCH1^+/−^), which develop tumors at low penetrance (~20%) following loss of the wild-type PTCH1 allele (122). Subsequent conditional or global knockout models have identified cooperative oncogenic alterations and solidified the granule cell progenitor (GCP) lineage as the cell of origin, with GCP identity being essential for SHH-driven tumorigenesis (123).

In contrast, WNT MB is proposed to arise from dorsal brainstem progenitors derived from the lower rhombic lip, rather than cerebellar compartments. Conditional knock-in models expressing a constitutively active β-catenin variant demonstrated that aberrant WNT pathway activation induces pathological cell accumulation in the brainstem but not the cerebellum, which can be aligned with putative extra-cerebellar origins (124). Additionally, transgenic mouse models overexpressing *NMYC* in cerebellar tissue have further revealed oncogenic versatility by inducing resembling MB in multiple subgroups, such as groups 3, 4, and SHH. This fact highlights the fact that the context-dependent effects of oncogene activation on pathogenesis.

PDX models have gained prominence in oncology research due to their capacity to closely mirror the biological and histopathological characteristics of the primary tumors from which they originate. In the context of MB, PDXs are typically generated by engrafting freshly resected tumor tissue either subcutaneously or within the cerebellar parenchyma-orthotopic engraftment into immunodeficient mice (125). The genetic diversity of recipient mice—covering inbred, outbred, or hybrid strains—does not preclude successful xenotransplantation. Nevertheless, the fact that these animals are immunosuppressed, a condition needed to allow tumor engraftment excluding the rejection in adult mice, represents a restraint in their use as immunotherapies testing (126). PDX models have been successfully established from all major molecular MB subgroups and have demonstrated stability across multiple passages, although subclonal populations may undergo selection during serial transplantation. We highlight that most of the PDX models have been derived from high-risk cases, suggesting that tumors with more aggressive phenotypes are more amenable to engraft. Also, orthotopic implantation into the cerebellum has been associated with improved engraftment efficiency, particularly for tumors with lower proliferative potential, compared to subcutaneous approaches (127, 128). These types of *in vivo* models have been instrumental in validating key molecular drivers of pathogenesis and serve as versatile platforms for evaluating a range of therapeutic strategies for preclinical assessment of pharmacologic agents, dosing regimens, delivery routes, and combination treatments, but not so key for cell-based and immunotherapies due to animal immunodeficient environments. (**Figure 1**).

More recently, both MB cell lines and patient-derived cells (from SHH MB, SHH PDX, the Group 3 MB PDX cell line MB-LU-181) have been transplanted into blastula stage of zebrafish embryos leading to orthotopic MB growth (129) The localization to the hindbrain region of transplanted cells was increased by culturing MB cells in neural stem cell-like medium. This model could be used to test the efficacy of SMO inhibitor sonidegib and an active metabolite of cyclophosphamide (129). The model of zebrafish has been also used for group-3 MB cells (130) to identify the subgroup MB cellular origin (131), to mimic the SHH-MB with specific mutations (132), to generate transcription activator-like effector nucleases TALEN-mediated somatic gene inactivation of CDKN2A/B or RB1 tumor suppressor genes (133). Altogether, zebrafish models can well resemble the growth and the aggressiveness of MB in humans to analyze the effects on MB biology due to the genome editing and the presence of specific mutation and/or activation of oncogenes (130–134) (**Figure 1**).

## 4 MB immune response and immunotherapy

As defined by the National Cancer Institute, “immunotherapy is a type of cancer treatment that helps your immune system fight cancer.” The immune system interacts with autologous cells, and it is educated for not reacting with self but only with something that is sensed as “foreign” (7–11). MB cells are autologous cells and, by definition, should not have been recognized by the adaptive arm of the immune system (69). Indeed, T lymphocytes should be impaired to eliminate self-cells, as self-reacting T lymphocytes have been deleted during thymic central selection (135, 136). Mistakes in the mechanisms of deletion allow the insurgence of an autoimmune disease if the peripheral tolerance does not work too (135–137). On the other hand, innate cells such as NK cells do not react with self-cells as they bear inhibitory receptors that interacting with self-major histocompatibility (MHC) class I alleles can impair killing of autologous cells. The innate arm of the immune system can kill target cells when tumor cells do not express MHC or when the activating signals are stronger than the inhibiting ones (138–142). The relevance of T lymphocyte response against MB can be exploited by two main tools: T cell-specific for associated peptide antigen or neoantigen expressed on MB cells presented by antigen-presenting cells (APC) or engineered chimeric antigen receptor (CAR) T cells recognizing surface receptors on MB cells (143). First, we will analyze the immune cells potentially involved in the response to MB namely: NK cells, lymphocytes subsets with functional properties between NK and classical αβTCR T cells and finally anti-MB specific αβTCR T cells. Second, the targeting of MB surface antigens with immunotherapeutic tools will be considered. Finally, we considered some potential molecular target expressed by the TME which can help to relieve the TME-mediated immunosuppression.

### 4.1 Innate immune response

**Figure 2:**
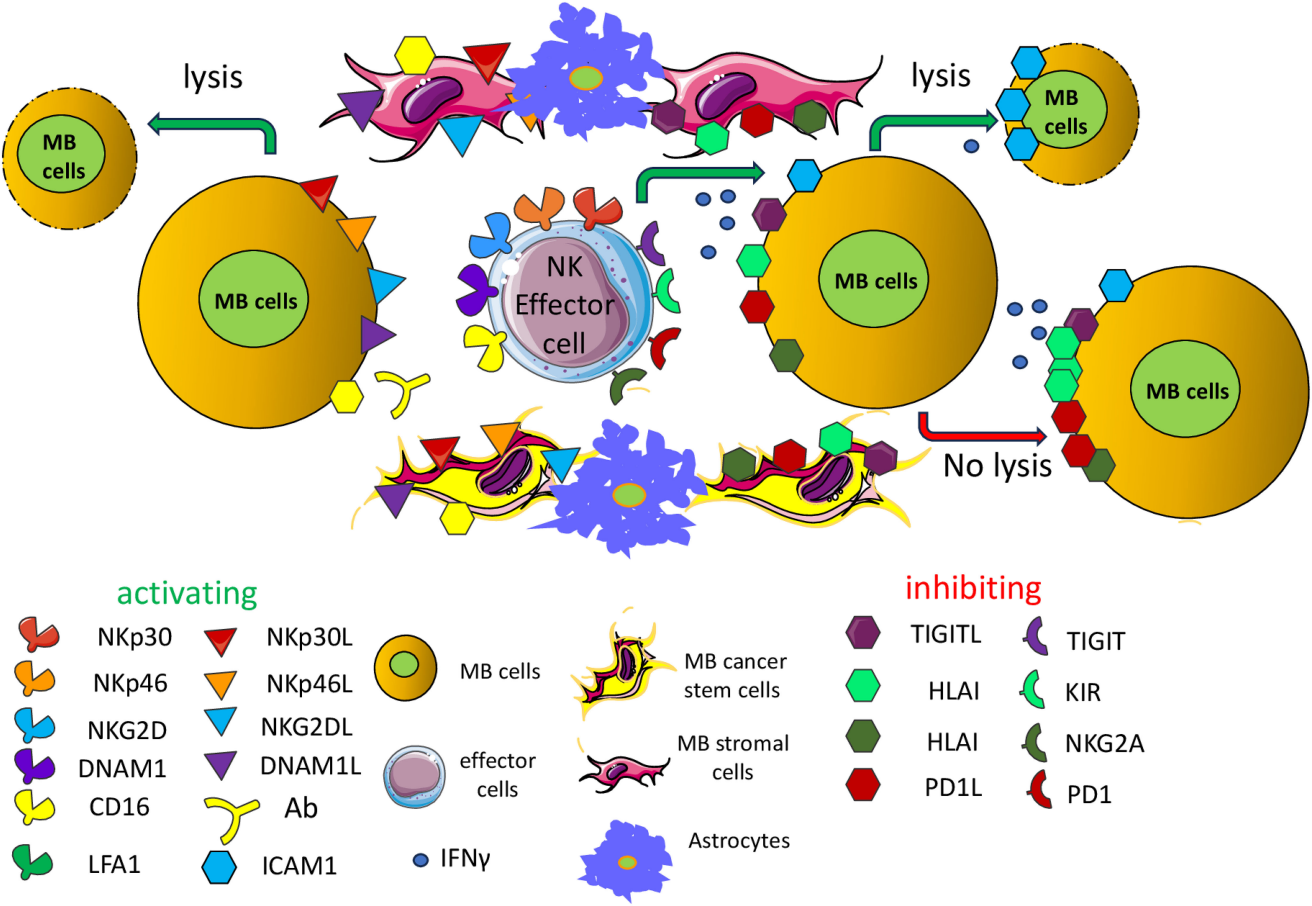
Activating and inhibiting receptors expressed on antitumor NK effector cells. Antitumor effector NK cells can express a plethora of receptors, whose engagement through the corresponding ligands expressed on MB cells can deliver an activating (green) or inhibiting signal (red). These ligands may be expressed also by other cellular components of the MB TME including stromal cells, cancer stem cells, endothelial cells and astrocytes. NKp30, NKp46, NKG2D, DNAM1 are some of the main activating receptors typically expressed on NK cells and subsets of T cells (effector cells). Some inhibiting receptors are represented by TIGIT (T cell immunoreceptor with Ig and ITIM domains), KIR (Killer Ig-like inhibitory receptor), NKG2A (killer cell lectin-like receptor subfamily C, member 1) and PD1 (programmed cell death receptor 1). The interaction of these receptors with the corresponding ligands on target cells can activate or inhibit the effector function of NK cells or subsets of T cells. The final outcome is related to the degree and/or affinity of each receptor/ligand interaction. The presence of the ligands on target cells regulates the fate of MB cells. The reported low expression of HLA-I on MB cells could limit the negative signal in self-NK cells of the killing of tumor cells, favoring the activation through the engagement of activating receptors. The production of IFNγ can lead to upregulation of HLA-I, PDL1 and ICAM1 exerting opposing effects on NK cell-mediated killing. The TIGIT (inhibitory) and the DNAM1 (activating) receptors recognize the same surface ligands, CD115/PVR and/or CD112/nectin2. The expression on effector cells of the CD16/FCγRIIIa can trigger cytolysis of target cells (antibody dependent cellular cytotoxicity, ADCC) in the presence of an antibody that links the CD16 on effector cells through its FC portion and makes a bridge with target cells by binding the antigen through its Fab component. CD16 is one of the main activating receptors of NK cells. Typically, the ADCC is mediated by antibodies of the γ1 (IGG1) isotype but not by the γ4 isotype (see **Table 3**). In this figure, activating and inhibiting ligands of MB cells are artificially shown on separate target cells, but this is an oversimplification. All these ligands may be present on the same MB cells. It is to note that the expression of the ligands for the various activating receptors is determined either by the reported expression on MB cells using 1- specific anti-ligand antibody; 2- with covering of the activating receptor with anti-receptor antibody leading to a reduction of cytolysis of target cells. Only the effects on MB cells are depicted but similar effects may be exerted on the other component of the TME such as stromal cells, astrocytes and MB cancer stem cells.

It has been reported that NK cells can recognize and kill the MB cells (**Figure 2**) (160–164). The established cell lines DAOY and D283-Med and the primary cell line 1603-Med from an anaplastic MB can express different levels of ligands for the NK cell activating receptor NKG2D including MICA/B and ULBP3; further, they express nectin-2 and PVR, ligands for the DNAM1 activating receptor, low levels of LFA3/CD58 (a ligand of CD2 antigen express by most lymphocytes) and intercellular adhesion molecule (ICAM) 1, 2 and 3 ligands for the lymphocyte function associated antigen (LFA) 1. More relevantly, the use of specific mAb against NK cell activating receptors such as NKG2D, DNAM1, NKp30 and NKp46 could inhibit the NK cell-mediated cytolysis of these cell lines (161). The expression of ligands for NKG2D has been further confirmed both in immunohistochemistry of MB specimens and in cell lines, together with the relevance of NKG2D and HLA-class I molecules in NK cell-mediated recognition of the MB cell line DAOY (162). This finding indicates that several counter-ligands of NK cell-triggering molecules may be considered suitable targets for the elimination of MB cells in patients (162). The adoptive transfer of IL15-activated NK cells can induce a delay in the growth of the MB DAOY cell line in a subcutaneous xenograft mouse model (160), leading to an increase in the OS rate. The infiltration of these xenografts was characterized by NK cells showing expression of several activating receptors and bearing several markers of cytotoxic cells, including perforin, granzyme, tumor necrosis factor (TNF)α, and interferon (IFN)γ. It is well known that NK cells can recognize tumor cell targets independently by the recognition of HLA-I differently from T lymphocytes (141, 142). In this context, it is essential to be better able to define the role of HLA-I expressed on MB cells, as typically NK cells express potent inhibitory receptors for self-HLA-I, leading to blocking of autologous cell killing (141, 142). It has been reported in a series of 10 MB primary tumors the absence of reactivity with anti-HLA-class I antibodies in immunohistochemistry assays (165). This finding has been confirmed on a large series (n=106) of MB, but in addition, it has been shown that high levels of HLA-I are strongly expressed only in MB with evident anaplastic features in association with MYC expression (166). A role of HLA-I peptide complexes and the endoplasmic reticulum aminopeptidase (ERAP)1 in the regulation of MB cell killing has been reported (167). However, the increase of cell killing upon blocking of HLA-I recognition by NK inhibitory receptors was faint (161, 167). Furthermore, the degree of killing of the polyclonal NK cell populations against the same target (DAOY cell line) used in these reports is markedly different (161, 167). This variability would indicate a variable expression of the activating receptors expressed on NK cells. Thus, the killing of MB cells is the result among positive and negative signals transduced by activating or inhibiting receptors upon binding with the corresponding ligands on MB cells (**Figure 2**). It is of note that an evident cytotoxic effect was also detected by injecting ^19^F-labeled NK cells intratumorally or contralaterally to an orthotopic MB tumor in immunodeficient mice (168). Further, it is of note that NK cells are more infiltrated in patients with a better prognosis (169). Altogether, these findings further support the possibility of using adoptive NK cell transfer as a useful tool to eliminate MB cells and control tumor expansion.

### 4.2 Effector lymphocyte with phenotypic and functional properties between NK and T cells and recognition of MB cells

**Figure 3:**
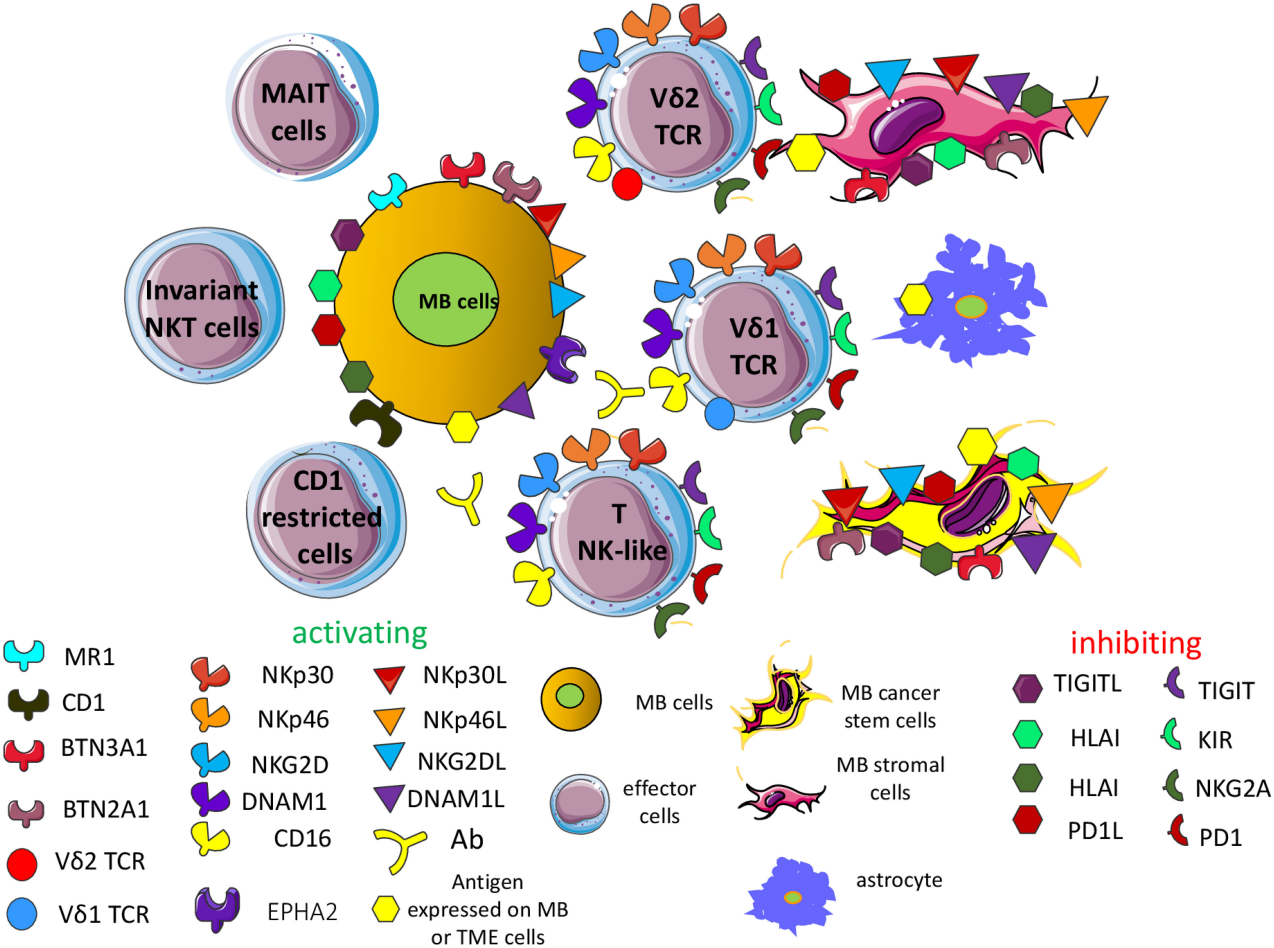
Additional effector cells targeting the MB cells. Several effector cell subsets can recognize the MB cells on the basis of the expression of some of the activating receptors expressed typically by NK cells. These subsets comprise invariant NK-like T cells, Vδ2 and Vδ1 (γδ TCR) T cells, CD1-restricted T cells, mucosal associated invariant T cells (MAIT) and other NK-like T cells. Typically, these cell subsets can show functional features of NK cells, such as killing of tumor target cells independently of the recognition of HLA-I antigens. Some of them express activating and inhibitory receptors like NK cells, and the final outcome of their engagement is similar to what is observed in NK cells. In detail, the Vδ2T cells can trigger TCR-mediated activation through the recognition of small phosphate antigens (pAg, such as isopentenyl pyrophosphate, IPP) presented by the butyrophilin members, including BTN3A1 and BTN2A1. pAg are derived from intermediates of the cholesterol synthesis of mevalonate pathway. Aminobisphosphonate such as zoledronic acid can increase the presentation of small pAg on tumor MB cells while Vδ1T cells can recognize the EPHA2 leading to the killing of MB. MAIT cells can recognize the major histocompatibility complex class I-related gene protein (MR1) presenting intermediates of riboflavin synthesis. On the other hand, CD1-restricted T cells can recognize lipid antigens instead of peptide antigens, like the majority of αβ TCR T cells. Lipid antigens can derive from endogenous or foreign origins. Overall, these cell subsets can recognize tumor cells if they express the corresponding counter-ligands (see also the **Supplementary Figures 1, 2**).

The relevance of some unconventional T cells, such as invariant NK-like T cells, Vδ2 (γδ TCR) T cells, CD1-restricted T cells, mucosal associated invariant T cells (MAIT) and other NK-like T cells, has not been studied in detail with MB cell lines and MB preclinical mouse models (65, 170–175). It has been reported that MB do not express CD1d, besides HLA-I, suggesting a molecular mechanism involved in MB escape from recognition by unconventional T cells (173). Furthermore, the molecular MB SHH subgroup can express elevated levels of CD1d mRNA compared to other MB subgroups (173). Importantly, CD1d-positive MB cells (DAOY and MED8A cell lines) presented glycolipid antigens (α-galactosylceramide) to NK T cells, inducing the production of cytokines such as IFNγ and IL4. Along this line, NKT cells induced remission of orthotopic injected MB xenografts of DAOY MB cells. In addition, the NKT cells in MB patients were present and functional, suggesting the possibility of using these NKT cells to kill MB cells at least in a subset of patients (173). More recently, it has been reported that γδT cells can infiltrate the MB, and in particular the group 4 MB, but there was not a significant correlation between the infiltration of γδT cells and patients OS. Furthermore, EphA2-expressing MB cells trigger Vγ9Vδ2T cell activation while amino bisphosphonates sensitize MB cells but not healthy neuronal cells to Vγ9Vδ2T cell lysis (175). This would indicate that γδT cells can be a useful tool to target MB cells. In this context, we reported that antibodies to tumor antigens conjugated with the aminobisphosphonate zoledronic acid can kill efficiently tumor and stromal cells (176). Thus, we can hypothesize that the use of antibodies to MB antigens conjugated with aminobisphosphonates can be effective in eliminating MB cells, as shown for colorectal carcinoma (176). Finally, it has been reported that six MB cell lines (DAOY, ONS-76, UW228, D341, D425 and D283) can be a good target for protein tyrosine kinase (PTK)7-targeted CAR γδ T cells against MB (177). Altogether, these findings support the idea that besides conventional T cells and NK cells, other effector lymphocytes can be an immunotherapeutic tool to eliminate MB cells (**Figure 3**).

### 4.3 Tumor antigens of MB

**Figure 4:**
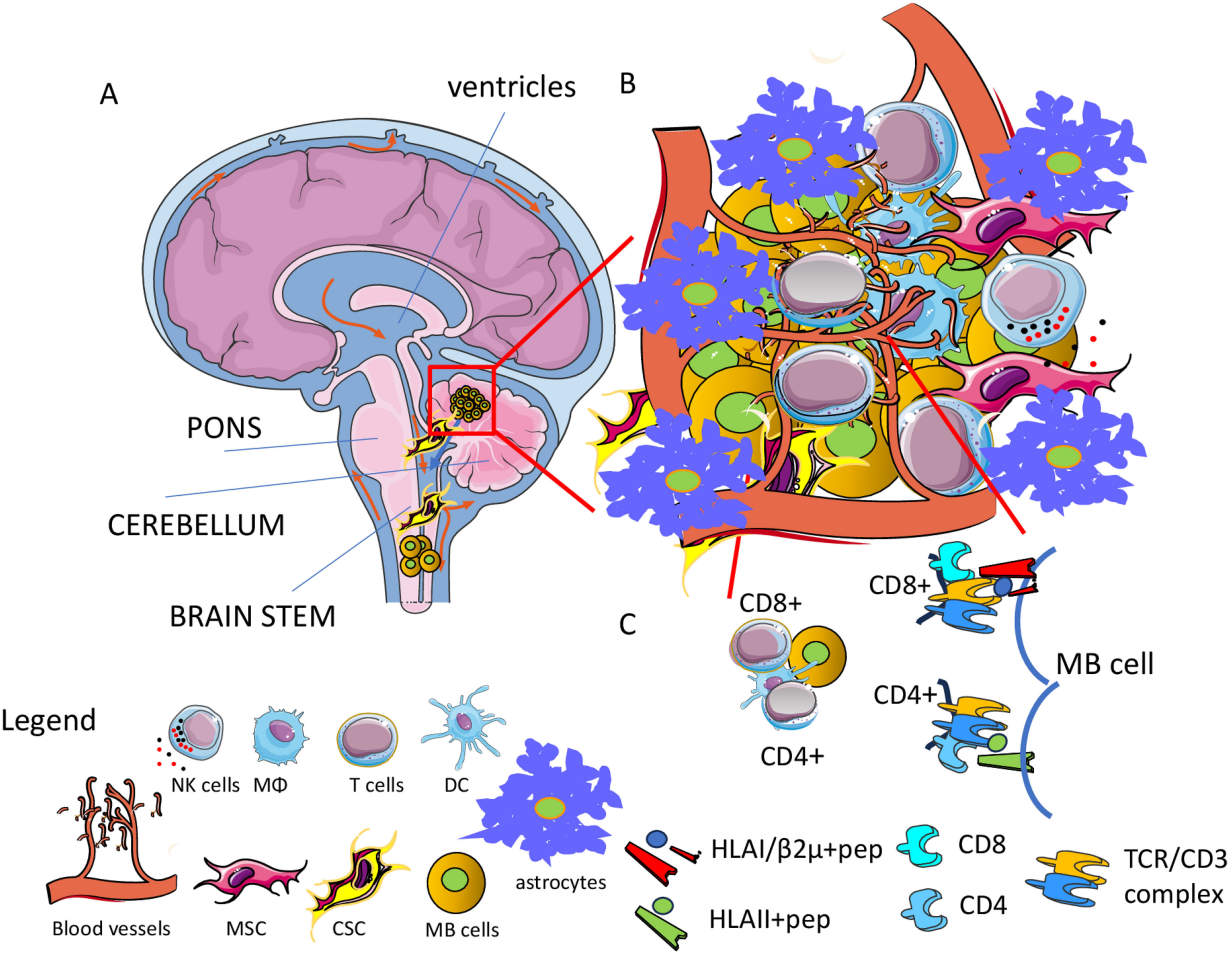
Tumor microenvironment (TME) in MB. **(A)** MB is a tumor usually localized in the cerebellum leading to metastasis in the spinal cord. **(B)** The TME is characterized by the presence of several cells of the immune system as macrophages (MΦ), dendritic cells (DC), and T cells together with mesenchymal stromal cells (MSC) and astrocytes (As). MB cancer stem cells (MB CSC) can be considered a major target of therapy to eradicate the tumor. Typically, the TME leads to the impairment of the immune response against tumor cells. Angiogenesis is an essential process that favors the growth of this tumor and possible spreading to other regions of the brain. The brain-blood barrier is a key anatomical and functional structure involved in the regulation of infiltration of antitumor effector cells, as well as tumor spreading and drug effectiveness. **(C)** Adaptive specific T cell immune response can be elicited against the MB cells and MB cell antigens potentially presented to either CD4^+^ or CD8^+^ T cells can be identified mainly by OMICS analysis.

The antigen-specific immune response could be considered one of the cellular-mediated mechanisms by which MB cells can be eliminated. Briefly, an adaptive immune response should be elicited against neoantigens and/or tumor-associated antigens (TAA) (178). The presence of neoantigens and TAA is also necessary for awaking exhausted tumor-infiltrating lymphocytes (TIL) upon treatment with immune checkpoint inhibitors (ICI) such as anti-CTLA4 or anti-PD1/PDL1 antibodies (179–183). Importantly, the possibility of evoking an adaptive immune response is one of the requisites to plan ICI therapy, and the identification of neoantigens or TAA together with antigen-specific TIL justify the cost of the treatment and predict, at least in principle, the response (183). Conceivably, more are the neoantigens and TAA present together with their immunogenic potency; more probable is that the immune system will control the tumor growth. As reported above, the patients can be subdivided on the basis of subgroup-specific genetic alterations, and more recently it has been shown that it is possible to identify potential tumor antigens, possibly suggesting the development of antigen-directed cellular therapies for MB (184). In detail, it has been developed an algorithm predicting antigens able to trigger an immune response in the context of the patients’ HLA-class I and class II. This point is relevant as it is well-known that the immune response needs both the help mediated by the recognition of CD4+ T cells of MHC-II-restricted peptides and the lytic activity of CD8+ T cells restricted to MHC-I antigens to eliminate tumor cells (185) (**Figure 4**). The antigens considered include neoantigens, TAA and fusion antigens (184). This pipeline has been named Open Reading frame Antigen aNalysis (ORAN) and it uses the gene expression data for identifying different classes of antigens (184). Importantly, the algorithm can predict putative peptide antigens that indeed trigger an efficient antitumor immune response after vaccination in preclinical glioblastoma models (186). It is of note that ORAN identified that only a subset of the genes mutated in MB could be immunogenic, and this subset was present in about 80% of patients; also, 44% of patients expressed three or more neoantigens. This would indicate that not all the MB patients may benefit from neoantigen-based immunotherapy. About 90% of MB expressed at least one TAA, and a quite high proportion of these patients expressed three TAA. Noteworthy, the TAA prediction showed a strong and better concordance with respect to neoantigens with proteomic data. The overall survival (OS) and the progression-free survival (PFS) of patients in the Group 3 of MB well correlate with the presence of MHC-I and MHC-II peptides of TAA. This work analyzed by RNA-seq 170 cases of MB of which 18 WNT, 46 SHH, 41 Group 3, and 65 Group 4, and it used a data set of microarray technology of 763 MB (64, 184). Moreover, the immune landscape and the pathways for antigen processing and presentation in tumor cells have been studied using up-to-date deconvolution computational methods. This analysis has given some insights on the possibility of identifying the so-called “recurring antigen”. It is conceivable that to design an MB vaccine potentially functional in any patient independently of the molecular subgroup and stage of development, a good immunogenic antigen should be identified. It is of note that MB expressed several private and immunogenic antigens. Except for the SHH-MB subgroup, several TAA were usually expressed. Furthermore, some cancer testis and neurodevelopmental antigens were expressed throughout all the subgroups. In detail, neoantigens from oncogenic driver mutations including *CTNNB1*, *DDX3X*, and *SMARCA4* and TAA such as *NEUROG1* and *PIK3R3* could be considered as potential therapeutic targets for immunotherapy. None of these identified potential targets have been validated in experiments showing that it is possible to trigger an immune response upon the use of a personalized vaccine. However, the same ORAN pipeline applied to the murine glioma GL261 cell line identified, after appropriate selection, 192 putative neoantigens and 37 TAA with a predictive immunogenic effect (186). Importantly, it has been developed a gene enrichment platform for the production of tumor open reading frames that are unique (TOFU) specific for tumor antigens. This platform allowed the generation of mRNA by *in vitro* transcription for immunogenic tumor antigens without the need of providing large tissue samples from patients to obtain these mRNAs, overcoming this bottleneck to produce vaccines. Noteworthy, the TOFU mRNA vaccine was efficient to evoke an antitumor response in a murine model of glioma when loaded into dendritic cells in combination with immune-checkpoint inhibitors (ICI) and/or adoptive cell therapies (72, 187).

It is clear that these novel approaches in identifying the MB neoantigens and TAA support the idea that immunotherapy might be feasible and efficient. Previous observations in MB on the immunogenic property of the fusion proteins composed by the enhancer of polycomb homolog 2 (EPC2) and GULP PTB domain containing engulfment adaptor 1 (GULP1) have shown that this protein can trigger the release of IFNγ by CD8^+^ T cells (188). Also, the finding that a CD8-specific T-cell response can be elicited in neoepitopes derived from Histidine Ammonia-Lyase (HAL), Neuraminidase 2 (NEU2), Proprotein Convertase Subtilisin (PCSK9), Programmed Cell Death 10 (PDCD10), Supervillin (SVIL) and tRNA Splicing Endonuclease Subunit 54 (TSEN54) variants is in line with the notion that specific T cell-mediated immunotherapy can be applied for MB (189). Finally, the proteogenomic approach allowed the identification of neoantigens from MB tumors with a low mutational burden and a limited amount of tissue (189). It is of note that T cells with different T cell antigen receptors (TCR) and producing several antitumor cytokines can be obtained using these neoantigens. This finding further supports the more recent publications (184, 186, 189) that it is possible to discover immunogenic-specific peptides that are the basis for generating appropriate anti-MB vaccines.

### 4.4 Targeting molecules expressed by MB: antibodies and CAR cells

**Figure 5:**
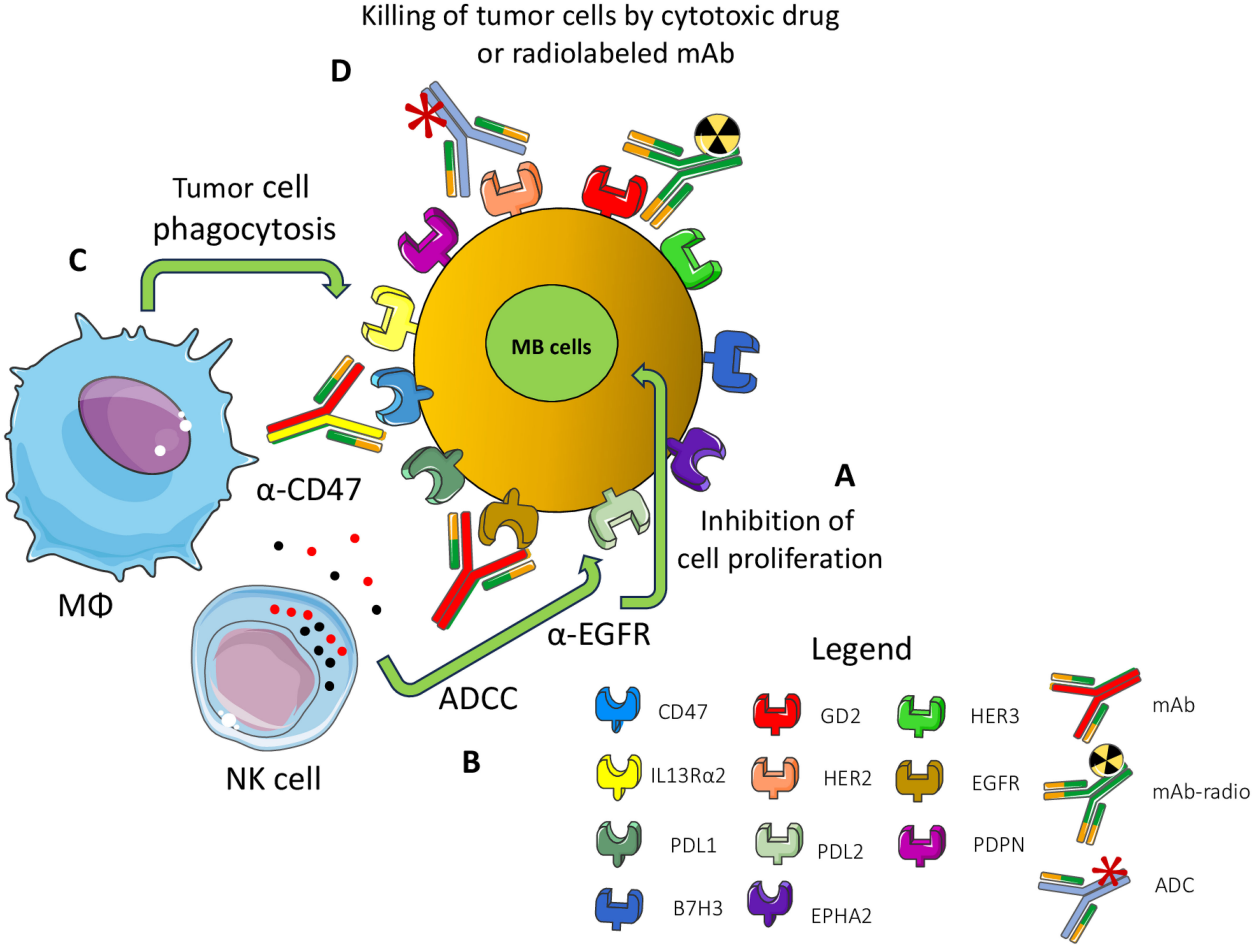
Surface MB receptors as targets for immunotherapy with antibodies. Several surface-expressed receptors of MB cells can be a target for therapy with monoclonal antibodies (mAb), radiolabeled-mAb (radio-mAb) or antibody drug conjugates with cytotoxic molecules (ADC). Some representative molecules are shown. EGFR, HER2 and HER3 are members of the epidermal growth factor receptor family: PDPN: podoplanin, PDL1/2: ligands for PD1, GD2: disialoganglioside, EPHA2 ephrin receptor A2, IL3 receptor alpha2. Not necessarily all these molecules are expressed at the cell surface of the same MB tumor cell. The antibodies directed against these molecules can inhibit **(A)** the proliferation of MB cells by blocking the binding with the natural ligand as growth factors (e.g., anti-EGFR Ab). **(B)** The Ab can trigger antibody-dependent cellular cytotoxicity (ADCC) by lymphocytes (such as anti-EGFR mAb), monocyte/macrophages and other innate cells or complement dependent cytotoxicity. **(C)** Antibodies can block the “do not eat me” signal (e.g., CD47) leading to phagocytosis or **(D)** they deliver cytotoxic drugs or radio-isotopes leading to the killing of tumor cells.

**Figure 6:**
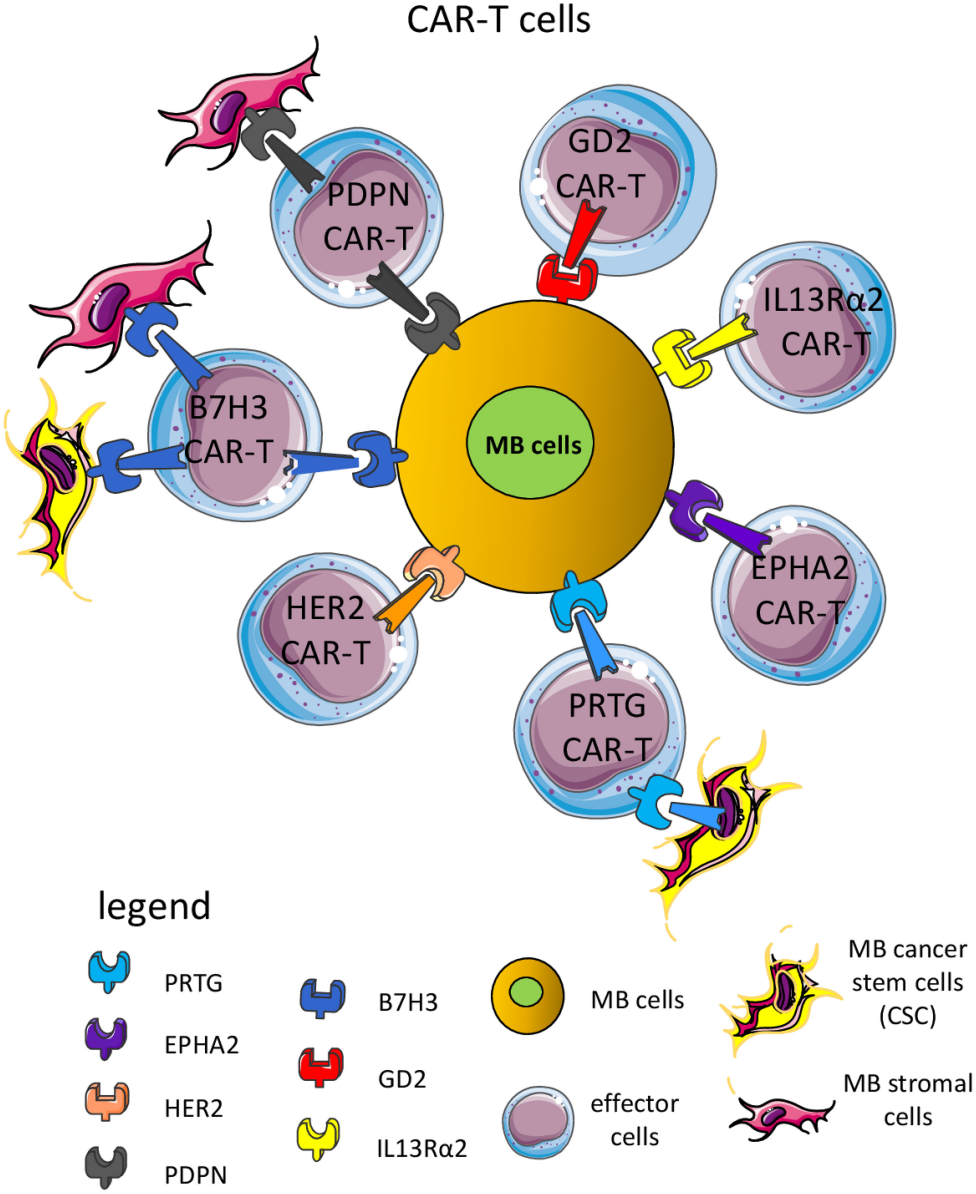
CAR-T cells are a suitable means to kill MB cells and other cellular components of the TME. Several CAR-T cells have been used in preclinical murine models and some clinical trials to target surface molecules expressed by MB cells, including B7H3, GD2 (disialoganglioside), EPHA2 (ephrin A2 receptor), HER2 (human epidermal growth receptor 2), PRTG (protogenin), PDPN (podoplanin) and IL13Rα2 (interleukin 13 receptor α2). Importantly, some CAR-T cells can recognize not only MB cells but also components of the TME, such as endothelial cells, stromal cells, and/or cancer stem cells (CSC). The majority of CAR-T cells can express CD4 or CD8 antigens. Usually, CD8^+^ CAR-T cells can recognize MB cells expressing the antigen recognized by the scFv portion leading to the signal transduction through the intracellular component of the CAR-T molecule. This elicited the release of perforin and granzyme and consequent killing of the MB cell. CAR-T can produce proinflammatory cytokines (IFNγ and TNFα) with potent antitumor effects.

Beside targeting tumor-specific antigens, immunotherapy can target receptors present on MB cells but widely expressed on other cell types (144, 190–207). The first point to consider for an efficient therapeutic effect without too many side effects is the expression of appropriate target molecules at the cell surface of MB cells (196–198). The ideal target should be expressed mainly, if not exclusively, on MB cells but not on healthy cells. The second critical point is the degree of the immune response; it should be enough strong to control and eliminate tumor cells, but sufficiently milder not to damage large amounts of healthy cells. This second point is much more important, more relevant: it is the function of healthy cells as a potential target of therapy. Proved target receptors on MB cells include HER2, B7H3 (CD276), Epha2 and GD2 (144, 196, 197, 199–203). These antigens can be targeted mainly by two therapeutic tools: monoclonal antibodies (mAb) (**Figure 5**) and CAR cells (**Figure 6**).

Antibodies represent a key therapeutic tool for several kinds of cancers, including tumors of the nervous system (204, 205) and CAR cells could be considered one of the more recent tools developed from classical mAb (206–209). As for the native mAb, CAR shows a component of the engineered chimeric receptor that recognizes the target molecule at the cell surface of MB cells (202–206). The antigen recognition domain of the CAR molecule is typically derived from the variable regions of a mAb as a single-chain variable fragment (scFv) recognizing a tumor expressed antigen. The main difference consists in the molecular and cellular mechanisms of the therapeutic effects (210, 211). Antibodies can affect receptor-ligand interactions and signaling, as well as trigger complement- and/or antibody-dependent cellular cytotoxicity (CDC and ADCC) and cytokine release (212). On the other hand, CAR cells, upon the interaction of the CAR molecule, deliver an activating signal that leads to the killing of the target cells (69). Also, CAR cells produce and/or release cytokines typical for the type of cell in which the CAR has been transduced (69). CAR molecules can be transduced into classical CD4^+^ or CD8^+^αβ T cells, γδ T cells, innate cells including NK cells, monocyte/macrophages (Mo/MΦ) and *in vitro* assays show that both mAb and CAR cells are efficient tools to eliminate MB cells (213–215). The main matter with both antibodies and CAR cells is the tumor localization of the antibody and the effector cells (216, 217). This could be considered the key factor that distinguishes the strong efficacy reported for CAR cells in hematological malignancies, compared to the disappointing results found for solid tumors (217–220).

#### 4.4.1 Targeting of GD2 in MB

The GD2 is a disialoganglioside expressed during fetal development and by several tumors, but not in normal adult tissues (197). Both mAb and CAR-T cells against GD2 are enrolling patients for the treatment of different brain tumors, including MB (196, 209, 220). Recently, it has been reported in an orthotopic MB murine model that CAR-GD2.CD28.4-1BBζ (CAR.GD2)-T construct, including the suicide gene inducible caspase-9 can control tumor growth and prolong the OS of treated mice. In addition, the use of the drug AP1903 induced the dimerization of caspase-9 leading to apoptosis of peripheral blood circulating and brain tumor-infiltrating CAR-T cells. These findings indicate the possibility to efficiently eliminate tumors and limit the side effects due to antitumor effector cells. Importantly, the *in vitro* pretreatment with tazemetostat, a first-class inhibitor of activating enhancer of zeste homolog 2 (EZH2), can upregulate the GD2 expression on GD2^dim^ MB cells (196). This upregulation of GD2 was sufficient to sensitize the MB cells to CAR-GD2 T cell-mediated cytotoxicity. A clinical trial of phase I/II is ongoing (NCT05298995) to determine the safety and effectiveness of CAR-GD2 T cell therapy in high-risk patients (196).

Furthermore, the preclinical evidence of treating a genetically engineered mouse model of MB with the ultra-high dose rate radiotherapy (FLASH-radiotherapy (RT)) in association with CAR-GD2 T cells has demonstrated that the immunosuppressive MB TME can be reversed to a pro-inflammatory one giving optimal anti-MB responses (208). In detail, the FLASH radiation delivered in milliseconds can abrogate the oxidation of the lipids, reducing the activity of peroxisome proliferator-activated receptor-γ (PPARγ). This is in contrast with the generation of reactive oxygen species triggered by the standard radiation, at a lower dose but for a longer time than FLASH-RT, leading to the PPARγ activation and consequent lipid peroxidation. These events hit mainly the macrophages present in the TME, together with a reduction of arginase 1 expression. In summary, the FLASH radiation reprograms the macrophages from an immunosuppressive to a pro-inflammatory behavior favoring the CAR-GD2 T cell infiltration and MB cell killing (208). The fact that preclinical studies suggest that FLASH-RT induces fewer side effects on healthy tissues than conventional (CV) RT would suggest that FLASH-RT could modify the TME and eventually the clinical outcome of disease.

#### 4.4.2 HER2, EPHA2 and IL13 receptor α2 as therapeutic targets of MB

The systematic characterization of the tumor of the CNS identified HER2, HER3, NECTIN4, TROP2, CLDN6, CLDN18.2, and CD276/B7H3 proteins as potential therapeutic targets (**Figures 3**, **4**). Also, EPHA2 and IL13 receptor α2 are well expressed in some MB (221) and it has been reported that several cell lines as well as a consistent group of primary MB cells can express HER2 and other members of the EGFR family (156, 201, 207, 214, 215, 218, 221–233). Other molecular targets expressed by MB will be considered later in chapter 4 of this review in the context of the targeting of MB and TME. The HER2 and HER3 can be targeted by antibodies; antibody drug conjugates and CAR-T cells have been reported or present on the market (156, 229). It has been reported in different animal models that the treatment with the HER2-BBz-CAR T cells effectively clears MB tumors (227). Orthotopically implantation in the posterior fossa of NOD.Cg-Prkdc ^scid^ Il2rg ^tm1Wjl^/SzJ (NOD scid gamma deficient, NSG) mice of the DAOY or D283-Med cells led to the generation of tumors. These tumors were efficiently treated with regional or intravenous HER2-BBz-CAR T cells.

This effect was further confirmed in non-human primates (Rhesus macaques, Macaca mulatta, NHP). It is to note that the dose necessary for regional delivery was a log lower for locoregional vs. intravenous delivery. Also, no systemic toxicity was detected in NHP after intraventricular delivery of autologous HER2-BBz-CAR T cells. It is of interest that the HER2-CAR-T cells contained the CD3ζ and 4-1BB signaling motifs and showed robust anti-MB activity, indicating this kind of CAR-T cells can induce a complete and long-lasting regression of established tumors when administered regionally (227). This locoregional delivery of CAR-T cells with a medium-length CAR spacer increases the therapeutic efficacy of HER2-CAR T cells in an orthotopic xenograft model with the cell lines D283-Med and Med411FH, but not against the HER2-negative D431 cell line (234). Indeed, it appears that the cytotoxic effect of HER2-CAR-T cells with a short spacer was not evident on HER2^+^ MB cell lines. This would indicate that the length of the spacer in the case of recognition of HER2 antigen on MB cell lines is a key point to consider. Furthermore, some patients suffering from other brain tumors (ependymoma or anaplastic astrocytoma, of the BarinChild-01 NCT03500991 clinical study) did not show dose-limiting toxicity after infusion of the HER2-CAR-T cells via CNS catheter into either the tumor cavity or the ventricular system.

This suggests the feasibility of repeated dosing regimens and well-toleration of intra-CNS HER2-CAR-T cells delivery in young patients, supporting the notion that this way of administration of CAR-T cells could be adopted in MB as well. This idea is further supported by additional experimental evidence obtained using locoregional therapy with other CAR-T cell models (221). In fact, it has been validated the intrathecal delivery of a trivalent EPHA2, interleukin 13 receptor α2, besides HER2, CAR-T cell against primary, metastatic and recurrent group 3 MB xenografts in mouse models. Furthermore, these CAR-T cells alone or in coo with azacytidine is an efficient therapeutic regimen mouse model with multiple metastases of this highly aggressive MB, providing the rationale for application of the delivery of these types of CAR-T cells intracranially in humans (221). EPHA2, ephrin receptor A2, plays a key role in cancer development and its expression shows association with poor prognosis, elevated metastatic potential, and reduced survival of tumor patients (200). Also, the interleukin 13 receptor α2, the receptor for the anti-inflammatory cytokine IL13, is overexpressed in several brain tumors, playing a role in invasion and metastasis (235, 236). Overall, the three targets considered using the trivalent CAR-T cells were well expressed on the different stages of development of the MB of group 3. Conceivably, the definition of CAR-T cells to use for patient treatment should satisfy the condition of expression of the molecular targets through the primary, metastatic and recurrent MB. This condition would allow the response against the relapse of the MB.

#### 4.4.3 B7H3 inhibitory immune receptor targeting MB

The B7 homolog 3 (CD276) is a transmembrane molecule expressed in several types of cancers where it functions as an immune checkpoint receptor, and it can be targeted efficiently by both CAR-T cells and mAb (237–241). In MB, it can be involved in the angiogenesis migration, invasion (see the 4.5.1 chapter of this review), as well as MB escape from the immune system (205, 242–246). The counter receptor expressed on immune cells of B7H3 has not been identified yet, but it is conceivable that activated CD4^+^ and CD8^+^ T cells express a receptor interacting with APC cells or tumor cells through B7H3 and this interaction leads to inhibition of lymphocyte functions such as tumor cell killing and cytokine production (247–249). Four putative candidates have been suggested, including the triggering receptor expressed on myeloid cells (TREM)-like transcript 2 (TLT-2), interleukin-20 receptor subunit α (IL20RA), phospholipase A2 receptor 1 (PLA2R1), angio-associated migratory cell protein (AAMP) and possibly other molecules (249–254).

### 4.5 Targeting the MB tumor microenvironment

The features of the tumor microenvironment (TME) are relevant for the growth and diffusion of several cancers, including MB (255). The knowledge of the features of MB during the interaction with the components of the TME could be considered as a therapeutic target in order to enhance the immune response to MB together with the impairment of the growth and spreading of the MB itself (255). This targeting can either reduce, or even convert, the immunosuppressive TME to immunostimulatory. The characteristics of the MB TME have been described in detail in several reviews elsewhere (255). Herein, we will point out in evidence the relevance of some components of the TME, such as the angiogenic factors, the role of podoplanin (PDPN) in MB spreading, “do not eat me” signals, and the interaction between the astrocytes and MB cells (**Figure 5**). This is to show some of the key cellular and molecular players against which it is conceivable to use old and new immunotherapeutic tools as antibodies or CAR-T cells to further improve the outcome of MB patients.

#### 4.5.1 Angiogenesis and novel factors involved in the regulation of MB spreading

The TME is responsible for the presence of several growth factors that can allow the generation of new blood vessels, such as vascular endothelial growth factor (VEGF) that in turn are essential for the growth and possible spreading of cancer cells (237, 238). The limitation of tumor angiogenesis may help the efficacy of immunotherapy by limiting the growth of tumor cells. For example, the neo-angiogenesis in MB is associated with the most aggressive Group 3 (256). The level of mRNA coding for VEGF-A was markedly increased in this subgroup compared to the other molecular subgroups, correlating negatively with the OS of patients. Furthermore, in rat models, the increased vascularity was associated with less survival. Group 3 of MB is strongly associated with the amplification of MYC and this amplification is linked to the expression of VEGF-A in several solid tumors, including colon rectal carcinomas, breast cancer and gliomas (257–261). This is not surprising, as MYC is involved in the regulation of many genes (262). By consequence, the targeting of angiogenesis is possible by interfering with MYC transcription and molecular target of rapamycin (mTOR) translation with small molecule inhibitors demonstrating synergistic antitumoral effects against MYC-dependent driven MB both *in vitro* and *in vivo* models (263). Also, MB cells express several factors responsible for angiogenesis besides VEGFA, such as VEGFB, VEGFC, VEGF189, VEGF165, VEGF121, angiopoietin (Ang)1, Ang2, transforming growth factor (TGF)α, and basic fibroblast growth factor (bFGF) (264, 265).

The antiangiogenic therapy using the anti-VEGFA mAb bevacizumab did not lead to consistent improvements of OS and PFS, but increased its tolerability together with irinotecan (plus or not with temozolomide) (266). Similarly, the association of intravenous bevacizumab, intraventricular therapy and oral etoposide and cytarabine alternate to oral administration of thalidomide, fenofibrate, and celecoxib was well-tolerated and better responses (although limited in time, about six months) have been detected (267). However, these results are far to be considered an advancement in treatment. It is evident that the complex interactions among these factors and the possibility that different portions of the tumor mass can express different factors do not allow a precise targeting of vascularization of the MB. Furthermore, the recent finding that the VEGFC may negatively regulate the metastatic properties of MB would suggest that not all the factors involved show a pro-tumor effect (102). In fact, VEGFC is involved in lymphangiogenesis, and it has been shown that VEGFC can decrease proliferation and migration of MB cells, inhibiting the formation of pseudo-vessels *in vitro*. Further, irradiation of resistant MB cells with strong expression of VEGFC inhibits the formation of vessel-like *in vitro*, and these irradiated cells generated smaller tumors in nude mice (98). In detail, bioinformatic analysis of several databases of MB suggested that the lower the level of VEGFC expression in the WNT MB group, the worse the outcome of patients. While it was detected the opposite analyzing the SHH, 3 and 4 subgroups in which high levels of VEGFC corresponded to a worse prognosis. This finding would suggest the dual function of VEGFC in MB. In addition, using some *in vitro* models of MB, some experimental evidence supports that the overexpression of VEGFC/VEGFC receptor axis is not only associated with lymphangiogenesis but exerts a beneficial effect in pediatric MB. This finding is based on the use of few cell lines compared to the large array of cell lines present, indicating that these results should be considered with attention (102). It has been shown that the irradiation of DAOY and HDMB03 cell lines increases their epithelial phenotype compared to mesenchymal phenotype, reducing the ability to disseminate like in glioblastoma (268). This would imply that VEGFC-reducing proliferation/migration of MB cells could keep the tumor in a condition more prone to being attacked by antitumor immune cells. This would be typical of MB of the WNT group, while in group 3 the excess of VEGFC would lead to a too strong generation of lymphatic vessels and consequently to metastasis. It is clear that these findings should be taken cautiously because they have been demonstrated with some MB cell lines. However, they can be the explanation of the role of the immune system in checking MB growth. Furthermore, it appears that the expression of VEGFC could be essential to favor immune response against MB at least in the early stages of development of the tumor. These findings would suggests that to plan efficient therapies for MB, combo therapies to TME and triggering of immune response are necessary. Also, it has been reported that B7H3 expression levels are relevant in promoting angiogenesis; this angiogenesis can be inhibited by miR-29 overexpression, leading to a downregulation of B7H3 (242). Thus, the targeting of B7H3 can hit MB cells and MB-associated angiogenesis.

#### 4.5.2 Podoplanin as a potential target in MB

The role of PDPN in brain cancers has been reviewed recently (269). Briefly, it is expressed on several cell types of different embryonal origins, and it is involved in many processes related to brain system development and diseases. These processes, such as thrombosis, lymphangiogenesis, angiogenesis and inflammation, play a key role in regulating the growth of any tumor, including the MB. PDPN can be considered a marker of neoplasia trending to generate metastases (270, 271). Indeed, it is associated with malignant progression leading to epithelial-mesenchymal transition (EMT) and consequent tumor tissue diffusion and metastasis (272–274). This has been demonstrated in a particular mouse model with breast carcinoma cells, or with the antibody NZ-1 anti-PDPN for lung metastatization of CHO-expressing human (h)PDPN, or using point-mutated hPDPN-expressing CHO cells. The PDPN is present on lymphatic endothelial cells in the lumen only in aggressive MB, while in the absence of metastasis this marker was diffusely expressed on the whole surface of endothelial cells. Importantly, PDPN is upregulated in several types of cells during inflammation, including epithelial cells, fibroblasts, fibroblast-like reticular cells, APC and T helper cells interacting with several potential ligands, among which C-type lectin domain family 1 member B (CLEC1B/CLEC-2), CD44 and galectin 8 (274–276).

It has been shown in mice xenografts that the MB cell lines D283-Med, D425-Med and DAOY were sensitive to a novel recombinant single-chain antibody variable region fragment (scFv) of NZ-11 (anti-PDPN antibody) fused to Pseudomonas exotoxin A with a C-terminal KDEL peptide (NZ-1-PE38KDEL). This immunotoxin was further stabilized with a disulfide bond to generate the NZ-1-(scdsFv)-PE38KDEL complex, displaying good stability at 37°C. This construct exerted a strong cytotoxic effect *in vitro* against MB xenograft cells; it delayed markedly the *in vivo* subcutaneous growth of the D283-Med xenograft and, more importantly, it increased by over 40% the OS when administered to mice with intracranial MB tumor xenograft. Also, CAR-T cells directed to PDPN by a construct composed of NZ-1-based single-chain variable fragments and CD28, 4-1BB and CD3 ζ intracellular domains have shown good efficiency against glioma cells both *in vitro* and *in vivo* glioma xenografts (277). Altogether, these findings suggest that PDPN could be considered a target in MB by using CAR-T cells, antibodies and lectins as shown for several types of cancers (257). Conceivably, the elimination of PDPN^+^ cells in the MB TME may reduce the immunosuppression exerted by different types of mesenchymal stromal cells allowing the triggering of NK or T cell responses as shown in other tumor models (278–281).

#### 4.5.3 Do not eat me signaling and MB growth

Among the several surface receptors involved in the regulation of antitumor immunity, the relevance of CD47 is well-established (262). This is also true for the MB (282–285). The CD47, also known as integrin-associated protein (IAP), binds to thrombospondin 1 (TSP-1) and signal regulatory protein alpha (SIRP-α). The binding of CD47 with SIRP-α on macrophages gives a “don’t eat me” signal that can spare the expressing CD47 cells. Typically, CD47 can allow tumor cells to evade macrophage-mediated elimination; consequently, the use of specific anti-CD47 antibodies can restore this event, and this is the rationale for the anti-CD47 antibody used in tumor therapy (282). In the context of MB, it has been recently shown that the humanized anti-CD47 antibody termed Hu5F9-G4 shows therapeutic efficacy on orthotopic PDX models. In addition, the intracranial administration of Hu5F9-G4 can inhibit the dissemination of MB to leptomeninges while exerting minimal effects on healthy neural cells (158). In more detail, the MB of group 3 expresses well the CD47 at the cell surface (86-99% of cells (158); this expression was evident on primary and xenograft-derived cells. More importantly, the use *in vitro* and *in vivo* of Hu5F9-G4 led to strong phagocytosis.

Two primary and three MYC-amplified cell lines localized at the cerebellum and disseminated at leptomeninges have been treated with Hu5F9-G4 antibody (also named Magrolimab) intraperitoneally, and a reduction of tumor burden and increase of OS in mice have been detected. Importantly, MB tumors were consistently infiltrated with macrophages in animals treated with the antibody, while the diffusion to leptomeninges of MB cells was strongly reduced. The metastatic recurrence of MYC overexpressing MB is a major fatal event that can hit pediatric patients (286). Strikingly, the use of Hu5F9-G4 was able to clear spinal metastasis in this xenograft model using MYC-amplified MB cells, and this antibody targets CD15^+^ CSC. The administration into the blood of Hu5F9-G4 revealed that it can pass through the blood-brain endothelial barrier (BBB) apparently even when the tumor was not present. Overall, these findings strongly suggest that the targeting of CD47 on MB cells can affect the growth and diffusion of MB within the CNS. As for PDPN targeting, the relief of the inhibitory signal delivered through the CD47-SIRP-α binding on macrophages could generate a pro-inflammatory TME favoring the anti-MB immune response, as indicated for other tumor types (287, 288).

#### 4.5.4 MB and astrocytes

Astrocytes are a star-shaped subtype of glial cells supporting the neuronal development, metabolism and function of the brain (289–292). They can express glial fibrillary acidic protein (GFAP) but also GFAP-negative astrocytes can be identified, indicating they are a quite heterogenous cell population (290–294). Astrocytes can play a role in innate immunity in the brain by interacting with microglia and undergoing proliferation and activation induced by danger-associated molecular patterns and/or pathogen-associated molecular patterns (293–295). The molecular analysis of astrocytes at the single cell level has identified that several other subsets of this cell population can exist in specific brain regions (296, 297). In addition, it has been reported that astrocytes present in the cerebellum are different from those of other brain regions (274). Detailed reviews on the protein markers of astrocytes present in different regions of the CNS as well as during inflammation and diseases have been published (268, 297, 298). Overall, the astrocytes play a trophic role of MB TME as they secrete the ligand sonic hedgehog (SHH) and this factor triggers the expression of nestin in MB cells; the elimination of MB-associated astrocytes led to suppression of nestin expression, blocking the tumor growth (299). This finding supports the notion that astrocytes are essential for the expansion of MB of the SHH-subgroup (300–303). Furthermore, the secretion of the CCL2 chemokine by astrocytes is essential for the stemness of MB cells and the generation of metastases throughout the CNS (304, 305). The impairment of astrocyte-mediated functions or their elimination by specific targeting may relieve the immune downregulation increasing the effect of immunotherapy.

## 5 Overall considerations and future/putative therapies and immunotherapies for MB

The MB is an aggressive tumor characterized by a relevant molecular and cellular heterogeneity The advances in genomic and transcriptomic have identified several subtypes within the four subgroups (52, 56, 65, 71, 74, 79, 88, 306). This can give insights to modify the therapeutic approaches in favorable and very high-risk groups (58, 306–308). There is no doubt that the molecular classifications have improved the prediction of the clinical outcome, and they provide key markers for a therapy tailored to the risk group. Nevertheless, the old and new therapeutic approaches do not have a striking effect on the OS rate of MB patients, indicating that these attempts should be improved. Recently, some relevant advances in tumor therapy have been reached with the use of immune checkpoint inhibitors (ICI) (178–180, 309). To relieve of the brake of tumor immunity related to the exhaustion of antitumor-specific T lymphocytes should be considered as a requisite to justify therapy with ICI (178–180). The use of pembrolizumab, an anti-PD1 antibody, is under investigation in some clinical trials (NCT02359565 and NCT06514898) in recurrent groups 3 and 4, but results have not been published yet. One report on the treatment of MB considers as ICI the anti-PD1 antibody nivolumab with or without the anti-CTLA4 antibody ipilimumab in 15 patients each (310). An evident difference in OS was present between the MB treated with the combo of nivolumab and ipilimumab (85%) versus nivolumab (40%) only. Unfortunately, this difference was not present after 24 months, showing about 35% of OS for both treatments. This discrepancy could be justified with the subgroups of the MB treated, but this detail is not reported in the original article (310) Also, using nivolumab in a little series of CNS tumors (with just one MB), it appears that a difference in the median survival was associated with the expression of PDL1 and high tumor mutation burden (311). This finding would suggest that the high mutation burden possibly associated with the generation of neoantigens and TAA together with the expression of the ligand of PD1on tumor cells are requisites to elicit a response in CNS tumors. Nevertheless, the results obtained are far from exciting. In this context, a key point is to define whether the MB expresses the ligands for IC molecules such as PDL1 and/or CD80/CD86 antigens. Indeed, it has been shown that the TME in MB shows a variable expression of ligands for IC (312–318). In detail, using K-means clustering, it has been shown that the immune cell infiltration was low in all the four groups of MB while the PD1/PDL1 expression was absent in the cohort analyzed of 19 MB (314). This finding is in agreement with other reports (38, 284, 317), but the PDL1 was markedly expressed in single WNT or SHH cases (312). This would suggest that the therapy with anti-classical IC (CTLA4 and PD1) is not appropriate as there is no possibility to relive the brake of the immune system. It is to be noted that several others IC receptors can be expressed by effector lymphocytes (142, 319–323) and further studies should show how much these additional IC are involved in the impairment of the MB recognition. On the other hand, favoring a pro-inflammatory MB TME, instead of an immunoregulatory one, can trigger the upregulation of ligands for classical IC molecules justifying their use for treatment (322, 323).

The definition of an atlas of naturally presented T cell antigens from 28 MB upon mapping of the HLA ligandome (WNT (n = 4); SHH (n = 9); Group 3 (n = 7); Group 4 (n = 8)) could help to the further characterization of how to target efficiently MB cells (318). It is determinant that the putative antigens show peptides presented in the context of both HLA-I and HLA-II. Indeed, a few candidate proteins: IGFBPL1, INSM1, and INSM2 satisfied this condition. The clinical relevance of the CD4^+^ T helper cells is considered a key point to elicit a durable and efficient anti-MB response. It is of note that the demonstration of the potential immunogenicity of these peptides has been obtained by tetramer staining (318) not by a functional assay. However, this atlas can facilitate the development of mRNA vaccines, primed DC with the peptides identified, and the possible adoptive transfer of antigen-specific T cells. Also, the targeting with the CT-179 drug of a basic helix-loop-helix (bHLH) transcription factor (TF) OLIG2 can suppress the recurrence of the MB of the SHH subgroup (324). Interestingly, OLIG2 promotes differentiation in oligodendrocyte lineage, but it favors the maintenance of an undifferentiated condition of neural stem cells and progenitor cells (325–328).

The block of the dimerization, phosphorylation and DNA binding of TF leads to prolonged survival of SHH-MB PDX and GEM models and potentiates radiotherapy (RT) *in vivo*. These findings could suggest the targeting of OLIG2^+^ CSCs that remain quiescent upon conventional therapy with a better clinical outcome for some patients. This would indicate that the targeting of appropriate TF regulating the fate of CSCs is a key tool to avoid resistance to therapy and relapse. The CT-179 increases the differentiation and upregulates the cyclin-dependent kinase (CDK) 4, favoring the proliferation. The further combination with the CDK4/6 inhibitor palbociclib prolongs the survival of the PDX and GEM models. Altogether, these findings indicate that the targeting of the TF in combination with other drugs can improve the clinical outcome. However, it should be considered the role of OLIG2 as a master regulator of oligodendrocyte lineage in early stages of differentiation as well as other components of the same family of TF (325). It is still undefined whether the blocking of one TF does not influence the others and how much the blocking of OLIG2 during the maturation of the CNS can affect its correct development. The treatment of a tumor during the development of the CNS is conceivably a challenging condition that is not so relevant if the tumor has been developed in an adult (325). Certainly, the targeting of OLIG2 is feasible for gliomas in adults, but it should be noted that the degree of expression can vary in glioblastomas and astrocytoma while it is usually well expressed in oligodendrogliomas (325). The use of OLIG2 as a target for MB therapy should be considered cautiously as OLIG2 involved in the motor neuron fate and in neural repair as well (327, 328). It is conceivable that the combo of inhibitors of OLIG2 TF and immunotherapy can increase the efficiency of lymphocytes in eliminating CSC. This targeting has been demonstrated for the anti-MB protogenin (PRTG) CAR-T cells recognizing CSC of MB group 3 with a strong expression of PRTG (PRTG^high^) and for the anti-PRTG blocking antibodies (329). Altogether, these findings suggest that the precise identification of the stem cells of MB could be the future target of the therapy. Another point to be discussed is the possible use of combinatiotn of immunotherapy and chemotherapy. It has been shown the association of chemotherapy and adoptive immunotherapy is more efficient in eliminating MB cells bot *in vitro* and *in vivo* subcutaneously injected MB cell line DAOY (330). It is to be noted that in this experimental system the addition of LPS can further increase the anti-MB effect of the combo therapy, indicating that the activation of antigen presenting cells could be involved.

## 6 Conclusions

MB is an aggressive pediatric tumor, and although great advances have been reached, the present immunotherapeutic tools are not enough to modify the outcome of this disease. The detailed study of the expression of receptors on immune cells together with that of ligands on MB cells as well as other components of the TME could suggest the main molecules to be targeted by immunotherapy. MB groups show some relevant differences for the expression of ligands of several activating receptors of antitumor effector cells, immunecheckpoint molecules, MHC-I and II as well as inflammatory cytokines (**Supplementary Figures 1, 2**) (72, 331, 332). However, there is no a unique pattern of expression of these molecules for each subtype suggesting that each patient should be considered as a specific case. Also, there is a certain heterogeneity regarding the degree and type of immune cell infiltration (**Supplementary Figure 3**) (72) among the different groups of MB. This suggests that a tailored therapy is necessary on the basis of the results of molecular analysis. The identification of specific markers of MB stem cells could allow the study of the counter receptors expressed on immune cells to trigger the elimination of the source of the disease. Furthermore, the TME influencing MB and immune cells could influence the effectiveness of immunotherapy modifying the expression of the molecules involved in the recognition of MB cells by anti-tumor effector cells. Further studies focused on new effector cell subsets, novel antibodies and ADC, besides those listed in **Table 3**, and combination of drugs with immunotherapeutic tools may open new avenues to fight this tumor.

**Table 3:** Monoclonal antibodies therapies targeted to some receptors expressed on MB cells and associated tumor microenvironment.

Target molecule	Antibody name	Type of antibody	Functional effect on target cells	Functional effect on immune cells	Tumor target	Reference
GD2	^131^I-3F8	IGG3	Killing target cell	Not done	Medulloblastoma patients	(144)
B7H3	8H9 humanized	IGG1	Blocking cell proliferation	ADCC	Neuroblastoma LAN-1 cell line	(145)
B7H3	MGA271 Fully human	IGG1	Blocking cell proliferation	ADCC	ND Renal and bladder carcinoma	(146)
B7H3	MJ18 rat	IGG1	Blocking cell proliferation	CD8+cell infiltration and killing target cell	Pancreatic cancer	(147)
B7H3xCD3 nanorings	8H9 scFv	bispecific	Killing target cell Independent from HLA-I	Increase T lymphocytes memory phenotype	medulloblastoma	(148)
B7H3xCD16 Bike	8H9 scFv anti-CD16 scFv 3G8	bispecific	Killing target cell Altered glucose metabolism	ADCC NK cell killing	Non-small cell lung cancer	(149)
B7H3x4-1BB Bike	mAb B5 murine mAb ID8 murine	bispecific	Blocking cell proliferation	Costimulation Upregulation activation antigens, IFNγ release CD8	Murine models CT26 MC38 B16-F10	(150)
B7H3 duocarmycin ADC	PRCA157 mAb MGCO18	IGG1	Killing target cell	Not done	PDX of head and neck, prostate, breast carcinoma Toxicity tests Cynomolgus	(151)
B7H3 DNA Topoisomerase I ADC	DS-7300a	IGG1	Killing tumor cell	Not done	PDX small lung cancer, non-small cell lung cancer, head and neck. bladder	(152)
B7H3 Monomethyl auristatin (MMA) E- and pyrrolobenzodiazepine ADCs	m276 fully humanized	IGG1	MMAE kills target cell Pyrrolobenzodiazepine kills medulloblastoma and endothelial cells	Not done	Murine models B16, MC38, Py230), 4T1 DLD-1, HCT-116, KM12, KM12SM, MDA--231, HT29, DMS-273 SW620, UACC CB17 OVCAR3	(153)
B7H3-DM1 ADC	14A2	Not determined	Killing target cells	Not done	Craniopharyngioma organoids	(154)
EGFR	Cetuximab + neuromedin B receptor antagonist BIM-23127	IGG1	Inhibition DAOY proliferation	ADCC	Medulloblastoma models	(155)
HER2	Trastuzumab Deruxtecan	IGG1	Killing target cells	Not done	Reduction brain metastasis of refractory breast carcinoma	(156)
PDPN	NZ-1 NZ-1-(scdsFv)-PE38KDEL	IGG1k	Killing target cells	Not done	Glioblastoma	(157)
CD47	Hu5F9-G4	IGG4	Blocking interaction CD47 and SIRP1alpha Increase phagocytosis	No ADCC	Primary, metastatic, recurrent glioblastoma	(158)
VEGF-A	Bevacizumab combo with Temozolomide irinotecan	IGG1k	antiangiogenic	Not done	Medulloblastoma patients	(159)

GD2, disialoganglioside, B7H3, B7 homolog 3; DM1, mertansine/emtansine; EGFR, epidermal growth factor receptor; HER2, human epidermal growth factor receptor 2; PDPN, podoplanin; VEGF-A, vascular endothelial growth factor-A; ADC, antibody drug conjugate; ADCC, antibody dependent cellular cytotoxicity.

## References

[1] Taylor MD Northcott PA Korshunov A Remke M Cho YJ Clifford SC. Molecular subgroups of medulloblastoma: The current consensus. Acta Neuropathologica. (2012) 123:465–72. doi: 10.1007/s00401-011-0922-z PMC3306779 22134537

[2] Louis DN Perry A Reifenberger G von Deimling A Figarella-Branger D Cavenee WK. The 2016 world health organization classification of tumors of the central nervous system: a summary. Acta Neuropathol. (2016) 131:803–20. doi: 10.1007/s00401-016-1545-1 27157931

[3] Michalski JM Janss AJ Vezina LG Smith KS Billups CA Burger PC. Children’s oncology group phase III trial of reduced-dose and reduced-volume radiotherapy with chemotherapy for newly diagnosed average-risk medulloblastoma. J Clin Oncol. (2021) 39:2685–97. doi: 10.1200/JCO.20.02730 PMC8376317 34110925

[4] Gupta T Pervez S Dasgupta A Chatterjee A Epari S Chinnaswamy G. Omission of upfront craniospinal irradiation in patients with low-risk WNT-pathway medulloblastoma is associated with unacceptably high risk of neuraxial failure. Clin Cancer Res. (2022) 28:4180–5. doi: 10.1158/1078-0432.CCR-22-0758 35653134

[5] Baroni LV Sampor C Gonzalez A Lubieniecki F Lamas G Rugilo C. Bridging the treatment gap in infant medulloblastoma: Molecularly informed outcomes of a globally feasible regimen. Neuro-Oncology. (2020) 22:1873–81. doi: 10.1093/neuonc/noaa122 PMC7746938 32413139

[6] Gajjar A Chintagumpala M Ashley D Kellie S Kun LE Merchant TE. Risk-adapted craniospinal radiotherapy followed by high-dose chemotherapy and stem-cell rescue in children with newly diagnosed medulloblastoma (St Jude Medulloblastoma-96): long-term results from a prospective, multicentre trial. Lancet Oncol. (2006) 7:813–20. doi: 10.1016/S1470-2045(06)70867-1 17012043

[7] Ji Y Xiao C Fan T Deng Z Wang D Cai W. The epigenetic hallmarks of immune cells in cancer. Mol Cancer. (2025) 24:66. doi: 10.1186/s12943-025-02255-4 40038722 PMC11881328

[8] Nihira NT Kudo R Ohta T. Inflammation and tumor immune escape in response to DNA damage. Semin Cancer Biol. (2025) 110:36–45. doi: 10.1016/j.semcancer.2025.02.005 39938581

[9] Hu Y Zhou T Cai P He Z. Neoantigens: new hope for cancer therapy. Front Oncol. (2025) 15:1531592. doi: 10.3389/fonc.2025.1531592 40134605 PMC11932895

[10] Baharom F Hermans D Delamarre L Seder RA. Vax-Innate: improving therapeutic cancer vaccines by modulating T cells and the tumour microenvironment. Nat Rev Immunol. (2025) 25:195–211. doi: 10.1038/s41577-024-01091-9 39433884

[11] de Vries NL van de Haar J Veninga V Chalabi M Ijsselsteijn ME van der Ploeg M. γδ T cells are effectors of immunotherapy in cancers with HLA class I defects. Nature. (2023) 613:743–50. doi: 10.1038/s41586-022-05593-1 PMC9876799 36631610

[12] Conforti F Pala L Di Mitri D Catania C Cocorocchio E Laszlo D. Sex hormones, the anticancer immune response, and therapeutic opportunities. Cancer Cell. (2025) 43:343–60. doi: 10.1016/j.ccell.2025.02.013 40068594

[13] Sun M Angelillo J Hugues S. Lymphatic transport in anti-tumor immunity and metastasis. J Exp Med. (2025) 222:1–19. doi: 10.1084/jem.20231954 PMC11837853 39969537

[14] Wang TW Nakanishi M. Immune surveillance of senescence: potential application to age-related diseases. Trends Cell Biol. (2025) 35:248–57. doi: 10.1016/j.tcb.2024.06.007 39025762

[15] Mellman I Chen DS Powles T Turley SJ. The cancer-immunity cycle: Indication, genotype, and immunotype. Immunity. (2023) 56:2188–205. doi: 10.1016/j.immuni.2023.09.011 37820582

[16] Xia Y Huang C Zhong M Zhong H Ruan R Xiong J. Targeting HGF/c-MET signaling to regulate the tumor microenvironment: Implications for counteracting tumor immune evasion. Cell communication signaling: CCS. (2025) 23:46. doi: 10.1186/s12964-025-02033-1 39856684 PMC11762533

[17] Guo R Wang R Zhang W Li Y Wang Y Wang H. Macrophage polarisation in the tumour microenvironment: recent research advances and therapeutic potential of different macrophage reprogramming. Cancer Control. (2025) 32:1–19. doi: 10.1177/10732748251316604 PMC11758544 39849988

[18] Rosenberg SA Lotze MT Muul LM Leitman S Chang AE Ettinghausen SE. at.Observations on the systemic administration of autologous lymphokine-activated killer cells and recombinant interleukin-2 to patients with metastatic cancer. N Engl J Med. (1985) 313:1485–92. doi: 10.1056/NEJM198512053132327 3903508

[19] Parmiani G. An explanation of the variable clinical response to interleukin 2 and LAK cells. Immunol Today. (1990) 11:113–5. doi: 10.1016/0167-5699(90)90046-c 2187466

[20] Aebersold P Hyatt C Johnson S Hines K Korcak L Sanders M. Lysis of autologous melanoma cells by tumor-infiltrating lymphocytes: association with clinical response. J Natl Cancer Inst. (1991) 83:932–7. doi: 10.1093/jnci/83.13.932 2067036

[21] Puri RK Rosenberg SA. Combined effects of interferon α and interleukin 2 on the induction of a vascular leak syndrome in mice. Cancer Immunol Immunotherapy. (1989) 28:267–74. doi: 10.1007/BF00205236 PMC11038677 2495179

[22] Fujita S Puri RK Yu ZX Travis WD Ferrans VJ. An ultrastructural study of *in vivo* interactions between lymphocytes and endothelial cells in the pathogenesis of the vascular leak syndrome induced by interleukin-2. Cancer. (1991) 68:2169–74. doi: 10.1002/1097-0142(19911115)68:10<2169::aid-cncr2820681014>3.0.co;2-f 1913455

[23] Pol JG Caudana P Paillet J Piaggio E Kroemer G. Effects of interleukin-2 in immunostimulation and immunosuppression. J Exp Med. (2020) 217:e20191247. doi: 10.1084/jem.20191247 31611250 PMC7037245

[24] León K García-Martínez K Carmenate T Rojas G. Combining computational and experimental biology to develop therapeutically valuable IL2 muteins. Semin Oncol. (2018) 45:95–104. doi: 10.1053/j.seminoncol.2018.04.001 30318089

[25] Balkhi S Bilato G De Lerma Barbaro A Orecchia P Poggi A Mortara L. Efficacy of anti-cancer immune responses elicited using tumor-targeted IL-2 cytokine and its derivatives in combined preclinical therapies. Vaccines. (2025) 13:1–24. doi: 10.3390/vaccines13010069 PMC11768832 39852848

[26] Bock TJ Colonne CK Fiorenza S Turtle CJ. Outcome correlates of approved CD19-targeted CAR T cells for large B cell lymphoma. Nat Rev Clin Oncol. (2025) 22:241–61. doi: 10.1038/s41571-025-00992-5 39966627

[27] Haubner S Subklewe M Sadelain M. Honing CAR T cells to tackle acute myeloid leukemia. Blood. (2025) 145:1113–25. doi: 10.1182/blood.2024024063 39630061

[28] Sabahi M Fathi Jouzdani A Sadeghian Z Dabbagh Ohadi MA Sultan H Salehipour A. CAR-engineered NK cells versus CAR T cells in treatment of glioblastoma; strength and flaws. J Neurooncol. (2025) 171:495–530. doi: 10.1007/s11060-024-04876-z 39538038

[29] Khalifeh M Salman H. Engineering resilient CAR T cells for immunosuppressive environment. Mol Ther. (2025) S1525-0016:S1525–0016(25)00039-5. doi: 10.1016/j.ymthe.2025.01.035 PMC12172186 39863931

[30] Wang DY Salem JE Cohen JV Chandra S Menzer C Ye F. Fatal toxic effects associated with immune checkpoint inhibitors: A systematic review and meta-analysis. JAMA Oncol. (2018) 4:1721–8. doi: 10.1001/jamaoncol.2018.3923 PMC6440712 30242316

[31] Blum SM Rouhani SJ Sullivan RJ. Effects of immune-related adverse events (irAEs) and their treatment on antitumor immune responses. Immunol Rev. (2023) 318:167–78. doi: 10.1111/imr.13262 37578634

[32] Schubert ML Schmitt M Wang L Ramos CA Jordan K Müller-Tidow C. Side-effect management of chimeric antigen receptor (CAR) T-cell therapy. Ann Oncol. (2021) 32:34–48. doi: 10.1016/j.annonc.2020.10.478 33098993

[33] Joseph T Sanchez J Abbasi A Zhang L Sica RA Duong TQ. Cardiotoxic effects following CAR-T cell therapy: A literature review. Curr Oncol Rep. (2025) 27:135–47. doi: 10.1007/s11912-024-01634-2 PMC11861112 39836349

[34] Umair MM Lai X Xue YB Yao H. Influence of CAR T-cell therapy associated complications. Front Oncol. (2025) 15:1494986. doi: 10.3389/fonc.2025.1494986 40052127 PMC11882432

[35] Javaid A Peres T Pozas J Thomas J Larkin J. Current and emerging treatment options for BRAFV600-mutant melanoma. Expert Rev Anticancer Ther. (2025) 25:55–69. doi: 10.1080/14737140.2025.2451722 39784319

[36] Carr AS Vonberg FW Koay S Young K Shaw H Olsson-Brown A. Neurological complications of immune checkpoint inhibitors: a practical guide. Pract Neurol. (2024) 25:116–26. doi: 10.1136/pn-2024-004327 39592208

[37] Brown LJ Yeo N Gee H Kong BY Hau E da Silva IP. Immune checkpoint inhibitors +/– chemotherapy for patients with NSCLC and brain metastases: A systematic review and network meta-analysis. Thorac Cancer. (2025) 16:1–14. doi: 10.1111/1759-7714.15510 PMC11753865 39843204

[38] Vermeulen JF Van Hecke W Adriaansen EJM Jansen MK Bouma RG Villacorta Hidalgo J. Prognostic relevance of tumor-infiltrating lymphocytes and immune checkpoints in pediatric medulloblastoma. OncoImmunology. (2018) 7(3):e1398877. doi: 10.1080/2162402X.2017.1398877 29399402 PMC5790383

[39] Martin AM Nirschl CJ Polanczyk MJ Bell WR Nirschl TR Harris-Bookman S. PD-L1 expression in medulloblastoma: An evaluation by subgroup. Oncotarget. (2018) 9:19177–91. doi: 10.18632/oncotarget.24951 PMC5922386 29721192

[40] Wang B LeBel A D’Mello AM. Ignoring the cerebellum is hindering progress in neuroscience. Trends Cognit Sci. (2025) 29:318–30. doi: 10.1016/j.tics.2025.01.004 39934082

[41] Massara M Delogu C Cardinale L Livoti V Liso A Cainelli E. The lateralized cerebellum: insights into motor, cognitive, and affective functioning across ages: a scoping review. Berlin Heidelberg: Springer (2025). 122 p. doi: 10.1007/s00415-024-12884-2 39812809

[42] Reumers SFI Bongaerts FLP de Leeuw FE van de Warrenburg BPC Schutter DJLG Kessels RPC. Cognition in cerebellar disorders: What’s in the profile? A systematic review and meta-analysis. J Neurol. (2025) 272:250. doi: 10.1007/s00415-025-12967-8 40047904 PMC11885410

[43] Bailey P Cushing H. Medulloblastoma Cerebelli: a common type of midcerebellar glioma of childhood. Arch Neurol Psychiatry. (1925) 14:192–224. doi: 10.1001/archneurpsyc.1925.02200140055002

[44] Millard NE De Braganca KC. Medulloblastoma. J Child Neurol. (2016) 31:1341–53. doi: 10.1177/0883073815600866 PMC4995146 26336203

[45] Rutkowski S von Hoff K Emser A Zwiener I Pietsch T Figarella-Branger D. Survival and prognostic factors of early childhood medulloblastoma: an international meta-analysis. J Clin Oncol. (2010) 28:4961–8. doi: 10.1200/JCO.2010.30.2299 20940197

[46] Carta R Del Baldo G Miele E Po A Besharat ZM Nazio F. Cancer predisposition syndromes and medulloblastoma in the molecular era. Front Oncol. (2020) 10:566822. doi: 10.3389/fonc.2020.566822 33194646 PMC7658916

[47] Smith MJ Beetz C Williams SG Bhaskar SS O’Sullivan J Anderson B. Germline mutations in SUFU cause Gorlin syndrome-associated childhood medulloblastoma and redefine the risk associated with PTCH1 mutations. J Clin Oncol. (2014) 32:4155–61. doi: 10.1200/JCO.2014.58.2569 25403219

[48] Ostrom QT Gittleman H Truitt G Boscia A Kruchko C Barnholtz-Sloan JS. CBTRUS statistical report: Primary brain and other central nervous system tumors diagnosed in the United States in 2011-2015. Neuro-Oncology. (2018) 20:iv1–iv86. doi: 10.1093/neuonc/noy131 30445539 PMC6129949

[49] Crawford JR MacDonald TJ Packer RJ. Medulloblastoma in childhood: new biological advances. Lancet Neurol. (2007) 6:1073–85. doi: 10.1016/S1474-4422(07)70289-2 18031705

[50] Louis DN Perry A Wesseling P Brat DJ Cree IA Figarella-Branger D. The 2021 WHO classification of tumors of the central nervous system: A summary. Neuro-Oncology. (2021) 23:1231–51. doi: 10.1093/neuonc/noab106 PMC8328013 34185076

[51] Li M Deng Y Zhang W. Molecular determinants of medulloblastoma metastasis and leptomeningeal dissemination. Mol Cancer Res. (2021) 19:743–52. doi: 10.1158/1541-7786.MCR-20-1026 33608450

[52] Aref D Croul S. Medulloblastoma: recurrence and metastasis. CNS Oncol. (2013) 2:377–85. doi: 10.2217/cns.13.30 PMC6166489 25054581

[53] Wang J Garancher A Ramaswamy V Wechsler-Reya RJ. Medulloblastoma: From molecular subgroups to molecular targeted therapies. Annu Rev Neurosci. (2018) 41:207–32. doi: 10.1146/annurev-neuro-070815-013838 29641939

[54] Badiger S Gudipati A Uppin M Konatam ML Yeramneni VK Bhattacharjee S. Clinicomorphological and molecular analysis of medulloblastoma and association with survival: A single tertiary care center experience. J Cancer Res Ther. (2023) 19:S592–602. doi: 10.4103/jcrt.jcrt_1268_22 38384024

[55] Korshunov A Okonechnikov K Sahm F Ryzhova M Stichel D Schrimpf D. Transcriptional profiling of medulloblastoma with extensive nodularity (MBEN) reveals two clinically relevant tumor subsets with VSNL1 as potent prognostic marker. Acta Neuropathol. (2020) 139:583–96. doi: 10.1007/s00401-019-02102-z 31781912

[56] Menon G Nair S Muthurethinam T Krishnakumar K Bhattacharya RN. Medulloblastoma in children: Prognostic factors and predictors of outcome. J Pediatr Neurosci. (2006) 1:16–20. doi: 10.4103/1817-1745.22942

[57] Orr BA. Pathology, diagnostics, and classification of medulloblastoma. Brain Pathol. (2020) 30:664–78. doi: 10.1111/bpa.12837 PMC7317787 32239782

[58] Cotter JA Hawkins C. Medulloblastoma: WHO 2021 and beyond. Pediatr Dev Pathol. (2022) 25:23–33. doi: 10.1177/10935266211018931 35168417

[59] Miller DC. The world health organization classification of tumors of the central nervous system, fifth edition, 2021: A critical analysis. Adv Tech Stand Neurosurg. (2023) 46:1–21. doi: 10.1007/978-3-031-28202-7_1 37318567

[60] Wang YJ Wang P Yan Z Zhou Q Gunturkun F Li P. Advancing presurgical non-invasive molecular subgroup prediction in medulloblastoma using artificial intelligence and MRI signatures. Cancer Cell. (2024) 42:1239–1257.e7. doi: 10.1016/j.ccell.2024.06.002 38942025 PMC13010562

[61] Alharbi M Mobark N Bashawri Y Abu Safieh L Alowayn A Aljelaify R. Methylation profiling of medulloblastoma in a clinical setting permits sub-classification and reveals new outcome predictions. Front Neurol. (2020) 11:167. doi: 10.3389/fneur.2020.00167 32265819 PMC7100767

[62] Chiang J Moreira DC Pytel NJ Liu YC Blackburn PR Shi Z. A CTNNB1-altered medulloblastoma shows the immunophenotypic, DNA methylation and transcriptomic profiles of SHH-activated, and not WNT-activated, medulloblastoma. Neuropathol Appl Neurobiol. (2022) 48:e12815. doi: 10.1111/nan.12815 35320876 PMC9295902

[63] Northcott PA Shih DJH Peacock J Garzia L Sorana Morrissy A Zichner T. Subgroup-specific structural variation across 1,000 medulloblastoma genomes. Nature. (2012) 487:49–56. doi: 10.1038/nature11327 PMC3683624 22832581

[64] Hicks D Rafiee G Schwalbe EC Howell CI Lindsey JC Hill RM. The molecular landscape and associated clinical experience in infant medulloblastoma: prognostic significance of second-generation subtypes. Neuropathol Appl Neurobiol. (2021) 47:236–50. doi: 10.1111/nan.12656 32779246

[65] Zhukova N Ramaswamy V Remke M Pfaff E Shih DJ Martin DC. Subgroup-specific prognostic implications of TP53 mutation in medulloblastoma. J Clin Oncol. (2013) 31:2927–35. doi: 10.1200/JCO.2012.48.5052 PMC4878050 23835706

[66] Kijima N Kanemura Y. Molecular classification of medulloblastoma. Neurol Med Chir (Tokyo). (2016) 56:687–97. doi: 10.2176/nmc.ra.2016-0016 PMC5221779 27238212

[67] Sursal T Ronecker JS Dicpinigaitis AJ Mohan AL Tobias ME Gandhi CD. Molecular stratification of medulloblastoma: clinical outcomes and therapeutic interventions. Anticancer Res. (2022) 42:2225–39. doi: 10.21873/anticanres.15703 35489737

[68] Ramaswamy V Remke M Bouffet E Bailey S Clifford SC Doz F. Risk stratification of childhood medulloblastoma in the molecular era: the current consensus. Acta Neuropathol. (2016) 131:821–31. doi: 10.1007/s00401-016-1569-6 PMC4867119 27040285

[69] Voskamp MJ Li S van Daalen KR Crnko S Ten Broeke T Bovenschen N. Immunotherapy in medulloblastoma: current state of research, challenges, and future perspectives. Cancers (Basel). (2021) 13:5387. doi: 10.3390/cancers13215387 34771550 PMC8582409

[70] Ramaswamy V Taylor MD. Medulloblastoma: from myth to molecular. J Clin Oncol. (2017) 35:2355–63. doi: 10.1200/JCO.2017.72.7842 28640708

[71] Friedman HS Burger PC Bigner SH Trojanowski JQ Brodeur GM He XM. Phenotypic and genotypic analysis of a human medulloblastoma cell line and transplantable xenograft (D341 Med) demonstrating amplification of c-myc. Am J Pathol. (1988) 130:472–84. 3279793 PMC1880676

[72] Cavalli FMG Remke M Rampasek L Peacock J Shih DJH Luu B. Intertumoral heterogeneity within medulloblastoma subgroups. Cancer Cell. (2017) 31:737–754.e6. doi: 10.1016/j.ccell.2017.05.005 28609654 PMC6163053

[73] Hamilton SR Liu B Parsons RE Papadopoulos N Jen J Powell SM. The molecular basis of Turcot’s syndrome. N Engl J Med. (1995) 332:839–47. doi: 10.1056/NEJM199503303321302 7661930

[74] Chen Y Zhang H Zhao Y Ma J. Congenital medulloblastoma in two brothers with SUFU-mutated Gorlin-Goltz syndrome: Case reports and literature review. Front Oncol. (2022) 12:988798. doi: 10.3389/fonc.2022.988798 36313636 PMC9603755

[75] Waszak SM Northcott PA Buchhalter I Robinson GW Sutter C Groebner S. Spectrum and prevalence of genetic predisposition in medulloblastoma: a retrospective genetic study and prospective validation in a clinical trial cohort. Lancet Oncol. (2018) 19:785–98. doi: 10.1016/S1470-2045(18)30242-0 PMC5984248 29753700

[76] Bonfim-Silva R Salomão KB Pimentel TVC de A Menezes CCB de O Palma PVB Fontes AM. Biological characterization of the UW402, UW473, ONS-76 and DAOY pediatric medulloblastoma cell lines. Cytotechnology. (2019) 71:893–903. doi: 10.1007/s10616-019-00332-3 31346954 PMC6787134

[77] Zanini C Ercole E Mandili G Salaroli R Poli A Renna C. Medullospheres from DAOY, UW228 and ONS-76 cells: increased stem cell population and proteomic modifications. PloS One. (2013) 8:1–14. doi: 10.1371/journal.pone.0063748 PMC3663798 23717474

[78] Ivanov DP Coyle B Walker DA Grabowska AM. *In vitro* models of medulloblastoma: Choosing the right tool for the job. J Biotechnol. (2016) 236:10–25. doi: 10.1016/j.jbiotec.2016.07.028 27498314

[79] Gu S Chen K Yin M Wu Z Wu Y. Proteomic profiling of isogenic primary and metastatic medulloblastoma cell lines reveals differential expression of key metastatic factors. J Proteomics. (2017) 160:55–63. doi: 10.1016/j.jprot.2017.03.022 28363815

[80] Othman RT Kimishi I Bradshaw TD Storer LC Korshunov A Pfister SM. Overcoming multiple drug resistance mechanisms in medulloblastoma. Acta Neuropathol Commun. (2014) 2:57. doi: 10.1186/2051-5960-2-57 24887326 PMC4229867

[81] Jacobsen PF Jenkyn JMP DJ. Establishment of a human medulloblastoma cell line and its heterotransplantation into nude mice. J Neuropathology Exp Neurol. (1985) 4:472–85. doi: 10.1097/00005072-198509000-00003 2993532

[82] Scheidt T Alka O Gonczarowska-Jorge H Gruber W Rathje F Dell’Aica M. Phosphoproteomics of short-term hedgehog signaling in human medulloblastoma cells. Cell Commun Signal. (2020) 18:99. doi: 10.1186/s12964-020-00591-0 32576205 PMC7310537

[83] Chai JY Sugumar V Alshawsh MA Wong WF Arya A Chong PP. The role of smoothened-dependent and-independent hedgehog signaling pathway in tumorigenesis. Biomedicines. (2021) 9:1–52. doi: 10.3390/biomedicines9091188 PMC8466551 34572373

[84] Lee SJ Krauthauser C Maduskuie V Fawcett PT Olson JM Rajasekaran SA. Curcumin-induced HDAC inhibition and attenuation of medulloblastoma growth *in vitro* and in *vivo* . BMC Cancer. (2011) 11:592–605. doi: 10.1186/1471-2407-11-144 PMC3090367 21501498

[85] Casciati A Tanori M Manczak R Saada S Tanno B Giardullo P. Human medulloblastoma cell lines: Investigating on cancer stem cell-like phenotype. Cancers. (2020) 12:1–14. doi: 10.3390/cancers12010226 PMC7016648 31963405

[86] Friedman HS Burger PC Bigner SH Trojanowski JQ Wikstrand CJ Halperin EC. Establishment and characterization of the human medulloblastoma cell line and transplantable xenograft D283 med. J Neuropathology Exp Neurol. (1985) 44:592–605. doi: 10.1097/00005072-198511000-00005 4056828

[87] Pitolli C Marini A Guerra M Pieraccioli M Marabitti V Palluzzi F. MYC up-regulation confers vulnerability to dual inhibition of CDK12 and CDK13 in high-risk Group 3 medulloblastoma. J Exp Clin Cancer Res. (2023) 42:1–19. doi: 10.1186/s13046-023-02790-2 37599362 PMC10440921

[88] Schwalbe EC Lindsey JC Nakjang S Crosier S Smith AJ Hicks D. Novel molecular subgroups for clinical classification and outcome prediction in childhood medulloblastoma: a cohort study. Lancet Oncol. (2017) 18:958–71. doi: 10.1016/S1470-2045(17)30243-7 PMC5489698 28545823

[89] Nazio F Po A Abballe L Ballabio C Diomedi Camassei F Bordi M. Targeting cancer stem cells in medulloblastoma by inhibiting AMBRA1 dual function in autophagy and STAT3 signalling. Acta Neuropathologica. (2021) 142:537–64. doi: 10.1007/s00401-021-02347-7 PMC8357694 34302498

[90] Jenkins NC Rao G Eberhart CG Pedone CA Dubuc AM Fults DW. Somatic cell transfer of c-Myc and Bcl-2 induces large-cell anaplastic medulloblastomas in mice. J Neuro-Oncology. (2016) 126:415–24. doi: 10.1007/s11060-015-1985-9 PMC4733589 26518543

[91] Kling MJ Kesherwani V Mishra NK Alexander G McIntyre EM Ray S. A novel dual epigenetic approach targeting BET proteins and HDACs in Group 3 (MYC-driven) Medulloblastoma. Lab Invest. (1991) 64:833–43. doi: 10.1186/s13046-022-02530-y PMC9650837 36357906

[92] Keles GE Berger MS Srinivasan J Kolstoe DD Bobola MS Silber JR. Establishment and characterization of four human medulloblastoma-derived cell lines. Oncol Res. (1995) 7:493–503. 8866661

[93] Menyhárt O Győrffy B. Principles of tumorigenesis and emerging molecular drivers of SHH-activated medulloblastomas. Ann Clin Trans Neurol. (2019) 6:990–1005. doi: 10.1002/acn3.762 PMC6529984 31139698

[94] Singh A Cheng D Swaminathan J Yang Y Zheng Y Gordon N. REST-dependent downregulation of von Hippel-Lindau tumor suppressor promotes autophagy in SHH-medulloblastoma. Sci Rep. (2024) 14:1–13. doi: 10.1038/s41598-024-63371-7 38866867 PMC11169471

[95] Coyle B Kessler M Sabnis DH Kerr ID. ABCB1 in children’s brain tumours. Biochem Soc Trans. (2015) 43:1018–22. doi: 10.1042/BST20150137 26517917

[96] Wei Y Maximov V Morrissy SA Taylor MD Pallas DC Kenney AM. P53 function is compromised by inhibitor 2 of phosphatase 2A in sonic hedgehog medulloblastoma. Mol Cancer Res. (2019) 17:186–98. doi: 10.1158/1541-7786.MCR-18-0485 PMC6318027 30224541

[97] Tamura K Shimizu K Yamada M Okamoto Y Matsui Y Park KC. Expression of major histocompatibility complex on human medulloblastoma cells with neuronal differentiation. Cancer Res. (1989) 49:5380–4. doi: 10.1158/1541-7786.MCR-18-0485 2504489

[98] Bassani B Bartolini D Pagani A Principi E Zollo M Noonan DM. Fenretinide (4-HPR) targets caspase-9, ERK 1/2 and the wnt3a/β-catenin pathway in medulloblastoma cells and medulloblastoma cell spheroids. PloS One. (2016) 11:e0154111. doi: 10.1371/journal.pone.0154111 27367907 PMC4930187

[99] Van Essen MJ Nicheperovich A Schuster-böckler B Esther BE Jacob J. Sonic hedgehog medulloblastoma cells in co-culture with cerebellar organoids converge towards *in vivo* Malignant cell states. Neurooncol Adv. (2024) 7(1):vdae218. doi: 10.1101/2024.04.01.587603 39896075 PMC11783571

[100] Mascaro-Cordeiro B Oliveira ID Tesser-Gamba F Pavon LF Saba-Silva N Cavalheiro S. Valproic acid treatment response *in vitro* is determined by TP53 status in medulloblastoma. Childs Nerv Syst. (2018) 34:1497–509. doi: 10.1007/s00381-018-3817-7 29785653

[101] Milde T Lodrini M Savelyeva L Korshunov A Kool M Brueckner LM. HD-MB03 is a novel Group 3 medulloblastoma model demonstrating sensitivity to histone deacetylase inhibitor treatment. J Neuro-Oncology. (2012) 110:335–48. doi: 10.1007/s11060-012-0978-1 23054560

[102] Penco-Campillo M Comoglio Y Feliz Morel ÁJ Hanna R Durivault J Leloire M. VEGFC negatively regulates the growth and aggressiveness of medulloblastoma cells. Commun Biol. (2020) 3:1–14. doi: 10.1038/s42003-020-01306-4 33067561 PMC7568583

[103] Jonchere B Williams J Zindy F Liu J Robinson S Farmer DM. Combination of ribociclib with BET-Bromodomain and PI3K/mTOR inhibitors for medulloblastoma treatment in vitro and *in vivo* . Mol Cancer Ther. (2023) 22:37–51. doi: 10.1158/1535-7163.MCT-21-0896 PMC9808370 36318650

[104] He XM Wikstrand CJ Friedman HS Bigner SH Pleasure S Trojanowski JQ. Differentiation characteristics of newly established medulloblastoma cell lines (D384 Med, D425 Med, and D458 Med) and their transplantable xenografts. Lab Invest. (1991) 64(6):833–43. 1904513

[105] Ampudia-Mesias E Cameron CS Yoo E Kelly M Anderson SM Manning R. The OTX2 gene induces tumor growth and triggers leptomeningeal metastasis by regulating the mTORC2 signaling pathway in group 3 medulloblastomas. Int J Mol Sci. (2024) 25:1–22. doi: 10.3390/ijms25084416 PMC11050316 38674001

[106] Peng Z Lv X Sun H Zhao L Huang S. 3D tumor cultures for drug resistance and screening development in clinical applications. Mol Cancer. (2025) 24(1):93. doi: 10.1186/s12943-025-02281-2 40119343 PMC11927140

[107] Wu Z Liu R Shao N Zhao Y. Developing 3D bioprinting for organs-on-chips. Lab Chip. (2025) 25:1081–96. doi: 10.1039/d4lc00769g 39775492

[108] Kalla J Pfneissl J Mair T Tran L Egger G. A systematic review on the culture methods and applications of 3D tumoroids for cancer research and personalized medicine. Cell Oncol. (2024) 48:1–26. doi: 10.1007/s13402-024-00960-8 PMC11850459 38806997

[109] Tao JY Zhu J Gao YQ Jiang M Yin H. Narrative review of 3D bioprinting for the construction of *in vitro* tumor models: present and prospects. Trans Cancer Res. (2025) 14:1479–91. doi: 10.21037/tcr-2025-128 PMC11912033 40104735

[110] Roper SJ Coyle B. Establishing an *in vitro* 3D spheroid model to study medulloblastoma drug response and tumor dissemination. Curr Protoc. (2022) 2:1–29. doi: 10.1002/cpz1.357 35080813

[111] Riedel NC de Faria FW Alfert A Bruder JM Kerl K. Three-dimensional cell culture systems in pediatric and adult brain tumor precision medicine. Cancers (Basel). (2022) 14:5972. doi: 10.3390/cancers14235972 36497454 PMC9738956

[112] Barbosa MAG Xavier CPR Pereira RF Petrikaitė V Vasconcelos MH. 3D cell culture models as recapitulators of the tumor microenvironment for the screening of anti-cancer drugs. Cancers. (2022) 14:1–30. doi: 10.3390/cancers14010190 PMC8749977 35008353

[113] Linke F Aldighieri M Lourdusamy A Grabowska AM Stolnik S Kerr ID. 3D hydrogels reveal medulloblastoma subgroup differences and identify extracellular matrix subtypes that predict patient outcome. J Pathol. (2021) 253:326–38. doi: 10.1002/path.5591 PMC7986745 33206391

[114] Duong Nguyen TT Tanoli Z Hassan S Özcan UO Caroli J Kooistra AJ. PGxDB: an interactive web-platform for pharmacogenomics research. Nucleic Acids Res. (2025) 53:D1486–97. doi: 10.1093/nar/gkae1127 PMC11701576 39565203

[115] Pei Y Moore CE Wang J Tewari AK Eroshkin A Cho YJ. An animal model of MYC-driven medulloblastoma. Cancer Cell. (2012) 21:155–67. doi: 10.1016/j.ccr.2011.12.021 PMC3285431 22340590

[116] Brabetz S Leary SES Gröbner SN Nakamoto MW Şeker-Cin H Girard EJ. A biobank of patient-derived pediatric brain tumor models. Nat Med. (2018) 24:1752–61. doi: 10.1038/s41591-018-0207-3 30349086

[117] Vinci M Burford A Molinari V Kessler K Popov S Clarke M. Functional diversity and cooperativity between subclonal populations of pediatric glioblastoma and diffuse intrinsic pontine glioma cells. Nat Med. (2018) 24:1204–15. doi: 10.1038/s41591-018-0086-7 PMC6086334 29967352

[118] Tang M Rich JN Chen S. Biomaterials and 3D bioprinting strategies to model glioblastoma and the blood-brain barrier. Adv Mater. (2021) 33:e2004776. doi: 10.1002/adma.202004776 33326131 PMC7854518

[119] Branco F Cunha J Mendes M Sousa JJ Vitorino C. 3D bioprinting models for glioblastoma: from scaffold design to therapeutic application. Adv Mater. (2025) 37:e2501994. doi: 10.1002/adma.202501994 40116532

[120] Li Z Langhans SA. *In vivo* and ex vivo pediatric brain tumor models: an overview. Front Oncol. (2021) 11:620831. doi: 10.3389/fonc.2021.620831 33869004 PMC8047472

[121] Blionas A Giakoumettis D Klonou A Neromyliotis E Karydakis P Themistocleous MS. Paediatric gliomas: diagnosis, molecular biology and management. Ann Trans Med. (2018) 6:251–1. doi: 10.21037/atm.2018.05.11 PMC6046297 30069453

[122] Takahashi M Matsuo S Inoue K Tamura K Irie K Kodama Y. Development of an early induction model of medulloblastoma in Ptch1 heterozygous mice initiated with N-ethyl-N-nitrosourea. Cancer Sci. (2012) 103:2051–5. doi: 10.1111/cas.12006 PMC7659221 22937732

[123] Behesti H Bhagat H Dubuc AM Taylor MD Marino S. Bmi1 overexpression in the cerebellar granule cell lineage of mice affects cell proliferation and survival without initiating medulloblastoma formation. DMM Dis Models Mech. (2013) 6:49–63. doi: 10.1242/dmm.009506 PMC3529338 23065639

[124] Kawauchi D Ogg RJ Liu L Shih DJH Finkelstein D Murphy BL. Novel MYC-driven medulloblastoma models from multiple embryonic cerebellar cells. Oncogene. (2017) 36:5231–42. doi: 10.1038/onc.2017.110 PMC5605674 28504719

[125] Mann B Artz N Darawsheh R Kram DE Hingtgen S Satterlee AB. Opportunities and challenges for patient-derived models of brain tumors in functional precision medicine. NPJ Precis Oncol. (2025) 9. doi: 10.1038/s41698-025-00832-w PMC11828933 39953052

[126] Liu Y Wu W Cai C Zhang H Shen H Han Y. Patient-derived xenograft models in cancer therapy: technologies and applications. Signal Transduction Targeted Ther. (2023) 8:160. doi: 10.1038/s41392-023-01419-2 PMC10097874 37045827

[127] Smith KS Xu K Mercer KS Boop F Klimo P DeCupyere M. Patient-derived orthotopic xenografts of pediatric brain tumors: a St. Jude resource. Acta Neuropathologica. (2020) 140:209–25. doi: 10.1007/s00401-020-02171-5 PMC7360541 32519082

[128] Qi L Baxter P Kogiso M Zhang H Braun FK Lindsay H. Direct implantation of patient brain tumor cells into matching locations in mouse brains for patient-derived orthotopic xenograft model development. Cancers. (2024) 16. doi: 10.3390/cancers16091716 PMC11083000 38730671

[129] van Bree N Oppelt AS Lindström S Zhou L Boutin L Coyle B. Development of an orthotopic medulloblastoma zebrafish model for rapid drug testing. Neuro Oncol. (2025) 27:779–94. doi: 10.1093/neuonc/noae210 PMC11889719 39383211

[130] Tsea I Olsen TK Polychronopoulos PA Tümmler C Sykes DB Baryawno N. DHODH inhibition suppresses MYC and inhibits the growth of medulloblastoma in a novel *in vivo* zebrafish model. Cancers. (2024) 16:1–17. doi: 10.3390/cancers16244162 PMC11674817 39766063

[131] Lin CY Erkek S Tong Y Yin L Federation AJ Zapatka M. Active medulloblastoma enhancers reveal subgroup-specific cellular origins. Nature. (2016) 530:57–62. doi: 10.1038/nature16546 26814967 PMC5168934

[132] Casey MJ Chan PP Li Q Zu JF Jette CA Kohler M. A simple and scalable zebrafish model of Sonic hedgehog medulloblastoma. Cell Rep. (2024) 43:114559. doi: 10.1016/j.celrep.2024.114559 39078737 PMC11404834

[133] Shim J Choi JH Park MH Kim H Kim JH Kim SY. Development of zebrafish medulloblastoma-like PNET model by TALEN-mediated somatic gene inactivation. Oncotarget. (2017) 8:55280–97. doi: 10.18632/oncotarget.19424 PMC5589658 28903419

[134] Gatzweiler C Ridinger J Ayhan S Najafi S Peterziel H Witt O. Evaluation of antitumor and on-target activity of HDAC inhibitors with the zebrafish embryo xenograft model. Methods Mol Biol (Clifton NJ). (2023) 2589:75–85. doi: 10.1007/978-1-0716-2788-4_5 36255618

[135] Amaya-Uribe L Rojas M Azizi G Anaya JM Gershwin ME. Primary immunodeficiency and autoimmunity: A comprehensive review. J Autoimmun. (2019) 99:52–72. doi: 10.1016/j.jaut.2019.01.011 30795880

[136] Takaba H Takayanagi H. The mechanisms of T cell selection in the thymus. Trends Immunol. (2017) 38:805–16. doi: 10.1016/j.it.2017.07.010 28830733

[137] Gupta S Louis AG. Tolerance and autoimmunity in primary immunodeficiency disease: A comprehensive review. Clin Rev Allergy Immunol. (2013) 45:162–9. doi: 10.1007/s12016-012-8345-8 23296947

[138] Vittayawacharin P Kongtim P Ciurea SO. Future directions in haploidentical hematopoietic stem cell transplantation. Hematol (United Kingdom). (2024) 29:1–8. doi: 10.1080/16078454.2024.2366718 PMC12577207 38889342

[139] Dhuyser A Aarnink A Pérès M Jayaraman J Nemat-Gorgani N Rubio MT. KIR in allogeneic hematopoietic stem cell transplantation: need for a unified paradigm for donor selection. Front Immunol. (2022) 13:821533. doi: 10.3389/fimmu.2022.821533 35242134 PMC8886110

[140] Sivori S Carlomagno S Pesce S Moretta A Vitale M Marcenaro E. TLR/NCR/KIR: Which one to use and when? Front Immunol. (2014) 5:105. doi: 10.3389/fimmu.2014.00105 24678311 PMC3958761

[141] Lanier LL. NK cell receptors. Annu Rev Immunol. (1998) 16:359–93. doi: 10.1146/annurev.immunol.16.1.359 9597134

[142] Poggi A Zocchi MR. Natural killer cells and immune-checkpoint inhibitor therapy: Current knowledge and new challenges. Mol Ther Oncolytics. (2022) 24:26–42. doi: 10.1016/j.omto.2021.11.016 34977340 PMC8693432

[143] Schakelaar MY Monnikhof M Crnko S Pijnappel EW Meeldijk J ten Broeke T. Cellular immunotherapy for medulloblastoma. Neuro-Oncology. (2023) 25:617–27. doi: 10.1093/neuonc/noac236 PMC10076947 36219688

[144] Kramer K Pandit-Taskar N Humm JL Zanzonico PB Haque S Dunkel IJ. A phase II study of radioimmunotherapy with intraventricular ^131^ I-3F8 for medulloblastoma. Pediatr Blood Cancer. (2018) 65:10.1002/pbc.26754. doi: 10.1002/pbc.26754 PMC6692907 28940863

[145] Ahmed M Cheng M Zhao Q Goldgur Y Cheal SM Guo HF. Humanized affinity-matured monoclonal antibody 8H9 has potent antitumor activity and binds to FG loop of tumor antigen B7-H3. J Biol Chem. (2015) 290:30018–29. doi: 10.1074/jbc.M115.679852 PMC4705981 26487718

[146] Loo D Alderson RF Chen FZ Huang L Zhang W Gorlatov S. Development of an Fc-enhanced anti-B7-H3 monoclonal antibody with potent antitumor activity. Clin Cancer Res. (2012) 18:3834–45. doi: 10.1158/1078-0432.CCR-12-0715 22615450

[147] Yamato I Sho M Nomi T Akahori T Shimada K Hotta K. Clinical importance of B7-H3 expression in human pancreatic cancer. Br J Cancer. (2009) 101:1709–16. doi: 10.1038/sj.bjc.6605375 PMC2778545 19844235

[148] Mews EA Beckmann P Patchava M Wang Y Largaespada DA Wagner CR. Multivalent, bispecific αb7-H3-αCD3 chemically self-assembled nanorings direct potent T cell responses against medulloblastoma. ACS Nano. (2022) 16:12185–201. doi: 10.1021/acsnano.2c02850 PMC9885520 35876221

[149] Liu J Yang S Cao B Zhou G Zhang F Wang Y. Targeting B7-H3 via chimeric antigen receptor T cells and bispecific killer cell engagers augments antitumor response of cytotoxic lymphocytes. J Hematol Oncol. (2021) 14:1–18. doi: 10.1186/s13045-020-01024-8 33514401 PMC7844995

[150] You G Lee Y Kang YW Park HW Park K Kim H. B7-H3×4-1BB bispecific antibody augments antitumor immunity by enhancing terminally differentiated CD8+ tumor-infiltrating lymphocytes. Sci Adv. (2021) 7:1–15. doi: 10.1126/sciadv.aax3160 PMC7810375 33523913

[151] Scribner JA Brown JG Son T Chiechi M Li P Sharma S. Preclinical development of MGC018, a duocarmycin-based antibody–drug conjugate targeting B7-H3 for solid cancer. Mol Cancer Ther. (2020) 19:2235–44. doi: 10.1158/1535-7163.MCT-20-0116 32967924

[152] Yamato M Hasegawa J Maejima T Hattori C Kumagai K Watanabe A. DS-7300a, a DNA topoisomerase I inhibitor, DXd-based antibody-drug conjugate targeting B7-H3, exerts potent antitumor activities in preclinical models. Mol Cancer Ther. (2022) 21:635–46. doi: 10.1158/1535-7163.MCT-21-0554 PMC9377751 35149548

[153] Seaman S Zhu Z Saha S Zhang XM Yang MY Hilton MB. Eradication of tumors through simultaneous ablation of CD276/B7-H3-positive tumor cells and tumor vasculature. Cancer Cell. (2017) 31:501–515.e8. doi: 10.1016/j.ccell.2017.03.005 28399408 PMC5458750

[154] Tang M Chen C Wang G Wang Y Zhang Z Li H. Evaluation of B7-H3 targeted immunotherapy in a 3D organoid model of craniopharyngioma. Biomolecules. (2022) 12:1–11. doi: 10.3390/biom12121744 PMC9775874 36551172

[155] Jaeger M Nör C De Farias CB Abujamra AL Schwartsmann G Brunetto AL. Anti-EGFR therapy combined with neuromedin B receptor blockade induces the death of DAOY medulloblastoma cells. Child’s Nervous System. (2013) 29:2145–50. doi: 10.1007/s00381-013-2290-6 24092425

[156] Coy S Lee JS Chan SJ Woo T Jones J Alexandrescu S. Systematic characterization of antibody–drug conjugate targets in central nervous system tumors. Neuro-Oncology. (2024) 26:458–72. doi: 10.1093/neuonc/noad205 PMC10912007 37870091

[157] Chandramohan V Bao X Kato Kaneko M Kato Y Keir ST Szafranski SE. Recombinant anti-podoplanin (NZ-1) immunotoxin for the treatment of Malignant brain tumors. Int J Cancer. (2013) 132:2339–48. doi: 10.1002/ijc.27919 PMC3809846 23115013

[158] Gholamin S Mitra SS Feroze AH Liu J Kahn SA Zhang M. Disrupting the CD47-SIRPα anti-phagocytic axis by a humanized anti-CD47 antibody is an efficacious treatment for Malignant pediatric brain tumors. Sci Transl Med. (2017) 9:eaaf2968. doi: 10.1126/scitranslmed.aaf2968 28298418

[159] Levy AS Krailo M Chi S Villaluna D Springer L Williams-Hughes C. Temozolomide with irinotecan versus temozolomide, irinotecan plus bevacizumab for recurrent medulloblastoma of childhood: Report of a COG randomized Phase II screening trial. Pediatr Blood Cancer. (2021) 68:e29031. doi: 10.1002/pbc.29031 33844469 PMC8764558

[160] Gauthier M Pierson J Moulin D Mouginot M Bourguignon V Rhalloussi W. Deciphering Natural Killer Cell Cytotoxicity Against Medulloblastoma *in vitro* and *in vivo*: Implications for Immunotherapy. ImmunoTargets Ther. (2024) 13:319–33. doi: 10.2147/ITT.S458278 PMC11214763 38948503

[161] Castriconi R Dondero A Negri F Bellora F Nozza P Carnemolla B. Both CD133+ and CD133- medulloblastoma cell lines express ligands for triggering NK receptors and are susceptible to NK-mediated cytotoxicity. Eur J Immunol. (2007) 37:3190–6. doi: 10.1002/eji.200737546 17918205

[162] Fernández L Portugal R Valentín J Martín R Maxwell H González-Vicent M. *In vitro* natural killer cell immunotherapy for medulloblastoma. Front Oncol. (2013) 3:94. doi: 10.3389/fonc.2013.00094 23626949 PMC3630393

[163] Sehl OC Yang Y Anjier AR Nevozhay D Cheng D Guo K. Preclinical and Clinical-Scale Magnetic Particle Imaging of Natural Killer Cells: *in vitro* and ex vivo Demonstration of Cellular Sensitivity, Resolution, and Quantification. Mol Imaging Biol. (2024) 27:78–88. doi: 10.1007/s11307-024-01969-z 39653984 PMC12257978

[164] Raso A Negri F Gregorio A Nozza P Mascelli S De Marco P. Successful isolation and long-term establishment of a cell line with stem cell-like features from an anaplastic medulloblastoma. Neuropathol Appl Neurobiol. (2008) 34:306–15. doi: 10.1111/j.1365-2990.2007.00896.x 17995922

[165] Raffaghello L Nozza P Morandi F Camoriano M Wang X Garrè ML. Expression and functional analysis of human leukocyte antigen class I antigen-processing machinery in medulloblastoma. Cancer Res. (2007) 67:5471–8. doi: 10.1158/0008-5472.CAN-06-4735 17545629

[166] Smith C Santi M Rajan B Rushing EJ Choi MR Rood BR. A novel role of HLA class I in the pathology of medulloblastoma. J Trans Med. (2009) 7:1–13. doi: 10.1186/1479-5876-7-59 PMC2714836 19594892

[167] Cifaldi L Romania P Falco M Lorenzi S Meazza R Petrini S. ERAP1 regulates natural killer cell function by controlling the engagement of inhibitory receptors. Cancer Res. (2015) 75:824–34. doi: 10.1158/0008-5472.CAN-14-1643 25592150

[168] Kennis BA Michel KA Brugmann WB Laureano A Tao RH Somanchi SS. Correction to: Monitoring of intracerebellarly-administered natural killer cells with fluorine-19 MRI (Journal of Neuro-Oncology, (2019), 142, 3, (395-407), 10.1007/s11060-019-03091-5). J Neuro-Oncology. (2019) 142:409. doi: 10.1007/s11060-019-03162-7 PMC11492566 30788681

[169] Liang KH Chang CC Wu KS Yu AL Sung SY Lee YY. Notch signaling and natural killer cell infiltration in tumor tissues underlie medulloblastoma prognosis. Sci Rep. (2021) 11:1–12. doi: 10.1038/s41598-021-02651-y 34857809 PMC8639846

[170] Lago C Gianesello M Santomaso L Leva G Ballabio C Anderle M. Medulloblastoma and high-grade glioma organoids for drug screening, lineage tracing, co-culture and *in vivo* assay. Nat Protoc. (2023) 18:2143–80. doi: 10.1038/s41596-023-00839-2 37248391

[171] Tao R Han K Wu SC Friske JD Roussel MF Northcott PA. Arrested development: the dysfunctional life history of medulloblastoma. Genes Dev. (2024) 39:4–17. doi: 10.1101/gad.351936.124 PMC11789489 39231614

[172] Muñoz Perez N Pensabene JM Galbo PM Jr Sadeghipour N Xiu J Moziak K. VISTA emerges as a promising target against immune evasion mechanisms in medulloblastoma. Cancers (Basel). (2024) 16:2629. doi: 10.3390/cancers16152629 39123357 PMC11312086

[173] Liu D Song L Brawley VS Robison N Wei J Gao X. Medulloblastoma expresses CD1d and can be targeted for immunotherapy with NKT cells. Clin Immunol. (2013) 149:55–64. doi: 10.1016/j.clim.2013.06.005 23891738 PMC3809126

[174] Goldsmith BSLHCJJLTHSAMKK. Abstract 1790: Exploring the efficacy of allogeneic gamma delta T cell adoptive cell therapy against the pediatric brain tumor medulloblastoma. Advancing Front Cancer Sci Med. (2023), 1970.

[175] Boutin L Liu M Déchanet Merville J Bedoya-Reina O Wilhelm MT. EphA2 and phosphoantigen-mediated selective killing of medulloblastoma by γδT cells preserves neuronal and stem cell integrity. Oncoimmunology. (2025) 14:2485535. doi: 10.1080/2162402X.2025.2485535 40190167 PMC11980450

[176] Benelli R Costa D Salvini L Tardito S Tosetti F Villa F. Targeting of colorectal cancer organoids with zoledronic acid conjugated to the anti-EGFR antibody cetuximab. J Immunother Cancer. (2022) 10:e005660. doi: 10.1136/jitc-2022-005660 36543375 PMC9772689

[177] Lowry BS Jonus HC Lee J Spencer TH Kenney AM Goldsmith K. (2023). Exploring the efficacy of allogeneic gamma delta T cell adoptive cell therapy against the pediatric brain tumor medulloblastoma, in: Proceedings of the American Association for Cancer Research Annual Meeting 2023; Part 1 (Regular and Invited Abstracts), Orlando, FL. Philadelphia (PA, 2023 Apr 14-19, Vol. 83. p. Abstract nr 1790. AACR; Cancer Res.

[178] Shariati A Khezrpour A Shariati F Afkhami H Yarahmadi A Alavimanesh S. DNA vaccines as promising immuno-therapeutics against cancer: a new insight. Front Immunol. (2024) 15:1498431. doi: 10.3389/fimmu.2024.1498431 39872522 PMC11769820

[179] Jayathilaka B Mian F Franchini F Au-Yeung G IJzerman M. Cancer and treatment specific incidence rates of immune-related adverse events induced by immune checkpoint inhibitors: a systematic review. Br J Cancer. (2024) 32:51–7. doi: 10.1038/s41416-024-02887-1 PMC11723908 39489880

[180] Badani A Ozair A Khasraw M Woodworth GF Tiwari P Ahluwalia MS. Immune checkpoint inhibitors for glioblastoma: emerging science, clinical advances, and future directions. J Neuro-Oncology. (2024) 171:531–47. doi: 10.1007/s11060-024-04881-2 PMC12188872 39570554

[181] Huang Q Xu L Ye L. Functional subsets of tumor-specific CD8+ T cells in draining lymph nodes and tumor microenvironment. Curr Opin Immunol. (2025) 93:102506. doi: 10.1016/j.coi.2024.102506 39591663

[182] Ouyang Y Liu W Zhang N Yang X Li J Long S. Prognostic significance of programmed cell death-ligand 1 expression on circulating tumor cells in various cancers: A systematic review and meta-analysis. Cancer Med. (2021) 10:7021–39. doi: 10.1002/cam4.4236 PMC8525108 34423578

[183] Hoyek C Zheng-lin B Bauernfeind JJ Al-ahmad OH. Overcoming financial and access barriers in global cancer care with low-dose immunotherapy: A systematic review. JCO Glob Oncol. (2025) 11:e2400409. doi: 10.1200/GO-24-00409 40127379

[184] Yang C Trivedi V Dyson K Gu T Candelario KM Yegorov O. Identification of tumor rejection antigens and the immunologic landscape of medulloblastoma. Genome Med. (2024) 16:1–19. doi: 10.1186/s13073-024-01363-y 39160595 PMC11331754

[185] Zinkernagel RM Bachmann MF Kündig TM Oehen S Pirchet H Hengartner H. On immunological memory. Annu Rev Immunol. (1996) 14:333–67. doi: 10.1146/annurev.immunol.14.1.333 8717518

[186] Trivedi V Yang C Klippel K Yegorov O von Roemeling C Hoang-Minh L. mRNA-based precision targeting of neoantigens and tumor-associated antigens in Malignant brain tumors. Genome Med. (2024) 16:1–19. doi: 10.1186/s13073-024-01281-z 38268001 PMC10809449

[187] Blaeschke F Paul MC Schuhmann MU Rabsteyn A Schroeder C Casadei N. Low mutational load in pediatric medulloblastoma still translates into neoantigens as targets for specific T-cell immunotherapy. Cytotherapy. (2019) 21:973–86. doi: 10.1016/j.jcyt.2019.06.009 31351799

[188] Paret C Lehmann N Bender H Sprang M Sommer CJ Cana D. Identification of an immunogenic medulloblastoma-specific fusion 1420 involving EPC2 and *GULP1* . Cancers (Basel). (2021) 13:5838. doi: 10.3390/cancers13225838 34830991 PMC8616194

[189] Rivero-Hinojosa S Grant M Panigrahi A Zhang H Caisova V Bollard CM. Proteogenomic discovery of neoantigens facilitates personalized multi-antigen targeted T cell immunotherapy for brain tumors. Nat Commun. (2021) 12:1–15. doi: 10.1038/s41467-021-26936-y 34795224 PMC8602676

[190] Chlapek P Zitterbart K Kren L Filipova L Sterba J Veselska R. Uniformity under *in vitro* conditions: Changes in the phenotype of cancer cell lines derived from different medulloblastoma subgroups. PloS One. (2017) 12:1–14. doi: 10.1371/journal.pone.0172552 PMC5322931 28231263

[191] Liang L Aiken C McClelland R Morrison LC Tatari N Remke M. Characterization of novel biomarkers in selecting for subtype specific medulloblastoma phenotypes. Oncotarget. (2015) 6:38881–900. doi: 10.18632/oncotarget.6195 PMC4770744 26497209

[192] Gronseth E Gupta A Koceja C Kumar S Kutty RG Rarick K. Astrocytes influence medulloblastoma phenotypes and CD133 surface expression. PloS One. (2020) 15:1–17. doi: 10.1371/journal.pone.0235852 PMC7337293 32628717

[193] Capdeville C Russo L Penton D Migliavacca J Zecevic M Gries A. Spatial proteomics finds CD155 and Endophilin-A1 as mediators of growth and invasion in medulloblastoma. Life Sci Alliance. (2022) 5:1–18. doi: 10.26508/LSA.202201380 PMC8926928 35296518

[194] Friedman GK Moore BP Nan L Kelly VM Etminan T Langford CP. Pediatric medulloblastoma xenografts including molecular subgroup 3 and CD133+ and CD15+ cells are sensitive to killing by oncolytic herpes simplex viruses. Neuro-Oncology. (2016) 18:227–35. doi: 10.1093/neuonc/nov123 PMC4724175 26188016

[195] Sandén E Dyberg C Krona C Visse E Carén H Northcott PA. Aberrant immunostaining pattern of the CD24 glycoprotein in clinical samples and experimental models of pediatric medulloblastomas. J Neuro-Oncology. (2015) 123:1–13. doi: 10.1007/s11060-015-1758-5 25820321

[196] Ciccone R Quintarelli C Camera A Pezzella M Caruso S Manni S. GD2-targeting CAR T-cell therapy for patients with GD2+ Medulloblastoma. Clin Cancer Res. (2024) 30:2545–57. doi: 10.1158/1078-0432.CCR-23-1880 PMC11145172 38551501

[197] Paret C Ustjanzew A Ersali S Seidmann L Jennemann R Ziegler N. GD2 expression in medulloblastoma and neuroblastoma for personalized immunotherapy: A matter of subtype. Cancers. (2022) 14:1–22. doi: 10.3390/cancers14246051 PMC9775636 36551537

[198] Rahmann EP Gilbertson RJ. Multiomic medulloblastomas. Cancer Cell. (2018) 34:351–3. doi: 10.1016/j.ccell.2018.08.010 30205039

[199] Gilbertson RJ. Medulloblastoma: signalling a change in treatment. Lancet Oncol. (2004) 5:209–18. doi: 10.1016/S1470-2045(04)01424-X 15050952

[200] Xiao T Xiao Y Wang W Tang YY Xiao Z Su M. Targeting ephA2 in cancer. J Hematol Oncol. (2020) 13:1–17. doi: 10.1186/s13045-020-00944-9 32811512 PMC7433191

[201] Meurer RT Martins DT Hilbig A Ribeiro MDC Roehe AV Barbosa-Coutinho LM. Immunohistochemical expression of markers KI-67, NeuN, synaptophysin, p53 and HER2 in medulloblastoma and its correlation with clinicopathological parameters. Arquivos Neuro-Psiquiatria. (2008) 66:385–90. doi: 10.1590/S0004-282X2008000300020 18641877

[202] Patereli A Alexiou GA Stefanaki K Moschovi M Doussis-Anagnostopoulou I Prodromou N. Expression of epidermal growth factor receptor and HER-2 in pediatric embryonal brain tumors. Pediatr Neurosurg. (2010) 46:188–92. doi: 10.1159/000316640 20962551

[203] Majzner RG Theruvath JL Nellan A Heitzeneder S Cui Y Mount CW. CAR T cells targeting B7-H3, a pan-cancer antigen, demonstrate potent preclinical activity against pediatric solid tumors and brain tumors. Clin Cancer Res. (2019) 25:2560–74. doi: 10.1158/1078-0432.CCR-18-0432 PMC8456711 30655315

[204] Tanzhu G Chen L Ning J Xue W Wang C Xiao G. Metastatic brain tumors: from development to cutting-edge treatment. MedComm. (2025) 6:1–73. doi: 10.1002/mco2.70020 PMC11661909 39712454

[205] Mortezaee K. B7-H3 immunoregulatory roles in cancer. Biomedicine Pharmacotherapy. (2023) 163:114890. doi: 10.1016/j.biopha.2023.114890 37196544

[206] Guo X Chang M Wang Y Xing B Ma W. B7-H3 in brain Malignancies: immunology and immunotherapy. Int J Biol Sci. (2023) 19:3762–80. doi: 10.7150/ijbs.85813 PMC10411461 37564196

[207] Varlet P Bouffet E Casanova M Giangaspero F Antonelli M Hargrave D. Comprehensive analysis of the ErbB receptor family in pediatric nervous system tumors and rhabdomyosarcoma. Pediatr Blood Cancer. (2022) 69:1–11. doi: 10.1002/pbc.29316 34546642

[208] Ni H Reitman ZJ Zou W Akhtar MN Paul R Huang M. FLASH radiation reprograms lipid metabolism and macrophage immunity and sensitizes medulloblastoma to CAR-T cell therapy. Nat Cancer. (2025) 6:460–73. doi: 10.1038/s43018-025-00905-6 PMC12244404 39910249

[209] Zhou D Zhu X Xiao Y. Advances in CAR-T therapy for central nervous system tumors. biomark Res. (2024) 12:1–36. doi: 10.1186/s40364-024-00679-6 39506843 PMC11539471

[210] Minguet S Maus MV Schamel WW. From TCR fundamental research to innovative chimeric antigen receptor design. Nat Rev Immunol. (2025) 25:212–24. doi: 10.1038/s41577-024-01093-7 39433885

[211] Bangolo A Amoozgar B Mansour C Zhang L Gill S Ip A. Comprehensive review of early and late toxicities in CAR T-cell therapy and bispecific antibody treatments for hematologic Malignancies. Cancers. (2025) 17:1–21. doi: 10.3390/cancers17020282 PMC11764151 39858064

[212] Das A McDonald D Lowe S Bredlau AL Vanek K Patel SJ. Immunological low-dose radiation modulates the pediatric medulloblastoma antigens and enhances antibody-dependent cellular cytotoxicity. Child’s Nervous System. (2017) 33:429–36. doi: 10.1007/s00381-016-3305-x 27942918

[213] Kong R Liu B Wang H Lu T Zhou X. CAR-NK cell therapy: latest updates from the 2024 ASH annual meeting. J Hematol Oncol. (2025) 18:0–3. doi: 10.1186/s13045-025-01677-3 PMC11872314 40025557

[214] Dehghan F Metanat Y Askarizadeh M Ahmadi E Moradi V. Novel gene manipulation approaches to unlock the existing bottlenecks of CAR-NK cell therapy. Front Cell Dev Biol. (2024) 12:1511931. doi: 10.3389/fcell.2024.1511931 40007761 PMC11850336

[215] Sloas C Gill S Klichinsky M. Engineered CAR-macrophages as adoptive immunotherapies for solid tumors. Front Immunol. (2021) 12:783305. doi: 10.3389/fimmu.2021.783305 34899748 PMC8652144

[216] Sampson JH Gunn MD Fecci PE Ashley DM. Brain immunology and immunotherapy in brain tumours. Nat Rev Cancer. (2020) 20:12–25. doi: 10.1038/s41568-019-0224-7 31806885 PMC7327710

[217] Zhang X Zhang H Lan H Wu J Xiao Y. CAR-T cell therapy in multiple myeloma: Current limitations and potential strategies. Front Immunol. (2023) 14:1101495. doi: 10.3389/fimmu.2023.1101495 36891310 PMC9986336

[218] Capolla S Rasool M Toffoli G Dal Bo M. CAR-T cell manufacturing for hematological and solid tumors: from the preclinical to clinical point of view. Cancer Med. (2025) 14:e70726. doi: 10.1002/cam4.70726 40013750 PMC11866474

[219] Canciani G Fabozzi F Pinacchio C Ceccarelli M Del Bufalo F. Developing CAR T-cell therapies for pediatric solid tumors. Paediatric Drugs. (2024) 27:5–18. doi: 10.1007/s40272-024-00653-7 39382819

[220] Lin YJ Mashouf LA Lim M. CAR T cell therapy in primary brain tumors: current investigations and the future. Front Immunol. (2022) 13:817296. doi: 10.3389/fimmu.2022.817296 35265074 PMC8899093

[221] Donovan LK Delaidelli A Joseph SK Bielamowicz K Fousek K Holgado BL. Locoregional delivery of CAR T cells to the cerebrospinal fluid for treatment of metastatic medulloblastoma and ependymoma. Nat Med. (2020) 26:720–31. doi: 10.1038/s41591-020-0827-2 PMC8815773 32341580

[222] Contessa JN Hamstra DA. Revoking the privilege: targeting HER2 in the central nervous system. Mol Pharmacol. (2008) 73:271–3. doi: 10.1124/mol.107.042986 17981994

[223] Hashimoto Y Penas-Prado M Zhou S Wei J Khatua S Hodges TR. Rethinking medulloblastoma from a targeted therapeutics perspective. J Neuro-Oncology. (2018) 139:713–20. doi: 10.1007/s11060-018-2917-2 PMC6132970 29869738

[224] Meco D Servidei T Zannoni GF Martinelli E Prisco MG de Waure C. Dual inhibitor AEE788 reduces tumor growth in preclinical models of medulloblastoma. Trans Oncol. (2010) 3:326–35. doi: 10.1593/tlo.10163 PMC2935636 20885895

[225] Gilbertson RJ Perry RH Kelly PJ Pearson AD Lunec J. Prognostic significance of HER2 and HER4 coexpression in childhood medulloblastoma. Cancer Res. (1997) 57:3272–80. 9242460

[226] Hudelist G Singer CF Manavi M Pischinger K Kubista E Czerwenka K. Co-expression of ErbB-family members in human breast cancer: Her-2/neu is the preferred dimerization candidate in nodal-positive tumors. Breast Cancer Res Treat. (2003) 80:353–61. doi: 10.1023/A:1024929522376 14503808

[227] Nellan A Rota C Majzner R Lester-McCully CM Griesinger AM Mulcahy Levy JM. Durable regression of Medulloblastoma after regional and intravenous delivery of anti-HER2 chimeric antigen receptor T cells. J ImmunoTherapy Cancer. (2018) 6:1–14. doi: 10.1186/s40425-018-0340-z PMC5925833 29712574

[228] Ahmed N Ratnayake M Savoldo B Perlaky L Dotti G Wels WS. Regression of experimental medulloblastoma following transfer of HER2-specific T cells. Cancer Res. (2007) 67:5957–64. doi: 10.1158/0008-5472.CAN-06-4309 17575166

[229] Waage IS Vreim I Torp SH. C-erbB2/HER2 in human gliomas, medulloblastomas, and meningiomas: A minireview. Int J Surg Pathol. (2013) 21:573–82. doi: 10.1177/1066896913492196 23842006

[230] Meco D Servidei T Riccardi A Ferlini C Cusano G Zannoni GF. Antitumor effect in medulloblastoma cells by gefitinib: Ectopic HER2 overexpression enhances gefitinib effects. vivo. Neuro-Oncology. (2009) 11:250–9. doi: 10.1215/15228517-2008-095 PMC2718969 19033425

[231] Bernardi RJ Lowery AR Thompson PA Blaney SM West JL. Immunonanoshells for targeted photothermal ablation in medulloblastoma and glioma: An *in vitro* evaluation using human cell lines. J Neuro-Oncology. (2008) 86:165–72. doi: 10.1007/s11060-007-9467-3 17805488

[232] Rickert CH Paulus W. Prognosis-related histomorphological and immunohistochemical markers in central nervous system tumors of childhood and adolescence. Acta Neuropathol. (2005) 109:69–92. doi: 10.1007/s00401-004-0959-3 15647946

[233] Bodey B Kaiser HE Siegel SE. Epidermal growth factor receptor (EGFR) expression in childhood brain tumors. In Vivo. (2005) 19:931–41. 16097449

[234] Vitanza NA Johnson AJ Wilson AL Brown C Yokoyama JK Künkele A. Locoregional infusion of HER2-specific CAR T cells in children and young adults with recurrent or refractory CNS tumors: an interim analysis. Nat Med. (2021) 27:1544–52. doi: 10.1038/s41591-021-01404-8 34253928

[235] Kawakami M Kawakami K Takahashi S Abe M Puri RK. Analysis of interleukin-13 receptor α2 expression in human pediatric brain tumors. Cancer. (2004) 101:1036–42. doi: 10.1002/cncr.20470 15329913

[236] Jaén M Martín-Regalado Á Bartolomé RA Robles J Casal JI. Interleukin 13 receptor alpha 2 (IL13Rα2): Expression, signaling pathways and therapeutic applications in cancer. Biochim Biophys Acta - Rev Cancer. (2022) 1877:188802. doi: 10.1016/j.bbcan.2022.188802 36152905

[237] Feustel K Martin J Falchook GS. B7-H3 inhibitors in oncology clinical trials: A review. J Immunotherapy Precis Oncol. (2024) 7:53–66. doi: 10.36401/JIPO-23-18 PMC10846634 38327753

[238] Zhou WT Jin WL. B7-H3/CD276: an emerging cancer immunotherapy. Front Immunol. (2021) 12:701006. doi: 10.3389/fimmu.2021.701006 34349762 PMC8326801

[239] Kontos F Michelakos T Kurokawa T Sadagopan A Schwab JH Ferrone CR. B7-H3: An attractive target for antibody-based immunotherapy. Clin Cancer Res. (2021) 27:1227–35. doi: 10.1158/1078-0432.CCR-20-2584 PMC7925343 33051306

[240] Arafat Hossain M. A comprehensive review of immune checkpoint inhibitors for cancer treatment. Int Immunopharmacol. (2024) 143:113365. doi: 10.1016/j.intimp.2024.113365 39447408

[241] Martínez-Gamboa DA Hans R Moreno-Cortes E Figueroa-Aguirre J Garcia-Robledo JE Vargas-Cely F. CAR T-cell therapy landscape in pediatric, adolescent and young adult oncology – A comprehensive analysis of clinical trials. Crit Rev Oncology/Hematology. (2025) 209:104648. doi: 10.1016/j.critrevonc.2025.104648 39900318

[242] Purvis IJ Avilala J Guda MR Venkataraman S Vibhakar R Tsung AJ. Role of MYC-miR-29-B7-H3 in medulloblastoma growth and angiogenesis. J Clin Med. (2019) 8:1158. doi: 10.3390/jcm8081158 31382461 PMC6723910

[243] Kanchan RK Perumal N Atri P Chirravuri Venkata R Thapa I Klinkebiel DL. MiR-1253 exerts tumor-suppressive effects in medulloblastoma via inhibition of CDK6 and CD276 (B7-H3). Brain Pathol. (2020) 30:732–45. doi: 10.1111/bpa.12829 PMC7383594 32145124

[244] Getu AA Tigabu A Zhou M Lu J Fodstad Ø Tan M. New frontiers in immune checkpoint B7-H3 (CD276) research and drug development. Mol Cancer. (2023) 22:1–15. doi: 10.1186/s12943-023-01751-9 36859240 PMC9979440

[245] Li S Poolen GC van Vliet LC Schipper JG Broekhuizen R Monnikhof M. Pediatric medulloblastoma express immune checkpoint B7-H3. Clin Trans Oncol. (2022) 24:1204–8. doi: 10.1007/s12094-021-02762-y PMC9107433 34988920

[246] Purvis IJ Velpula KK Guda MR Nguyen D Tsung AJ Asuthkar S. B7-h3 in medulloblastoma-derived exosomes; a novel tumorigenic role. Int J Mol Sci. (2020) 21:1–14. doi: 10.3390/ijms21197050 PMC7583814 32992699

[247] Picarda E Ohaegbulam KC Zang X. Molecular pathways: Targeting B7-H3 (CD276) for human cancer immunotherapy. Clin Cancer Res. (2016) 22:3425–31. doi: 10.1158/1078-0432.CCR-15-2428 PMC4947428 27208063

[248] Sun M Richards S Prasad DVR Mai XM Rudensky A Dong C. Characterization of mouse and human B7-H3 genes. J Immunol. (2002) 168:6294–7. doi: 10.4049/jimmunol.168.12.6294 12055244

[249] Liao S Huang J Lupala CS Li X Li X Li N. Identification of the B7-H3 interaction partners using a proximity labeling strategy. Int J Mol Sci. (2025) 26:1731. doi: 10.3390/ijms26041731 40004194 PMC11855656

[250] Cox J Hein MY Luber CA Paron I Nagaraj N Mann M. Accurate proteome-wide label-free quantification by delayed normalization and maximal peptide ratio extraction, termed MaxLFQ. Mol Cell Proteomics. (2014) 13:2513–26. doi: 10.1074/mcp.M113.031591 PMC4159666 24942700

[251] Husain B Ramani SR Chiang E Lehoux I Paduchuri S Arena TA. A platform for extracellular interactome discovery identifies novel functional binding partners for the immune receptors B7-H3/CD276 and PVR/CD155. Mol Cell Proteomics. (2019) 18:2310–23. doi: 10.1074/mcp.TIR119.001433 PMC6823854 31308249

[252] Cao S Peterson SM Müller S Reichelt M Amador CMR Martinez-Martin N. A membrane protein display platform for receptor interactome discovery. Proc Natl Acad Sci United States America. (2021) 118:1–12. doi: 10.1073/pnas.2025451118 PMC8488672 34531301

[253] Ciprut S Berberich A Knoll M Pusch S Hoffmann D Furkel J. Neuro-oncology advances. Neuro-Oncology Advances. (2022) 4:1–10. doi: 10.1093/noajnl/vdac098 PMC9341442 35919070

[254] Hashiguchi M Kobori H Ritprajak P Kamimura Y Kozono H Azuma M. Triggering receptor expressed on myeloid cell-like transcript 2 (TLT-2) is a counter-receptor for B7-H3 and enhances T cell responses. Proc Natl Acad Sci United States America. (2008) 105:10495–500. doi: 10.1073/pnas.0802423105 PMC2492502 18650384

[255] van Bree NFHN Wilhelm M. The tumor microenvironment of medulloblastoma: an intricate multicellular network with therapeutic potential. Cancers. (2022) 14:5009. doi: 10.3390/cancers14205009 36291792 PMC9599673

[256] Thompson EM Keir ST Venkatraman T Lascola C Yeom KW Nixon AB. The role of angiogenesis in Group 3 medulloblastoma pathogenesis and survival. Neuro-Oncology. (2017) 19:1217–27. doi: 10.1093/neuonc/nox033 PMC5570262 28379574

[257] Chen C Cai S Wang G Cao X Yang X Luo X. C-Myc enhances colon cancer cell-mediated angiogenesis through the regulation of HIF-1α. Biochem Biophys Res Commun. (2013) 430:505–11. doi: 10.1016/j.bbrc.2012.12.006 23237807

[258] Dews M Homayouni A Yu D Murphy D Sevignani C Wentzel E. Augmentation of tumor angiogenesis by a Myc-activated microRNA cluster. Nat Genet. (2006) 38:1060–5. doi: 10.1038/ng1855 PMC2669546 16878133

[259] Green AR Aleskandarany MA Agarwal D Elsheikh S Nolan CC Diez-Rodriguez M. MYC functions are specific in biological subtypes of breast cancer and confers resistance to endocrine therapy in luminal tumours. Br J Cancer. (2016) 114:917–28. doi: 10.1038/bjc.2016.46 PMC4984797 26954716

[260] Chen X Yang F Zhang T Wang W Xi W Li Y. MiR-9 promotes tumorigenesis and angiogenesis and is activated by MYC and OCT4 in human glioma. J Exp Clin Cancer Res. (2019) 38:1–16. doi: 10.1186/s13046-019-1078-2 30795814 PMC6385476

[261] Meškytė EM Keskas S Ciribilli Y. Myc as a multifaceted regulator of tumor microenvironment leading to metastasis. Int J Mol Sci. (2020) 21:1–29. doi: 10.3390/ijms21207710 PMC7589112 33081056

[262] Gearhart J Pashos EE Prasad MK. Pluripotency redux–advances in stem-cell research. N Engl J Med. (2007) 357:1469–72. doi: 10.1056/NEJMp078126 17928593

[263] Kumar D Kanchan R Chaturvedi NK. Targeting protein synthesis pathways in MYC-amplified medulloblastoma. Discover Oncol. (2025) 16. doi: 10.1007/s12672-025-01761-7 PMC11711608 39779613

[264] Saker Z Rizk M Bahmad HF Nabha SM. Targeting angiogenic factors for the treatment of medulloblastoma. Curr Treat Options Oncol. (2022) 23:864–86. doi: 10.1007/s11864-022-00981-1 35412196

[265] Huber H Eggert A Janss AJ Wiewrodt R Zhao H Sutton LN. Angiogenic profile of childhood primitive neuroectodermal brain tumours/medulloblastomas. Eur J Cancer. (2001) 37:2064–72. doi: 10.1016/s0959-8049(01)00225-8 11597385

[266] Aguilera D Mazewski C Fangusaro J MacDonald TJ McNall-Knapp RY Hayes LL. Response to bevacizumab, irinotecan, and temozolomide in children with relapsed medulloblastoma: A multi-institutional experience. Child’s Nervous System. (2013) 29:589–96. doi: 10.1007/s00381-012-2013-4 PMC3963487 23296323

[267] Peyrl A Chocholous M Sabel M Lassaletta A Sterba J Leblond P. Sustained survival benefit in recurrent medulloblastoma by a metronomic antiangiogenic regimen: A nonrandomized controlled trial. JAMA Oncol. (2023) 9:1688–95. doi: 10.1001/jamaoncol.2023.4437 PMC10603581 37883081

[268] Olmez I Love S Xiao A Manigat L Randolph P McKenna BD. Targeting the mesenchymal subtype in glioblastoma and other cancers via inhibition of diacylglycerol kinase alpha. Neuro Oncol. (2018) 20:192–202. doi: 10.1093/neuonc/nox119 29048560 PMC5777487

[269] Wang Y Peng D Huang Y Cao Y Li H Zhang X. Podoplanin: Its roles and functions in neurological diseases and brain cancers. Front Pharmacol. (2022) 13:964973. doi: 10.3389/fphar.2022.964973 36176432 PMC9514838

[270] Mashhadiabbas F Chafjiri MM Dashti M Mudasser MA Gholami S. Podoplanin expression in various types of oral dysplasia and oral squamous cell carcinoma. J Taibah Univ Med Sci. (2024) 20:40–51. doi: 10.1016/j.jtumed.2024.11.007 39866370 PMC11761884

[271] Parhar S Kaur H Vashist A Verma S. Role of podoplanin in potentially Malignant disorders and oral squamous cell carcinoma and its correlation with lymphangiogenesis. Indian J Cancer. (2015) 52:617–22. doi: 10.4103/0019-509X.178427 26960495

[272] Kunita A Kashima TG Morishita Y Fukayama M Kato Y Tsuruo T. The platelet aggregation-inducing factor aggrus/podoplanin promotes pulmonary metastasis. Am J Pathol. (2007) 170:1337–47. doi: 10.2353/ajpath.2007.060790 PMC1829466 17392172

[273] Wicki A Lehembre F Wick N Hantusch B Kerjaschki D Christofori G. Tumor invasion in the absence of epithelial-mesenchymal transition: podoplanin-mediated remodeling of the actin cytoskeleton. Cancer Cell. (2006) 9:261–72. doi: 10.1016/j.ccr.2006.03.010 16616332

[274] Kato Y Kaneko MK Kunita A Ito H Kameyama A Ogasawara S. Molecular analysis of the pathophysiological binding of the platelet aggregation-inducing factor podoplanin to the C-type lectin-like receptor CLEC-2. Cancer Sci. (2008) 99:54–61. doi: 10.1111/j.1349-7006.2007.00634.x 17944973 PMC11159596

[275] Astarita JL Acton SE Turley SJ. Podoplanin: emerging functions in development, the immune system, and cancer. Front Immunol. (2012) :283. doi: 10.3389/fimmu.2012.00283 22988448 PMC3439854

[276] Zhang Z Zhang N Yu J Xu W Gao J Lv X. The role of podoplanin in the immune system and inflammation. J Inflammation Res. (2022) 15:3561–72. doi: 10.2147/JIR.S366620 PMC9212786 35747250

[277] Shiina S Ohno M Ohka F Kuramitsu S Yamamichi A Kato A. CAR T cells targeting Podoplanin reduce orthotopic glioblastomas in mouse brains. Cancer Immunol Res. (2016) 4:259–68. doi: 10.1158/2326-6066.CIR-15-0060 26822025

[278] Poggi A Varesano S Zocchi MR. How to hit mesenchymal stromal cells and make the tumor microenvironment immunostimulant rather than immunosuppressive. Front Immunol. (2018) 9:262. doi: 10.3389/fimmu.2018.00262 29515580 PMC5825917

[279] Poggi A Giuliani M. Mesenchymal stromal cells can regulate the immune response in the tumor microenvironment. Vaccines. (2016) 4:1–21. doi: 10.3390/vaccines4040041 PMC5192361 27834810

[280] Costa D Venè R Benelli R Romairone E Scabini S Catellani S. Targeting the epidermal growth factor receptor can counteract the inhibition of natural killer cell function exerted by colorectal tumor-associated fibroblasts. Front Immunol. (2018) 9:1150. doi: 10.3389/fimmu.2018.01150 29910806 PMC5992415

[281] Hanahan D Michielin O Pittet MJ. Convergent inducers and effectors of T cell paralysis in the tumour microenvironment. Nat Rev Cancer. (2025) 25:41–58. doi: 10.1038/s41568-024-00761-z 39448877

[282] Huang J Liu F Li C Liang X Li C Liu Y. Role of CD47 in tumor immunity: a potential target for combination therapy. Sci Rep. (2022) 12:1–11. doi: 10.1038/s41598-022-13764-3 35697717 PMC9192775

[283] Marquardt V Theruvath J Pauck D Picard D Qin N Blümel L. Tacedinaline (CI-994), a class I HDAC inhibitor, targets intrinsic tumor growth and leptomeningeal dissemination in MYC-driven medulloblastoma while making them susceptible to anti-CD47-induced macrophage phagocytosis via NF-kB-TGM2 driven tumor inflammat. J ImmunoTherapy Cancer. (2023) 11:e005871. doi: 10.1136/jitc-2022-005871 PMC9843227 36639156

[284] Marques RF Moreno DA da Silva L Leal LF de Paula FE Santana I. Digital expression profile of immune checkpoint genes in medulloblastomas identifies CD24 and CD276 as putative immunotherapy targets. Front Immunol. (2023) 14:1062856. doi: 10.3389/fimmu.2023.1062856 36825029 PMC9941636

[285] Yang SY Choi SA Lee JY Park AK Wang KC Phi JH. miR-192 suppresses leptomeningeal dissemination of medulloblastoma by modulating cell proliferation and anchoring through the regulation of DHFR, integrins, and CD47. Oncotarget. (2015) 6:43712–30. doi: 10.18632/oncotarget.6227 PMC4791261 26506238

[286] Northcott PA Korshunov A Witt H Hielscher T Eberhart CG Mack S. Medulloblastoma comprises four distinct molecular variants. J Clin Oncol. (2011) 29:1408–14. doi: 10.1200/JCO.2009.27.4324 PMC4874239 20823417

[287] Yang H Shao R Huang H Wang X Rong Z Lin Y. Engineering macrophages to phagocytose cancer cells by blocking the CD47/SIRPɑ axis. Cancer Med. (2019) 8:4245–53. doi: 10.1002/cam4.2332 PMC6675709 31183992

[288] Ni H Cao L Wu Z Wang L Zhou S Guo X. Combined strategies for effective cancer immunotherapy with a novel anti-CD47 monoclonal antibody. Cancer Immunol Immunother. (2022) 71:353–63. doi: 10.1007/s00262-021-02989-2 PMC10991210 34165607

[289] Barres BA. The mystery and magic of glia: a perspective on their roles in health and disease. Neuron. (2008) 60:430–40. doi: 10.1016/j.neuron.2008.10.013 18995817

[290] Cunningham C Dunne A Lopez-Rodriguez AB. Astrocytes: heterogeneous and dynamic phenotypes in neurodegeneration and innate immunity. Neuroscientist. (2019) 25:455–74. doi: 10.1177/1073858418809941 PMC6525076 30451065

[291] Kwon W Choi DJ Yu K Williamson MR Murali S Ko Y. Comparative transcriptomic analysis of cerebellar astrocytes across developmental stages and brain regions. Int J Mol Sci. (2024) 25. doi: 10.3390/ijms25021021 PMC10816327 38256095

[292] Verkhratsky A Butt A Li B Illes P Zorec R Semyanov A. Astrocytes in human central nervous system diseases: a frontier for new therapies. Signal Transduction Targeted Ther. (2023) 8:1021. doi: 10.1038/s41392-023-01628-9 PMC10570367 37828019

[293] Liddelow SA Guttenplan KA Clarke LE Bennett FC Bohlen CJ Schirmer L. Neurotoxic reactive astrocytes are induced by activated microglia. Nature. (2017) 541:481–7. doi: 10.1038/nature21029 PMC5404890 28099414

[294] Neal M Luo J Harischandra DS Gordon R Sarkar S Jin H. Prokineticin-2 promotes chemotaxis and alternative A2 reactivity of astrocytes. Glia. (2018) 66:2137–57. doi: 10.1002/glia.23467 PMC6240381 30277602

[295] Sofroniew MV. Astrocyte reactivity: subtypes, states, and functions in CNS innate immunity. Trends Immunol. (2020) 41:758–70. doi: 10.1016/j.it.2020.07.004 PMC7484257 32819810

[296] Bocchi R Thorwirth M Simon-Ebert T Koupourtidou C Clavreul S Kolf K. Astrocyte heterogeneity reveals region-specific astrogenesis in the white matter. Nat Neurosci. (2025) 28:457–69. doi: 10.1038/s41593-025-01878-6 PMC11893471 39994409

[297] Endo F Kasai A Soto JS Yu X Qu Z Hashimoto H. Molecular basis of astrocyte diversity and morphology across the CNS in health and disease. Science. (2022) 378:eadc9020. doi: 10.1126/science.adc9020 36378959 PMC9873482

[298] Jurga AM Paleczna M Kadluczka J Kuter KZ. Beyond the GFAP-astrocyte protein markers in the brain. Biomolecules. (2021) 11:1361. doi: 10.3390/biom11091361 34572572 PMC8468264

[299] Liu Y Yuelling LW Wang Y Du F Gordon RE O’Brien JA. Astrocytes promote medulloblastoma progression through hedgehog secretion. Cancer Res. (2017) 77:6692–703. doi: 10.1158/0008-5472.CAN-17-1463 PMC5759326 28986380

[300] Duarte TT Teixeira SA Gonzalez-Reyes L Reis RM. Decoding the roles of astrocytes and hedgehog signaling in medulloblastoma. Curr Oncol. (2021) 28:3058–70. doi: 10.3390/curroncol28040267 PMC8395412 34436033

[301] Cheng Y Franco-Barraza J Wang Y Zheng C Zhang L Qu Y. Sustained hedgehog signaling in medulloblastoma tumoroids is attributed to stromal astrocytes and astrocyte-derived extracellular matrix. Lab Invest. (2020) 100:1208–22. doi: 10.1038/s41374-020-0443-2 PMC7442735 32457352

[302] Qadeer ZA Weiss WA. A SHHecret target of relapsed medulloblastoma: Astrocytes. J Exp Med. (2021) 218:9–11. doi: 10.1084/jem.20211141 PMC8424466 34398189

[303] Li H Liu Y Liu Y Xu L Sun Z Zheng D. Tumor-associated astrocytes promote tumor progression of Sonic Hedgehog medulloblastoma by secreting lipocalin-2. Brain Pathol. (2024) 34:1–19. doi: 10.1111/bpa.13212 PMC10711256 37721122

[304] Liu H Liu H Sun Y O’Brien JA O’Brien JA Franco-Barraza J. Necroptotic astrocytes contribute to maintaining stemness of disseminated medulloblastoma through CCL2 secretion. Neuro-Oncology. (2020) 22:625–38. doi: 10.1093/neuonc/noz214 PMC7229261 31729527

[305] Eberhart C. Astrocytes: New stars in the medulloblastoma firmament. Neuro-Oncology. (2020) 22:587–9. doi: 10.1093/neuonc/noaa057 PMC7229242 32157298

[306] Schwalbe EC Lindsey JC Danilenko M Hill RM Crosier S Ryan SL. Molecular and clinical heterogeneity within MYC-family amplified medulloblastoma is associated with survival outcomes: A multicenter cohort study. Neuro Oncol. (2025) 27:222–36. doi: 10.1093/neuonc/noae178 PMC11726341 39377358

[307] Mynarek M Obrecht D Sill M Sturm D Kloth-Stachnau K Selt F. Identification of low and very high-risk patients with non-WNT/non-SHH medulloblastoma by improved clinico-molecular stratification of the HIT2000 and I-HIT-MED cohorts. Acta Neuropathol. (2023) 145:97–112. doi: 10.1007/s00401-022-02522-4 36459208 PMC9807480

[308] Sarvode S Gajjar A. Review of the impact of molecular analysis on the therapy of medulloblastoma. Pediatr Hematol Oncol J. (2023) 8:121–8. doi: 10.1016/j.phoj.2023.05.001

[309] Takenaka M Kuroda K Tanaka F. Adjuvant and neo-adjuvant therapy for non-small cell lung cancer without EGFR mutations or ALK rearrangements. Int J Clin Oncol. (2024) 30:215–28. doi: 10.1007/s10147-023-02459-y 38281195

[310] Dunkel IJ Doz F Foreman NK Hargrave D Lassaletta A André N. Nivolumab with or without ipilimumab in pediatric patients with high-grade CNS Malignancies: Safety, efficacy, biomarker, and pharmacokinetics-CheckMate 908. Neuro Oncol. (2023) 25:1530–45. doi: 10.1093/neuonc/noad031 PMC10398811 36808285

[311] Gorsi HS Malicki DM Barsan V Tumblin M Yeh-Nayre L Milburn M. Nivolumab in the treatment of recurrent or refractory pediatric brain tumors: A single institutional experience. J Pediatr Hematol Oncol. (2019) 41:e235–41. doi: 10.1097/MPH.0000000000001339 30681550

[312] Bockmayr M Mohme M Klauschen F Winkler B Budczies J Rutkowski S. Subgroup-specific immune and stromal microenvironment in medulloblastoma. OncoImmunology. (2018) 7:1–10. doi: 10.1080/2162402X.2018.1462430 PMC6140816 30228931

[313] Li C Zou H Xiong Z Xiong Y Miyagishima DF Wanggou S. Construction and validation of a 13-gene signature for prognosis prediction in medulloblastoma. Front Genet. (2020) 11:429. doi: 10.3389/fgene.2020.00429 32508873 PMC7249855

[314] Diao S Gu C Zhang H Yu C. Immune cell infiltration and cytokine secretion analysis reveal a non-inflammatory microenvironment of medulloblastoma. Oncol Lett. (2020) 20:1361. doi: 10.3892/ol.2020.12260 PMC7656115 33193857

[315] Murata D Mineharu Y Arakawa Y Liu B Tanji M Yamaguchi M. High programmed cell death 1 ligand-1 expression: association with CD8+ T-cell infiltration and poor prognosis in human medulloblastoma. J Neurosurg. (2018) 128:710–6. doi: 10.3171/2016.11.JNS16991 28474991

[316] Grabovska Y Mackay A O’Hare P Crosier S Finetti M Schwalbe EC. Pediatric pan-central nervous system tumor analysis of immune-cell infiltration identifies correlates of antitumor immunity. Nat Commun. (2020) 11:1–15. doi: 10.1038/s41467-020-18070-y 32859926 PMC7455736

[317] Majzner RG Simon JS Grosso JF Martinez D Pawel BR Santi M. Assessment of programmed death-ligand 1 expression and tumor-associated immune cells in pediatric cancer tissues. Cancer. (2017) 123:3807–15. doi: 10.1002/cncr.30724 28608950

[318] Velz J Freudenmann LK Medici G Dubbelaar M Mohme M Ghasemi DR. Mapping naturally presented T cell antigens in medulloblastoma based on integrative multi-omics. Nat Commun. (2025) 16:1364. doi: 10.1038/s41467-025-56268-0 39904979 PMC11794601

[319] Zhang W Zhu Y Liu H Zhang Y Liu H Adegboro AA. Pan-cancer evaluation of regulated cell death to predict overall survival and immune checkpoint inhibitor response. NPJ Precis Oncol. (2024) 8:77. doi: 10.1038/s41698-024-00570-5 38538696 PMC10973470

[320] Rezazadeh-Gavgani E Majidazar R Lotfinejad P Kazemi T Shamekh A. Immune checkpoint molecules: A review on pathways and immunotherapy implications. Immun Inflammation Dis. (2025) 13:e70196. doi: 10.1002/iid3.70196 PMC12004596 40243372

[321] Borgeaud M Sandoval J Obeid M Banna G Michielin O Addeo A. Novel targets for immune-checkpoint inhibition in cancer. Cancer Treat Rev. (2023) 120:102614. doi: 10.1016/j.ctrv.2023.102614 37603905

[322] Hernández-Prat A Rodriguez-Vida A Cardona L Qin M Arpí-Llucià O Soria-Jiménez L. Enhancing immunotherapy through PD-L1 upregulation: the promising combination of anti-PD-L1 plus mTOR inhibitors. Mol Oncol. (2025) 19:151–72. doi: 10.1002/1878-0261.13699 PMC11705730 39258533

[323] Song H Chen L Pan X Shen Y Ye M Wang G. Targeting tumor monocyte-intrinsic PD-L1 by rewiring STING signaling and enhancing STING agonist therapy. Cancer Cell. (2025) 43:503–518.e10. doi: 10.1016/j.ccell.2025.02.014 40068600

[324] Li Y Lim C Dismuke T Malawsky DS Oasa S Bruce ZC. Suppressing recurrence in Sonic Hedgehog subgroup medulloblastoma using the OLIG2 inhibitor CT-179. Nat Commun. (2025) 16:1091. doi: 10.1038/s41467-024-54861-3 39904981 PMC11794477

[325] Szu JI Tsigelny IF Wojcinski A Kesari S. Biological functions of the Olig gene family in brain cancer and therapeutic targeting. Front Neurosci. (2023) 17:1129434. doi: 10.3389/fnins.2023.1129434 37274223 PMC10232966

[326] Buffo A Vosko MR Ertürk D Hamann GF Jucker M Rowitch D. Expression pattern of the transcription factor Olig2 in response to brain injuries: Implications for neuronal repair. Proc Natl Acad Sci United States America. (2005) 102:18183–8. doi: 10.1073/pnas.0506535102 PMC1312388 16330768

[327] Meijer DH Kane MF Mehta S Liu H Harrington E Taylor CM. Separated at birth? The functional and molecular divergence of OLIG1 and OLIG2. Nat Rev Neurosci. (2012) 13:819–31. doi: 10.1038/nrn3386 PMC3733228 23165259

[328] Zhou Q Anderson DJ. The bHLH transcription factors OLIG2 and OLIG1 couple neuronal and glial subtype specification. Cell. (2002) 109:61–73. doi: 10.1016/s0092-8674(02)00677-3 11955447

[329] Visvanathan A Saulnier O Chen C Haldipur P Orisme W Delaidelli A. Early rhombic lip Protogenin+ve stem cells in a human-specific neurovascular niche initiate and maintain group 3 medulloblastoma. Cell. (2024) 187:4733–50. doi: 10.1016/j.cell.2024.06.011 PMC11707800 38971152

[330] Lasky JL Bradford KL Wang Y Pak Y Panosyan EH. Chemotherapy can synergize with adoptive immunotherapy to inhibit medulloblastoma growth. Anticancer Res. (2022) 42:1697–706. doi: 10.21873/anticanres.15646 35346988

[331] Petralia F Tignor N Reva B Koptyra M Chowdhury S Rykunov D. Integrated proteogenomic characterization across major histological types of pediatric brain cancer. Cell. (2020) 183:1962–1985.e31. doi: 10.1016/j.cell.2020.10.044 PMC8143193 33242424

[332] Cerami E Gao J Dogrusoz U Gross BE Sumer SO Aksoy BA. The cBio cancer genomics portal: an open platform for exploring multidimensional cancer genomics data. Cancer Discovery. (2112) 2:401–4. doi: 10.1158/2159-8290.CD-12-0095 PMC3956037 22588877

