# Supplementary material for: Medulloblastoma: biology and immunotherapy
Source: Front Immunol. 2025 Jul 3;16:1602930. doi: 10.3389/fimmu.2025.1602930 (PMC12267187; doi:10.3389/fimmu.2025.1602930)
Supplement: Supplementary file 1 [file DataSheet1.docx]

**Supplementary Figure 1. Analysis of public data of MB tumors for the expression of ligands of activating molecules present on anti-tumor effector cells.** (A). Major histocompatibility complex class I-related (MR1) expression on a cohort of MB specimens from data previously reported (1). (B). Heatmap of the analysis of expression on MB of several ligands for molecules expressed on effector lymphocytes such as BTN1A1, BTN3A1, BTN3A3, BTN3A2, BTN2A2, BTN2A1, BTNLB, BTNL2, BTNL9, BTNL3, CD1A, CD1B. CD1C, CD1D, MR1, ULBP1, ULBP2, ULBP3, MICB, BAG6 and NCR3LG1 ligand for NKp30 activating receptor. Data are from the medulloblastoma gene expression array (GSE85217) (2). (C) gene expression of the same molecules grouped for each MB subtype. Medulloblastoma gene expression array (GSE85217) [1,2] was analyzed with the R software Gene heatmap were produced with Complex Heatmap R package [3, 4]. Gene heatmaps were produced with Complex Heatmap R package samples defined as "High" had tumor purity levels lower than the first quantile, while "Low" had values higher than the third quantile.

**Supplementary Figure 2. Analysis of public data of MB on the expression of molecules relevant for immune recognition.** (A) Heatmap and (B) grouped gene expression on MB specimens of some HLA (DQB1, DQA1) immune checkpoint receptors/ligands (CTLA4, PDCD1, PDCD1LG2, CD274, HAVCR2, CEACAM1, LGALS3, FGL1 ligand of LAG3), costimulatory molecules (CD80, CD86). (C) Heatmap and (D) grouped gene expression on MB of MHC-Class I and II molecules (HLA-A, HLA-B, B2M, DQB1, DRA1, DRB1), ICAM1, KIR3DL1, IFNG, IFNA1, IFNB1 and TNF. The data set are from the reference 2.

**Supplementary Figure 3. Analysis of public data of infiltrating lymphocytes in MB.** (A) Heatmap and (B) infiltrating immune cell subsets for each MB subtype. The data are from reference 2. Infiltrate composition was computed with xcell in the immunodeconv R package [6, 7].

References cited

1.Cornel AM, van der Sman L, van Dinter JT, Arrabito M, et al. Targeting pediatric cancers via T-cell recognition of the monomorphic MHC class I-related protein MR1. J Immunother Cancer. 2024 Mar 21;12(3):e007538. doi: 10.1136/jitc-2023-007538.

2 Cavalli FMG, Remke M, Rampasek L, Peacock J, Shih DJH, Luu B, Garzia L, Torchia J, Nor C, Morrissy AS, Agnihotri S, Thompson YY, Kuzan-Fischer CM, Farooq H, Isaev K, Daniels C, Cho BK, Kim SK, Wang KC, Lee JY, Grajkowska WA, Perek-Polnik M, Vasiljevic A, Faure-Conter C, Jouvet A, Giannini C, Nageswara Rao AA, Li KKW, Ng HK, Eberhart CG, Pollack IF, Hamilton RL, Gillespie GY, Olson JM, Leary S, Weiss WA, Lach B, Chambless LB, Thompson RC, Cooper MK, Vibhakar R, Hauser P, van Veelen MC, Kros JM, French PJ, Ra YS, Kumabe T, López-Aguilar E, Zitterbart K, Sterba J, Finocchiaro G, Massimino M, Van Meir EG, Osuka S, Shofuda T, Klekner A, Zollo M, Leonard JR, Rubin JB, Jabado N, Albrecht S, Mora J, Van Meter TE, Jung S, Moore AS, Hallahan AR, Chan JA, Tirapelli DPC, Carlotti CG, Fouladi M, Pimentel J, Faria CC, Saad AG, Massimi L, Liau LM, Wheeler H, Nakamura H, Elbabaa SK, Perezpeña-Diazconti M, Chico Ponce de León F, Robinson S, Zapotocky M, Lassaletta A, Huang A, Hawkins CE, Tabori U, Bouffet E, Bartels U, Dirks PB, Rutka JT, Bader GD, Reimand J, Goldenberg A, Ramaswamy V, Taylor MD. Intertumoral Heterogeneity within Medulloblastoma Subgroups. Cancer Cell. 2017 Jun 12;31(6):737-754.e6. doi: 10.1016/j.ccell.2017.05.005. PMID: 28609654; PMCID: PMC6163053.

3 Ramaswamy V, Taylor MD. Bioinformatic Strategies for the Genomic and Epigenomic Characterization of Brain Tumors. Methods Mol Biol. 2019; 1869:37-56. doi: 10.1007/978-1-4939-8805-1_4. PMID: 30324512.Mol Biol 2019;1869:37-56. PMID: 30324512

4 Gu Z, Eils R, Schlesner M. Complex heatmaps reveal patterns and correlations in multidimensional genomic data. Bioinformatics. 2016 Sep 15;32(18):2847-9. doi: 10.1093/bioinformatics/btw313. Epub 2016 May 20. PMID: 27207943.

5 Gu Z. Complex heatmap visualization. Imeta. 2022 Aug 1;1(3):e43. doi: 10.1002/imt2.43. PMID: 38868715; PMCID: PMC10989952.

6 Aran D, Hu Z, Butte AJ. xCell: digitally portraying the tissue cellular heterogeneity landscape. Genome Biol. 2017 Nov 15;18(1):220. doi: 10.1186/s13059-017-1349-1. PMID: 29141660; PMCID: PMC5688663.

7 Sturm G, Finotello F, List M. Immunedeconv: An R Package for Unified Access to Computational Methods for Estimating Immune Cell Fractions from Bulk RNA-Sequencing Data. Methods Mol Biol. 2020; 2120:223-232. doi: 10.1007/978-1-0716-0327-7_16. PMID: 32124323.
