# Supplementary figures and images for: Medulloblastoma: biology and immunotherapy
Source: Front Immunol. 2025 Jul 3;16:1602930. doi: 10.3389/fimmu.2025.1602930 (PMC12267187; doi:10.3389/fimmu.2025.1602930)

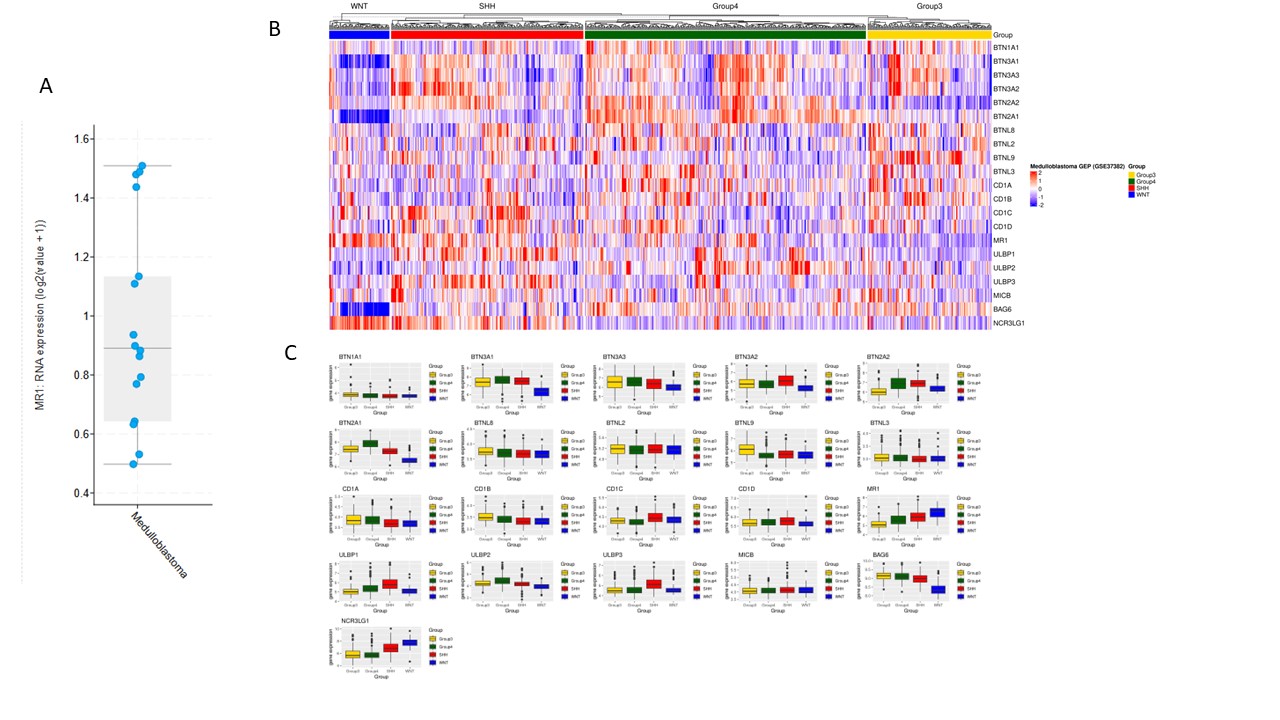

Supplement: Supplementary file 2 [file Image1.jpeg]

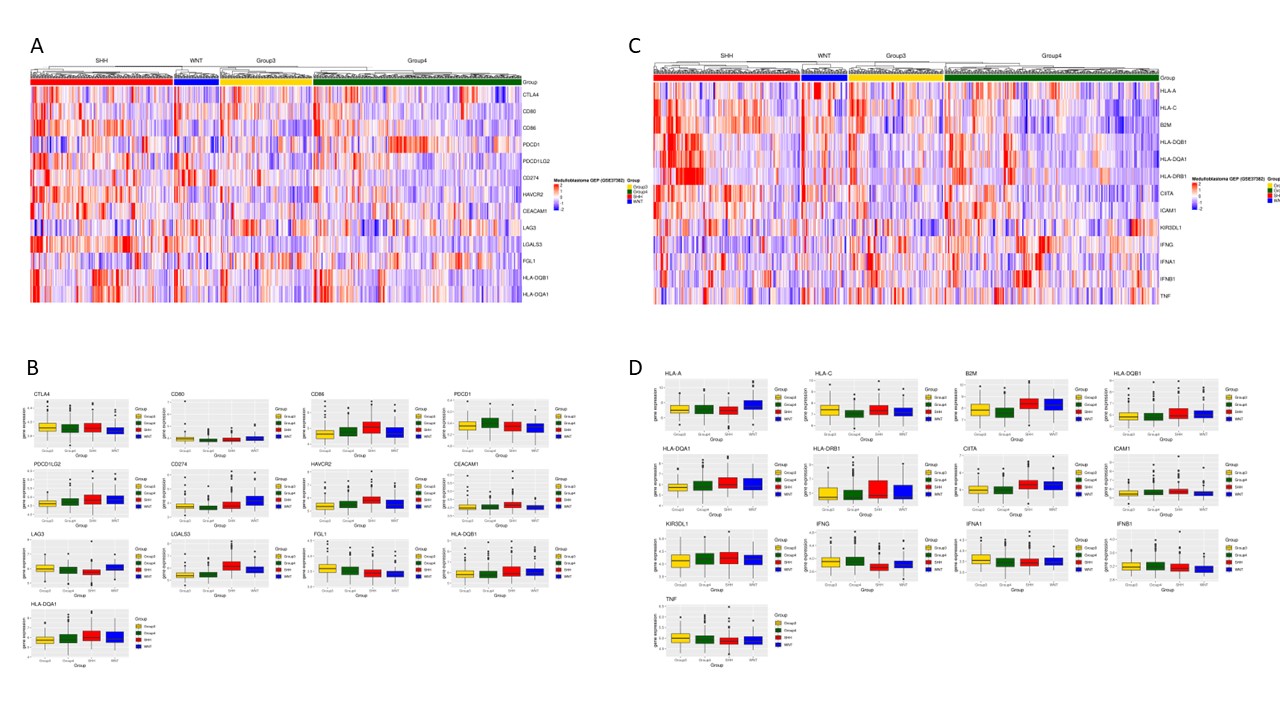

Supplement: Supplementary file 3 [file Image2.jpeg]

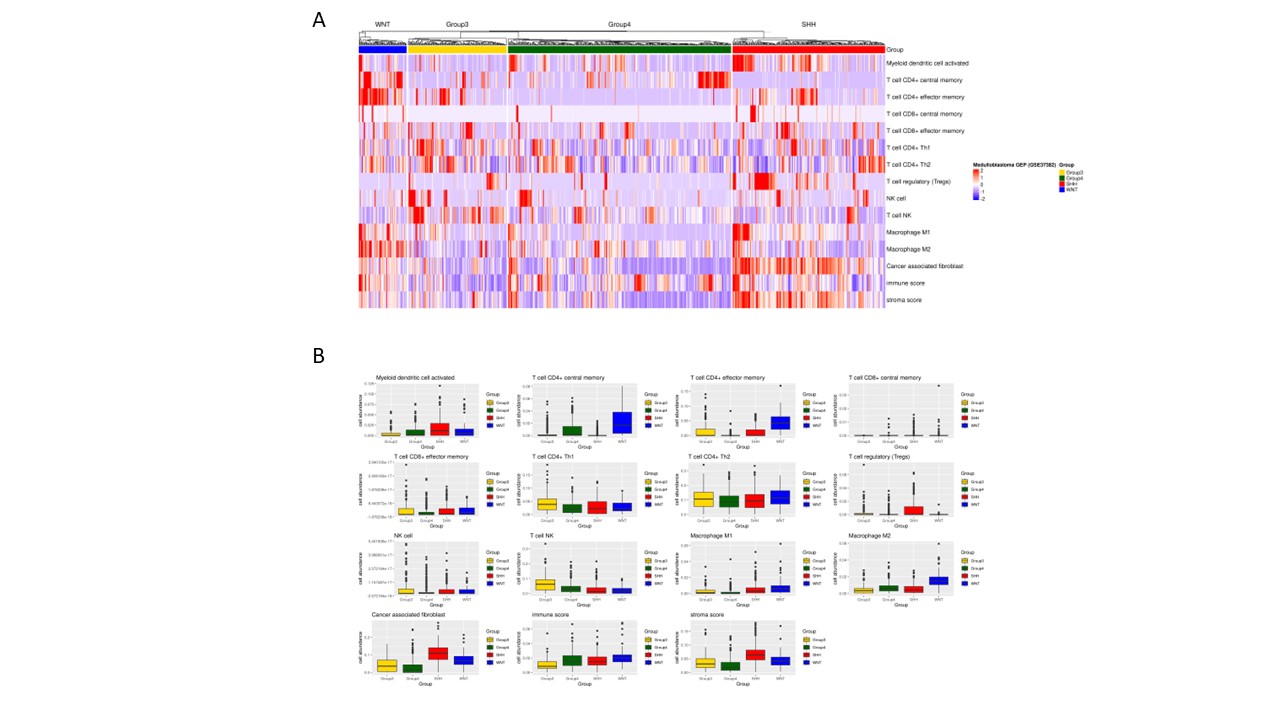

Supplement: Supplementary file 4 [file Image3.jpeg]
